# Nematic and chiral superconductivity emerging within the loop-current phase in kagome metals

**DOI:** 10.1038/s41467-026-75259-3

**Published:** 2026-07-27

**Authors:** Rina Tazai, Youichi Yamakawa, Hiroshi Kontani

**Affiliations:** 1 Center for Emergent Matter Science, RIKEN, Wako, Japan; 2 Department of Physics, Nagoya University, Nagoya, Japan

**Keywords:** Superconducting properties and materials, Electronic properties and materials, Topological matter

## Abstract

The kagome metals *A*V_3_Sb_5_ (*A* = Cs, Rb, K) host multiple symmetry-breaking phases, including charge-density-wave and loop-current orders, and exhibit highly exotic superconductivity with pronounced nematicity and chirality. Remarkably, even dilute impurities transform this exotic superconducting state into an isotropic *s*-wave state. These observations pose a challenge to existing theoretical scenarios. We show that loop-current order induces nematic chiral *d*-wave superconductivity in kagome metals. The loop-current-induced orbital magnetization (OM) stabilizes one chiral superconducting channels. This OM-chirality coupling mechanism is generic and applies to pairing driven by either attractive or repulsive interactions. Furthermore, coexisting loop-current and bond orders give rise to pronounced nematic chiral superconductivity even for an almost *C*_6_-symmetric Fermi surface. For attractive pairing, dilute impurities suppress the chiral state and restore a conventional *s*-wave phase, as observed experimentally. The theory further predicts a robust 2 × 2 pair-density modulation. This study provides key insights into time-reversal-symmetry-breaking exotic superconductivity in kagome metals.

## Abstract (web summary)

The authors show that the loop-current in the normal state gives rise to nematic chiral d-wave superconductivity in AV3Sb5 kagome metals, further enhanced by bond order. For attractive pairing, weak impurity scattering suppresses the chiral state and restores a conventional s-wave phase, as observed experimentally.

## Introduction

In strongly correlated metals, a variety of exotic quantum phase transitions have been discovered over the years, leading to intriguing symmetry-breaking states. Recently discovered kagome metal *A*V_3_Sb_5_ (*A* = Cs, Rb, K)^1,2^ displays remarkable electronic states arising from a sequence of quantum phases, including the charge bond-order (BO)^3,4^ and *s*-wave superconductivity with finite impurities^5,6^. In addition, *C*_6_-symmetry-breaking nematic states emerge in the normal state^3,4,7,8^ and superconducting (SC) state^9–11^. Particularly, time-reversal symmetry (TRS) breaking charge loop-current (LC) state, unrelated to spin polarization, has garnered significant attention^12–18^.

**Fig. 1:**
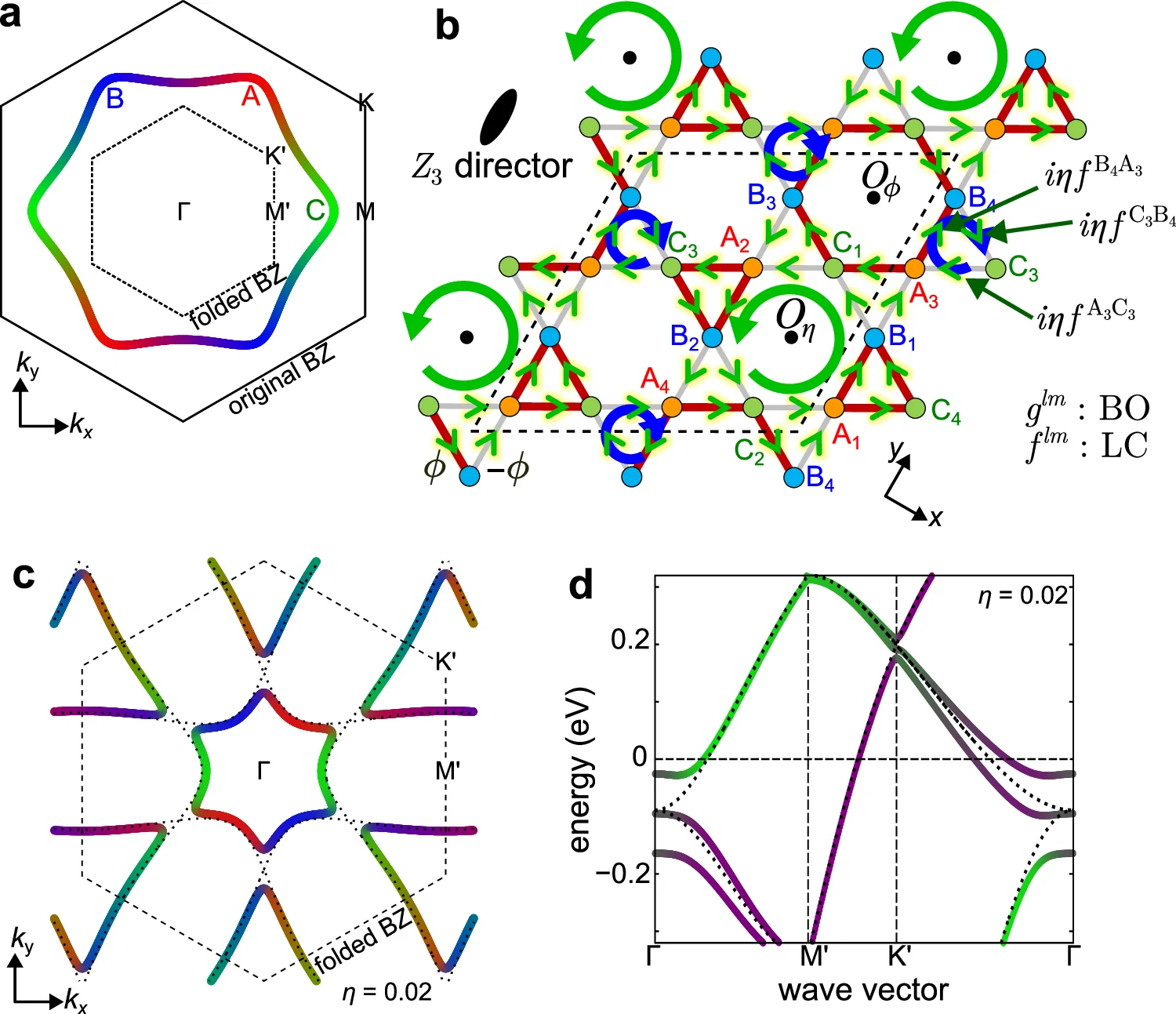
Kagome lattice model with 2 × 2 BO and LC. **a** Fermi surfaces without density-waves in the original BZ. Red, blue, and green colors represent the weight of the sublattices A, B, and C, respectively. **b** 2 × 2 LC in the extended unit cell. *iη* is the LC order parameter, and *f*^*lm*^ = − *f*^*ml*^ is the LC form factor. *ϕ* is the BO order parameter, and *g*^*lm*^ = *g*^*ml*^ is the BO form factor. (Hereafter, we refer to *ϕ* > 0 (*ϕ* < 0) as the TrH (SoD) BO; see the Supplementary Note A for details). The coexistence between the BO and LC leads to the nematic state, whose director is along *y* (or equivalently *x*) axis. The 2 × 2 unit cell is composed of 12 sites denoted as *M*_*μ*_, where *M *= A, B, C and *μ* = 1–4. **c** Fermi surfaces and **d** band-structure under finite LC (*η* = 0.02, *ϕ* = 0) in the folded BZ for *n* = 11.84. Dotted lines represent the original FS and bandstructure at *η* = 0. The triple degenerate bands at *E* = *E*_vHS_ at the *Γ* point are lifted by the LC order.

The LC order parameter involves a “pure imaginary” modulation in the hopping integral^19–22^. The microscopic mechanism of the LC order has been investigated in refs. ^23–28^. The LC is mediated by the BO quantum fluctuations^29^ in kagome metals. Figure 1a shows the original Fermi surface (FS), in the absence of the BO and LC, in the kagome lattice model denoted in Fig. 1b. The 2 × 2 BO and 2 × 2 LC states are schematically shown in Figs. 1b and S1 in the Supplementary Note A (Supplementary Information). In these figures, the BO center (*O*_*ϕ*_) and the LC center (*O*_*η*_) are out-of-phase^29,30^. Then, the LC + BO coexistence becomes nematic, which is predicted by the Ginzburg–Landau (GL) free energy theory for *T* < *T*_LC_ ≪ *T*_BO_^29,30^, and has been experimentally observed^15^.

The chiral LC phase accompanies finite uniform orbital magnetization (OM) *M*_orb_, which can be switched by a small magnetic field *B*_*z*_, as evidenced by the anomalous Hall effect^31^, electronic magneto-chiral anisotropy (eMChA)^32,33^, and magnetic torque^13^ measurements. Magnetotropic susceptibility measurements reveal a small but heavily anisotropic *M*_orb_ with tiny hysteresis^18^. Another direct piece of evidence of LC comes from scanning tunneling microscopy (STM) measurements: the chirality in the quasiparticle interference (QPI) is switched by *B*_*z*_ in both *A *= Cs and *A *= K compounds^12,14–17^, which is naturally induced by the LC^34,35^. In addition, the internal local magnetic field in the LC phase has been reported by *μ*SR^36,37^ measurements. In contrast, Kerr-effect studies reported the absence of LC^38^. Recently, magnetic circular dichroism ARPES has measured the orbital magnetic moments mainly on the *xz*-orbital band in CsV_3_Sb_5_^39,40^. Also, the SC diode polarity reflects the normal-state LC chirality in ref. ^41^.

Furthermore, striking exotic SC states with nontrivial symmetry breaking appear below *T*_c_ (=1–3 K) in kagome metals, as revealed by recent intensive experiments. For example, STM measurement^14^ reveals the emergence of (i) TRS breaking the chiral SC state that lacks in-plane mirror symmetry. This finding is further supported by the observations of the SC diode effect^41,42^ and giant thermal Hall effect^43^ under zero magnetic field. In addition, (ii) pronounced nematic anisotropy in the SC gap has been observed^9–11^. Moreover, (iii) prominent modulation in the SC gap, called the pair-density-modulation (PDM), has been observed^14,44^ and analyzed theoretically^14,45^. Unexpectedly, such exotic pairing states transform into a (iv) conventional *s*-wave state by introducing a small amount of disorder^5,6,46^. At present, the nature of exotic SC states with multiple symmetry breakings in kagome metals—likely closely linked to the LC order due to the hierarchy *T*_c_ ≪ *T*_LC_—remains a major unresolved problem.

In this paper, we propose a generic mechanism for nematic and chiral superconductivity, accompanied by significant PDM, in the LC phase of kagome metals. One of the two chiral *d*-wave (*d*^±^ wave) SC states is stabilized by the LC-induced uniform OM, and this “OM-chirality coupling mechanism” becomes relevant at low temperatures. When the LC order appears in the BO phase, a prominent nematic chiral SC state is realized due to the mixture of the chiral *d*-wave and *s*-wave states. For attractive pairing interactions, the present SC state is smoothly transformed into a conventional *s*-wave state by introducing dilute impurities, as discussed for Fe-based superconductors^47^. Moreover, the present mechanism yields a prominent 2 × 2 PDM. This study provides insights into the chiral SC states arising from the LC phase in kagome metals, such as the SC diode effect and the giant thermal Hall effect under zero magnetic field.

The microscopic mechanism of the exotic multiple quantum phase transitions has been studied very actively. The mean-field theories, as well as the renormalization group theories based on the (extended) Hubbard models, have been performed in refs. ^8,23,25,27–29,48–54^. It was shown that the star-of-David BO state is driven by the paramagnon-interference^8^, which is described by the Aslamazov-Larkin vertex corrections that are dropped in the mean-field type approximations^55–59^. Note that the BO fluctuations mediate not only attractive SC interaction but also the LC order with broken TRS^8,29^.

## Results

### Kagome lattice model with LC + BO order parameters

First, we introduce the kagome lattice model: in the Fermi surface in Fig. 1a, each van Hove singularity (vHS) on the original Brillouin zone (BZ) is associated with a single sublattice (A, B, or C), known as sublattice interference^48,50^. Now, we introduce the LC order parameter, described as ${\rm\delta }{t}_{ij}^{{{\rm{c}}}}=i\eta {f}^{ij}$ with *f*^*ij*^ = − *f*^*ji*^ = + 1 or −1, which is depicted in Figs. 1b and S1^30^. Also, the BO parameter *ϕ* is described as ${\rm\delta }{t}_{ij}^{{{\rm{b}}}}=\phi {g}^{ij}$, where *g*^*ij*^ = *g*^*ji*^ = + 1 or −1 for the nearest-neighbor sites (*i*, *j*). The present LC + BO coexisting state breaks the *C*_6_ symmetry because the BO center (*O*_*ϕ*_) and the LC center (*O*_*η*_) are out-of-phase^29,30^: see Section “Z3 nematic LC + BO coexisting state” of the Methods and the Supplementary Note A (Supplementary Information) for details. This *Z*_3_ nematic LC +  BO coexistence is energetically favorable according to the GL free energy theory for *T* < *T*_LC_ ≪ *T*_BO_^29,30^.

### SC gap equation for the kagome lattice model with 2 × 2 density waves

In this paper, we set the hopping integrals to *t* = − 0.8 eV and ${t}^{{\prime} }/t=0.16$ in order to reproduce the shape of the large *d*_*xz*_-orbital Fermi surface, the so-called “p-type” Fermi surface, with gentle negative curvature near the M point reported in ARPES studies. See the Supplementary Note E (Supplementary Information) for a comparison of the band structure of the present model with that obtained from ab initio calculations. In this study, we set the electron filling to *n* ≈ 11.8 to obtain a realistic Fermi-surface size and to place the *d*_*xz*_-orbital vHS energy at *E*_vHS_ − *μ* = − 0.05 ~ − 0.1 eV, consistent with observations in AV_3_Sb_5_ (A = K, Rb, Cs). Unless otherwise noted, energy is given in units of eV hereafter.

 Figure 1c, d denote the folded Fermi surfaces and band structure under finite LC (*η* = 0.029, *ϕ* = 0) in the folded BZ, respectively. Here, a single shallow electron-type or hole-type Fermi pocket emerges. It is caused by the LC-induced hybridization among three vHS points at the *Γ* point, which are originally located at M points in the original BZ. This shallow Fermi pocket has a large DOS and exhibits prominent Berry curvature due to the LC order. Its unfolded Fermi surface is presented in the Supplementary Note G (Supplementary Information). We will discuss the significant role of this Fermi pocket in chiral superconductivity.

In kagome metals *A*V_3_Sb_5_ (*A* = Cs, Rb, K), an *s*-wave superconducting state without sign reversal is realized in the presence of dense impurities^5,6,46^, mediated by an attractive pairing interaction arising from charge-channel fluctuations, including BO fluctuations^8^. Taking this into account, we solve the following gap equation with on-site attractive interaction *g*(>0), in the present study:

1 $$\lambda {\Delta }_{m}=g{\sum}_{l}{\Gamma }_{ml}{\Delta }_{l},$$

2 $${\Gamma }_{ml}=\frac{T}{N}{\sum}_{{{\bf{k}}},n}{G}_{{{\bf{k}}}}^{ml}({\epsilon }_{n}){G}_{-{{\bf{k}}}}^{ml}(-{\epsilon }_{n}){\rm\Theta }({\epsilon }_{n};\Omega ),$$

where *λ* is the eigenvalue and Δ_*m*_ is the local gap function for sublattice *m* (=1–12). *λ* reaches unity at *T* = *T*_c_. *Γ*_*ml*_ represents the pair-hopping amplitude from sublattice *l* to *m*. In Eq. 2, ${\hat{G}}_{{{\bf{k}}}}({\epsilon }_{n})$ is the Green function in the presence of the LC (*η*) and BO (*ϕ*) with Matsubara frequency *ϵ*_*n*_ = *πT*(2*n* + 1). ${\rm\Theta }({\epsilon }_{n};\Omega )={\Omega }^{2}/({(| {\epsilon }_{n}| -\pi T)}^{2}+{\Omega }^{2})$ is the cutoff function with *Ω*; we set *Ω* = 0.01 in the present paper. Here, we express ${\Gamma }_{ml}\equiv {\Gamma }_{ml}^{0}+{\gamma }_{ml}$: The first term is the real function for *η* = *ϕ* = 0, and the second term is ${\gamma }_{ml}\equiv {\gamma }_{ml}^{{\prime} }+i{\gamma }_{ml}^{{\prime\prime} }$, where ${\gamma }^{{\prime} }$ (*γ*^*″*^) denote the real (imaginary) parts. Note that *Γ*_*mm*_ ≫ ∣*Γ*_*ml*_∣ (*m* ≠ *l*) due to the sublattice interference^48,50^.

In Eq. 1, we assume a zero Cooper-pair momentum **Q**. The validity of this assumption is verified in the LC + BO phase for *ϕ*, *η* ≲ 0.05.

### Chiral *d*-wave superconductivity under a pure LC phase

**Fig. 2:**
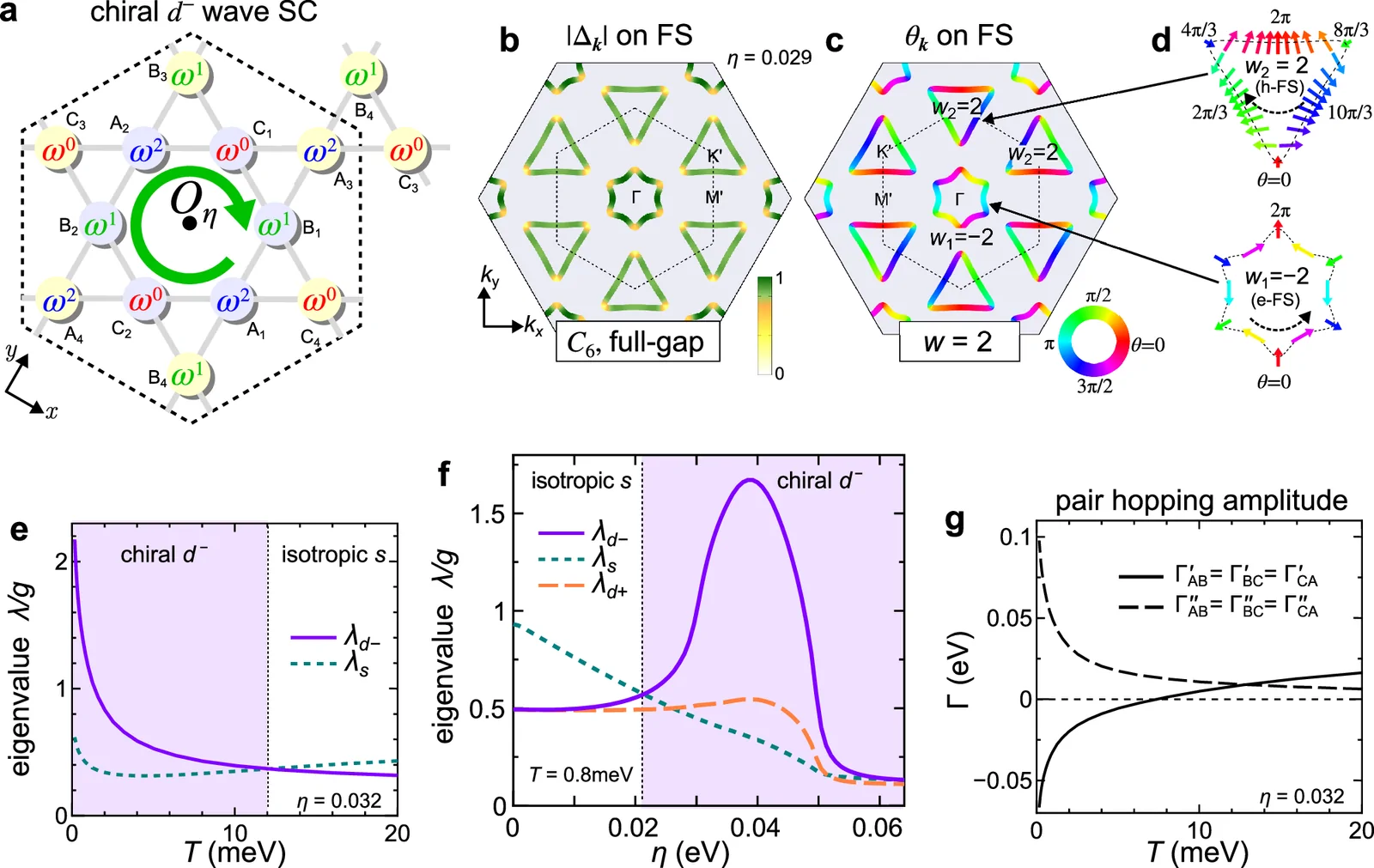
chiral *d*-wave superconductivity under a pure LC phase. **a** Chiral *d*-wave SC gap (*χ*_*d*_ = − 1) under the LC order, where *ω* = *e*^*i*2*π*/3^. (*s*-wave state corresponds to *ω* = 1) Here, the LDOS ${\rho }_{{M}_{\mu }}(\epsilon )$ and $| {\Delta }_{{M}_{\mu }}|$ take equal values at *μ* = 1 and 2, and also at *μ* = 3 and 4. **b** ∣Δ_**k**_∣ and **c** ${\theta }_{{{\bf{k}}}}\equiv \arctan ({\Delta }_{{{\bf{k}}}}^{{\prime\prime} }/\Delta {\prime} )$ on the Fermi surface for chiral *d*-wave state for *η* = 0.029 at *T* = 0.8 meV. *w*_1_ (*w*_2_) is the winding number of the SC gap around *Γ* (${K}^{{\prime} }$) point. The total winding number is *w* = *w*_1_ + 2*w*_2_. **d** Vector representation of *θ*_**k**_. Upward arrow corresponds to *θ* = 0. **e** Two largest eigenvalues as functions of *T* for *η* = 0.032. The *d*^±^ wave state with *T*_c_ ~ 2 meV is realized for *g* ~ 1.2. **f** Three largest eigenvalues as functions of *η* at *T* = 0.8 meV. At $\eta=0,\,{\lambda }_{{d}^{+}}={\lambda }_{{d}^{-}}$ and ${\lambda }_{s} > {\lambda }_{{d}^{\pm }}$. With $\eta > 0,\,{\lambda }_{{d}^{-}}$ gradually increases for finite *η*, and chiral *d*^−^ wave state is realized for *η* ≳ 0.02. **g** Complex off-diagonal kernel functions *Γ*_AB_, *Γ*_BC_, and *Γ*_CA_ as functions of *T*. Here, A ≡ *A*_3_, B ≡ *B*_4_, and C ≡ *C*_3_.

Hereafter, we express the twelve sublattices (*l* = 1–12) as *M*_*μ*_, where *M *= A, B, or C and *μ* = 1–4; as denoted in Fig. 2a: *μ* = 1 and 2 denote the six sites surrounding the LC center *O*_*η*_. That is, *μ* = 1, 2(3, 4) denote the sites participating in the circulating current on a hexagonal (triangular) plaquette. Then, the SC gap function is expressed as ${\Delta }_{{M}_{\mu }}$, and we introduce the notation ${{{\Delta }}}_{\mu }\equiv ({\Delta }_{{{{\rm{A}}}}_{\mu }},{\Delta }_{{{{\rm{B}}}}_{\mu }},{\Delta }_{{{{\rm{C}}}}_{\mu }})$. In the present study, the 2 × 2 PDM ∣Δ_PDM_∣ ≠ 0 can be prominent in the 2 × 2 density-wave phases; see Section “2 × 2 pair-density-modulation” of the Methods and the Supplementary Note A (Supplementary Information) for details.

First, we analyze the gap equation for *ϕ* = 0 (without BO). When *η* is zero, a plane *s*-wave SC state is obtained as the largest eigenvalue. The second-largest (*χ*_*d*_ = ± 1) eigenvalue corresponds to the doubly degenerate chiral *d*^±^ wave solutions. This double degeneracy is lifted when *η* is finite. In this paper, we reveal that a single chiral SC state (*χ*_*d*_ = + 1 or −1) can take over the *s*-wave SC state in the LC phase, because *Γ*_*ml*_ acquires an effective Aharonov-Bohm phase factor originating from the LC order *η* ≠ 0. The obtained *d*^−^ wave state Δ_*μ*_ ∝ (1, *ω*^2^, *ω*), which corresponds to *χ*_*d*_ = − 1, is depicted in Fig. 2a in real space. The chirality *χ*_*d*_ is reversed by changing *η* to −*η*. The gap function on the *b*-th Fermi momentum **k** is given as ${\Delta }_{b,{{\bf{k}}}}={\sum }_{m}{w}_{{{\bf{k}}}}^{m,b}{\Delta }_{m}$, where ${\omega }_{{{\bf{k}}}}^{m,b}$ is the weight of *m*-th sublattice on the *b*-th band. Hereafter, we omit the index *b*, as it is uniquely determined for a given Fermi momentum **k**.

In the chiral *d*-wave state, ${\Delta }_{{{\bf{k}}}}\equiv {\Delta }_{{{\bf{k}}}}^{{\prime} }+i{\Delta }_{{{\bf{k}}}}^{{\prime\prime} }$ is a complex-valued function of **k**. Figure 2b, c show the obtained ∣Δ_**k**_∣ and ${\theta }_{{{\bf{k}}}}\equiv \arctan ({\Delta }_{{{\bf{k}}}}^{{\prime\prime} }/{\Delta }_{{{\bf{k}}}}^{{\prime} })$ on the Fermi surface for the chiral *d*-wave state, respectively, for *η* = 0.029 at *T* = 0.8 meV. Here, ∣Δ_**k**_∣ exhibits a sizable dependence on **k** due to the phase difference of Δ_*l*_ (*l* = 1 − 12). The vector representation of *θ*_**k**_ for a single Fermi pocket is given in Fig. 2d. *w*_1_ (*w*_2_) denote the winding number of the SC gap on the electron-type (hole-type) FS, evaluated in the anticlockwise (clockwise) direction. The total winding number, *w* = *w*_1_ + 2*w*_2_, coincides with the skyrmion number $\frac{1}{4\pi }{\sum }_{b}\int\,d{k}_{x}d{k}_{y}{{{\bf{n}}}}_{b}({\partial }_{{k}_{x}}{{{\bf{n}}}}_{b}\times {\partial }_{{k}_{y}}{{{\bf{n}}}}_{b})$, with the unit vector ${{{\bf{n}}}}_{b,{{\bf{k}}}}=({\Delta }_{b,{{\bf{k}}}}^{{\prime} },{\Delta }_{b,{{\bf{k}}}}^{{\prime\prime} },\mu -{\epsilon }_{b,{{\bf{k}}}})/\sqrt{| {\Delta }_{b,{{\bf{k}}}}{| }^{2}+{(\mu -{\epsilon }_{b,{{\bf{k}}}})}^{2}}$. This quantity is equivalent to the Chern number $C=\frac{1}{2\pi }{\sum }_{b}\int\,d{k}_{x}d{k}_{y}{\Omega }_{{{\bf{k}}}}^{b}$ of the occupied Bogoliubov bands, when the Berry curvature of the normal-state bands in the LC phase is neglected.

Figure 2e shows the largest and second-largest eigenvalues as functions of *T* for *η* = 0.032. Notably, the obtained *λ*_*d*_ drastically increases for *T* < 5 meV. Therefore, a chiral *d*^−^ wave state with a transition temperature *T*_c_ ≪ 4 meV is realized for *g* < 1.7. (Note that *λ*_*x*_ in the higher *T* region (≳0.01) is overestimated, as the pairing constant *g* due to electron correlations should decrease with increasing *T*). Figure 2f shows the three largest eigenvalues as functions of *η* at *T* = 0.8 meV. At *η* = 0, the relation *λ*_*s*_ > *λ*_*d*_^+^ = *λ*_*d*_^−^ holds. As *λ*_*d*_^−^ gradually increases with *η*, the *d*^−^ wave state emerges for *η* ≳ 0.02. Thus, the *T*_c_ of the *d*^−^ wave state is enhanced by the LC order. The LC-induced enhancement of the chiral *d*-wave state originates from the inter-sublattice (*l* ≠ *m*) imaginary pair-hopping amplitude *Γ*_*lm*_, which is shown in Fig. 2g: in the LC phase, the magnitude of ${{\rm{Im}}}{\Gamma }_{{{\rm{lm}}}}$ (*l* ≠ *m*) is comparable to its real part, and ${{\rm{Re}}}{\Gamma }_{{{\rm{lm}}}} < 0$ at low temperatures. This LC-induced complex pair-hopping gives rise to the chiral SC, as we will discuss later.

### Inter-sublattice complex pair-hopping mechanism in the LC phase

Here, we discuss why chiral *d*-wave SC is realized in the LC phase due to the charge-channel attractive pairing interaction. The LC-induced band-hybridization gap suppresses the diagonal kernel function ${\Gamma }_{mm}~N(0){\mathrm{log}}({\rm\Omega }/T)$, leading to the reduction of the eigenvalue *λ*. (Here, *N*(*ϵ*) is the density-of-states). However, *Γ*_*ml*_ with *m* ≠ *l* becomes a complex functions for *η* ≠ 0, and its magnitude is drastically magnified at low temperatures, as shown in Fig. 2g. Then, the phase factor $\theta {{\prime} }_{ml}\equiv \arctan ({\Gamma }_{ml}^{{\prime\prime} }/{\Gamma }_{ml}^{{\prime} })$ prefers the phase difference between Δ_*m*_ and Δ_*l*_, working as the inter-sublattice “complex Josephson coupling”. For this reason, the chiral *d*-wave SC state emerges in the LC phase. A derivation of the Ginzburg–Landau free energy describing the LC–chiral SC coupling, based on the Green function method, is given in the Supplementary Note H (Supplementary Information).

A more intuitive physical picture is provided by the “OM-chirality coupling mechanism” between *M*_orb_ and chiral superconductivity. It is captured by the GL free energy ${F}_{{{\rm{OM}}}-{{\rm{ch}}}}=-{\bar{M}}_{{{\rm{orb}}}}\left(| {\Delta }^{d-}{| }^{2}-| {\Delta }^{d+}{| }^{2}\right)$, where the GL coefficient ${\bar{M}}_{{{\rm{orb}}}}$ has a functional form similar to *M*_orb_; see Supplementary Note H for details (Supplementary Information). In the present model, ∣*M*_orb_∣ = 10^−4^–10^−3^ [*μ*_*B*_] per V atom for *ϕ*, *η* = 10–20 meV; see Supplementary Note H (Supplementary Information). Then, the energy splitting between finite angular momenta (such as $\left\vert {l}_{z}=\pm 1\right\rangle$) is estimated as Δ*E* ~ *M*_orb_/*N*(0) = 0.1–1 meV, where *N*(0) ~ 1 [eV^−1^] is the density-of-states. The estimated Δ*E* is sufficiently large to induce a significant splitting between *λ*_*d*_^+^ and *λ*_*d*_^−^ when *T*_c_ ~ 1 meV. Thus, chiral Cooper pairs with orbital angular momentum are stabilized under the LC-induced effective *M*_orb_.

To verify these intuitive considerations, we analyze a simplified gap equation for three original sublattices A (=A_3_), B (=B_4_), and C (=C_3_), by neglecting the *μ*-dependence of ${\Delta }_{{M}_{\mu }}$: with reference to Fig. 2g, and taking into account that ${\Gamma }_{{M}_{\mu }{L}_{\nu }}$ (*M*, *L* = A, B, C) is nearly independent of *μ* and *ν* as verified in the Supplementary Note B, we introduce the following simple 3 × 3 complex kernel functions:

3 $$g\hat{\bar{\Gamma }}=\left(\begin{array}{ccc}{\lambda }_{0}&a-ib&a+ib\\ a+ib&{\lambda }_{0}&a-ib\\ a-ib&a+ib&{\lambda }_{0}\end{array}\right),$$

where *a* and *b* are real constants. (Note that ${\hat{\bar{{\Gamma }}}}_{ML}\approx 4{\Gamma }_{{{M}}_{{\mu }}{{L}}_{{\nu }}}$). The eigenvalues and gap functions are given as

4 $$\cdot \lambda={\lambda }_{0}+2a:{{\boldsymbol{\Delta }}}\propto (1,1,1)={{\rm{isotropic}}}\,{{\rm{s}}},$$

5 $$\cdot \lambda={\lambda }_{0}-a-\sqrt{3}b:{\rm\Delta }\propto (1,{\omega }^{2},\omega )={\mathrm{chiral}}\,{{{\rm{d}}}}^{-},$$

6 $$\cdot \lambda={\lambda }_{0}-a-\sqrt{3}b:{\rm\Delta }\propto (1,{\omega }^{2},\omega )={\mathrm{chiral}}\,{d}^{-},$$

Thus, we find the following results: (i) isotropic *s*-wave SC gap is realized when *η* = 0 because *b* = 0 and *a* > 0. (ii) A chiral *d*^+^ or *d*^−^ SC state is realized for $| b| > \sqrt{3}a$, depending on the sign of *b*. (iii) The sign of *b* depends on *n* even for a fixed **η**. (iv) The ratio ∣*b*/*a*∣ increases as *T* is lowered; see below.

In the LC state, the LDOS pattern shown in Fig. 2a belongs to the *D*_6*h*_ point group, so the chiral *d*-wave state (*E*_2*g*_) and the *s*-wave state (*A*_1*g*_) belong to the different irreducible representation. In addition, TRS breaking in the LC order lifts the degeneracy between *λ*_*d*_^+^ and *λ*_*d*_^−^.

**Fig. 3:**
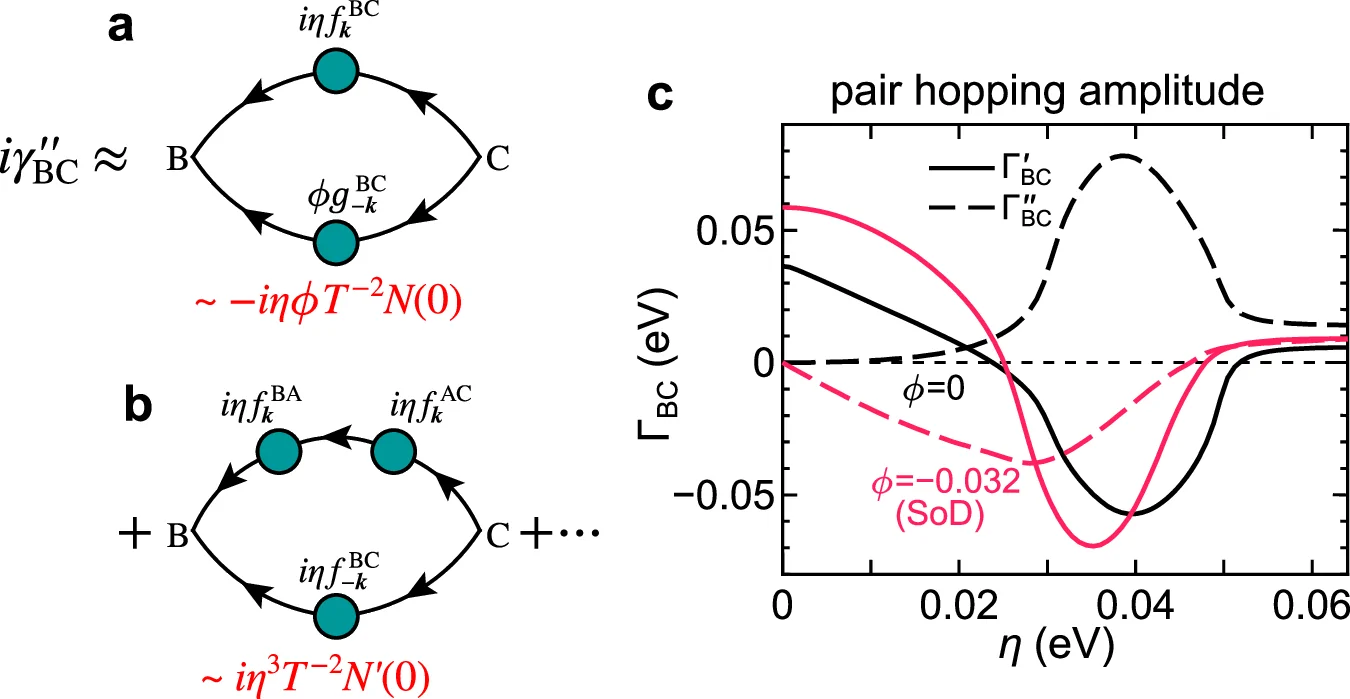
TRS breaking pair-hopping mechanism given by off-diagonal kernel functions. Leading-order expansion terms of $i{\gamma }_{{{\rm{BC}}}}^{{\prime\prime} }$) with respect to *η* and *ϕ*. In the BO phase (*ϕ* ≠ 0), the pair-hopping process **a** gives the leading contribution ${\gamma }_{{{\rm{BC}}}}^{{\prime\prime} }\propto \phi \eta$. For *ϕ* = 0, the process **b** gives the leading contribution ${\gamma }_{{{\rm{BC}}}}^{{\prime\prime} }\propto {\eta }^{3}$. Here, ${\bar{G}}_{k}^{M}$ is the Green function for *η* = *ϕ* = 0, and *N*(0) is the density-of-states. Here, A ≡ *A*_3_, B ≡ *B*_4_, and C ≡ *C*_3_. **c** Numerical results of ${\Gamma }_{{{\rm{BC}}}}^{{\prime} }$ and ${\Gamma }_{{\mathrm{BC}}}^{{\prime\prime} }$ as functions of *η*, at *T* = 0.8 meV. For both *ϕ* = 0 and 0.01, the obtained results for *η* ≲ 0.02 are well explained by the processes (**a**, **b**).

Here, we present an analytic explanation for the emergence of the prominent imaginary pair hopping term $i{\gamma }_{{{\rm{BC}}}}^{{\prime\prime} }$ at low temperatures in the LC phase. In kagome metals, the 12 × 12 green function for *η* = *ϕ* = 0 is approximately sublattice-diagonal around the vHS points: ${\bar {{\rm{G}}}}^{{M}_{\mu }{L}_{\nu }}(k) \approx {Ḡ}^{M}_{\mu \nu }(k) \cdot \delta_{M,L}$^60^. Based on this simplification, the leading-order expansion terms of $i{\gamma }_{{{\rm{BC}}}}^{{\prime\prime} }$ with respect to *η*, *ϕ* are expressed in Fig. 3a, b. (Here, the notations *μ*, *ν* are dropped for simplicity). In the folded BZ, the vHS point is **k** = 0, where the relations *g*^*lm*^ = ± 1 (*g*^*ml*^ = *g*^*lm*^) and *f*^*lm*^ = ± 1 (*f*^*ml*^ = − *f*^*lm*^) are satisfied for the nearest (*m*, *l*) pair.

Figure 3c shows the numerical results for ${\Gamma }_{{{\rm{BC}}}}^{{\prime} }$ and ${\Gamma }_{{\mathrm{BC}}}^{{\prime\prime} }$ as functions of *η* at *T* = 0.8 meV. In the BO phase (*ϕ* ≠ 0), the pair-hopping process illustrated in Fig. 3a provides the leading contribution, $i{\gamma }_{{{\rm{BC}}}}^{{\prime\prime} }\propto \eta \phi {T}^{-2}N(0)$. In the absence of BO (*ϕ* = 0), the leading term instead arises from the process in Fig. 3b, yielding $i{\gamma }_{{{\rm{BC}}}}^{{\prime\prime} }\propto i{\eta }^{3}{T}^{-2}{N}^{{\prime} }(0)$. Consequently, this mechanism generates a phase factor ${\theta }_{{\rm{BC}}}^{\prime} \equiv \arctan ({\Gamma }_{{\rm{BC}}}^{\prime\prime}/{\Gamma }_{{\rm{BC}}}^{\prime})$ in the pair-hopping amplitude, which is particularly enhanced in the BO phase as ${\theta }_{{{\rm{BC}}}}^{{\prime} }\propto \eta \phi$. Importantly, ${\theta }_{{{\rm{BC}}}}^{{\prime} }$ is further amplified at low temperatures, as shown in Fig. 2g.

### Nematic chiral SC states driven by LC + BO order

In kagome metals, the chiral LC order appears inside the star-of-David BO. The nematic LC + BO coexisting state breaks the *C*_6_ symmetry when the chiral LC order and the BO are out-of-phase (i.e., *O*_*ϕ*_ ≠ *O*_*η*_). This *Z*_3_ nematic LC + BO coexistence is energetically favored based on the GL free energy theory for *T* < *T*_LC_ ≪ *T*_BO_^29,30^, and it is observed experimentally^15^. In the following, we analyze the gap equation in the nematic LC + BO coexisting state. Because the LDOS pattern shown belongs to the *D*_2*h*_ point group (see Section “Z3 nematic LC + BO coexisting state” of the Methods), the chiral *d*-wave state (∣*l*_*z*_∣ = 2) belongs to the *A*_1*g*_ representation. Therefore, the *s* + chiral *d*-wave state is expected to be realized, with sizable nematicity and chirality.

**Fig. 4:**
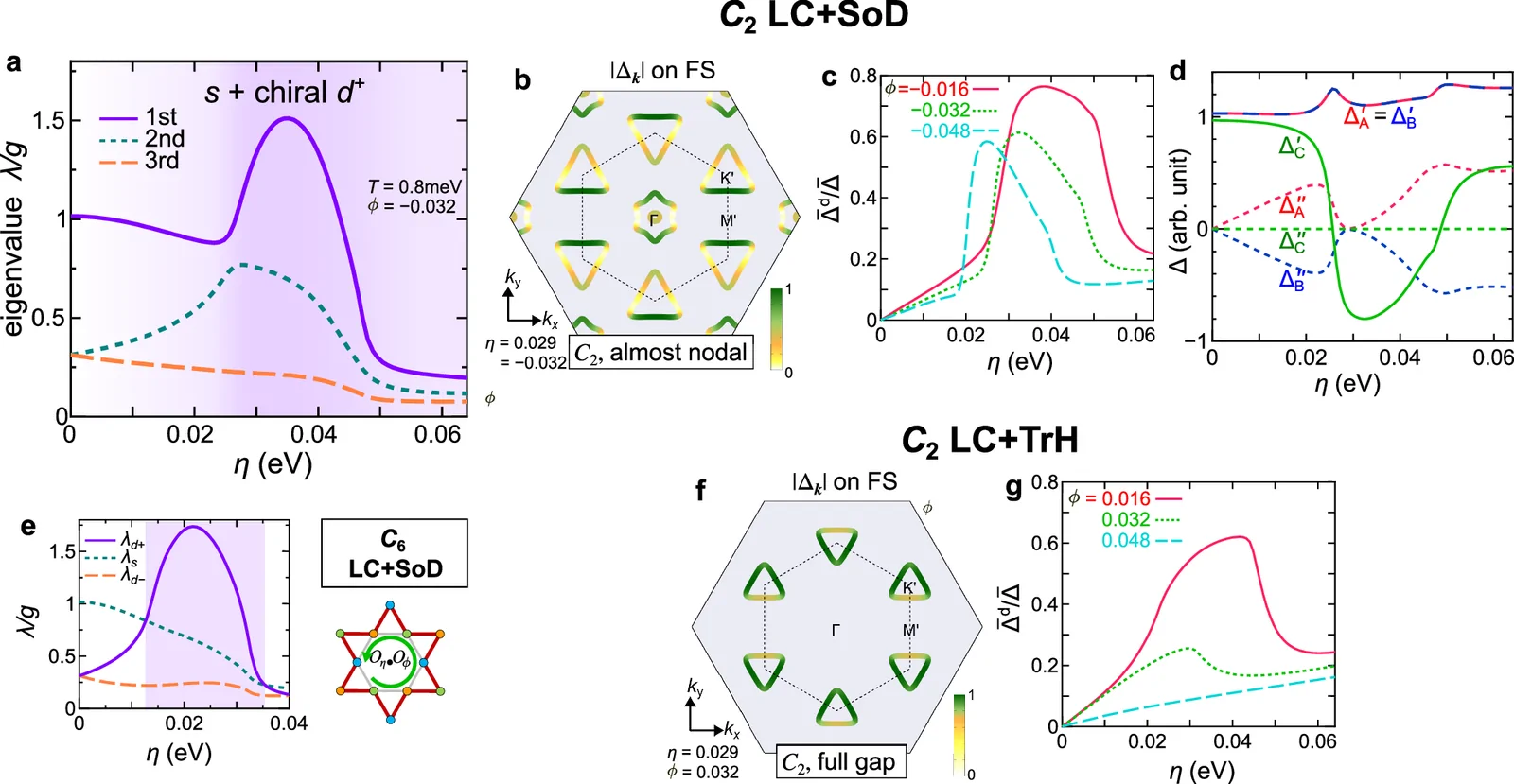
Nematic chiral superconductivity driven by LC + BO phases. **a** The first, second, and third eigenvalues in the *C*_2_ LC + BO(SoD) phase, where *O*_*η*_ and *O*_*ϕ*_ are different. In this case, the *s* + chiral *d*^+^-wave SC state is induced by the LC order. The purple shading represents its *d*^+^-wave component; see the (**c**). **b** Obtained ∣Δ_**k**_∣ for *η* = 0.029 in the SoD phase. The gap structure exhibits pronounced nematic anisotropy along the AB direction (the *k*_*y*_-axis), despite the nearly *C*_6_-symmetric Fermi surface. **c** Obtained *d*-wave SC weight ${\bar{\Delta }}^{d}/\bar{\Delta }$ for *ϕ* = − 0.016 ~ − 0.048 as a function of *η*. The *d*-wave weight exceeds 0.5 for a wide parameter range. **d** Δ_A_, Δ_B_, and Δ_C_ as functions of *η*. **e** Eigenvalues in the *C*_6_ LC + BO phase, where *O*_*η*_ and *O*_*ϕ*_ coincide. In this case, a pure *s*-wave (*d*^+^-wave) state emerges for *η* < *η*_c_ (*η* > *η*_c_), where *η*_c_ = 0.013. **f** Obtained ∣Δ_**k**_∣ for *η* = 0.029 in the *C*_2_ LC + BO(TrH) phase. The gap structure also exhibits sizable nematic anisotropy. **g** Obtained ${\bar{\Delta }}^{d}/\bar{\Delta }$ for *ϕ* = 0.016–0.048 as a function of *η*.

We first discuss the SoD BO state with *ϕ* = − 0.032: Figure 4a shows the first three eigenvalues in the *C*_2_ LC + BO (SoD) phase, in which *O*_*η*_ and *O*_*ϕ*_ are different. At *η* = 0, the largest eigenvalue corresponds to the *s*-wave state, while the second, doubly degenerate eigenvalues correspond to the $({d}_{xy},{d}_{{x}^{2}-{y}^{2}})$-wave states. For *η* ≠ 0, one chiral *d*^+^-wave component mixes into the leading eigenvalue, yielding the TRS-breaking *s* + chiral *d*-wave state. The leading eigenvalue increases sharply around *η* ~ 0.03, indicating an LC-induced enhancement of the condensation energy. Interestingly, the chirality of the *d*-wave component is opposite to that for *ϕ* = 0 shown in Fig. 2f.

As shown in Fig. 4b, the obtained gap magnitude, ∣Δ_**k**_∣, exhibits a highly anisotropic, nematic structure for *η* ≈ ∣*ϕ*∣, due to the LC-induced admixture of *s*-wave and chiral *d*-wave states. The magnitude of the *d*-wave projected component, ${\bar{\Delta }}^{d}/\bar{\Delta }$, is shown in Fig. 4c as functions of *η*. Interestingly, this weight exceeds 0.5 for a wide parameter range. (Note that ${({\bar{\Delta }}^{d})}^{2}+{({\bar{\Delta }}^{s})}^{2}={(\bar{\Delta })}^{2}$). Figure 4d exhibits the sublattice components of the *s* + *d*^±^ wave state for *η*, *ϕ* ≠ 0: We present Δ_A_, Δ_B_, and Δ_C_ as functions of *η*, by setting ImΔ_C_ = 0. Here, A ≡ *A*_3_, B ≡ *B*_4_, and C ≡ *C*_3_.

We briefly discuss the SC state in the *C*_6_ LC + BO (SoD) phase, where *O*_*η*_ and *O*_*ϕ*_ coincide. Unlike in the *C*_2_ LC + BO phase, the *s*-wave and *d*^+^-wave states remain decoupled for *η* ≠ 0. As shown in Fig. 4e, a pure *s*-wave SC state is realized for *η* < *η*_c_ ≡ 0.013, whereas it is switched to a *d*^+^-wave SC state for *η* > *η*_c_. We stress that $| {\lambda }_{{d}^{+}}-{\lambda }_{{d}^{-}}|$ increases linearly with *η*, which is significantly faster than in the case of *ϕ* = 0 shown in Fig. 2f. The present *η*-linear enhancement originates from the *M*_orb_ ∝ *ϕη* term in kagome metals; see Supplementary Note H (Supplementary Information).

Next, we discuss the *C*_2_ LC + BO(TrH) state with *ϕ* = 0.032: Figure 4f shows the obtained anisotropic and nematic gap magnitude, ∣Δ_**k**_∣, for *η* ≈ ∣*ϕ*∣. The weight of the chiral *d*-wave SC is shown in Fig. 4g. Because this Fermi pocket quickly shrinks for *ϕ* > 0, so ${\bar{\Delta }}^{d}/\bar{\Delta }$ becomes small for *ϕ* ≳ 0.03; see the Supplementary Note A (Supplementary Information).

In the LC + BO phase, a prominent chiral SC state emerges due to the finite *ηϕ* term in *θ*_*ml*_, as illustrated in Fig. 3c. This *ηϕ* term is significantly enhanced when an LC-induced small Fermi pocket forms around the *Γ* point, since the process shown in Fig. 3b becomes large owing to the enhanced magnitude of $| \bar{G}{{{\bf{k}}}}^{A}| | \bar{G}{{{\bf{k}}}}^{B}|$ at **k** = ***0***. In the Supplementary Note H (Supplementary Information), we provide a physical explanation of LC-induced chiral superconductivity based on the “OM-chirality coupling mechanism” within the Ginzburg–Landau framework. As we show in the Supplementary Note K (Supplementary Information), the LC-induced chiral SC is further stabilized deep in the SC phase *T* ≪ *T*_c_.

In Section “*n*-dependence of *λx* (*x* = *s*, *d*)” of the Methods, we discuss the eigenvalues (*λ*_*d*_, *λ*_*s*_) for a wide filling range 9.7 ≤ *n *≤ 12.3. The obtained *λ*_*d*_ takes sizable values for *n* ~ 11.8, where the LC-induced small Fermi pocket appears around the *Γ* point. Interestingly, the LC-induced chiral SC state also appears for *n* ~ 10, where one small Fermi pocket appears around the *Γ* point; see Section “LC-induced chiral d-wave state for hole-doped system (*n* = 10.2)” of the Methods.

Experimentally, sizable nematic anisotropy in the SC gap has been observed in refs. ^9–11^, irrespective of the nearly *C*_6_-symmetric Fermi surfaces in the CDW phase. The present nematic chiral SC state will give a natural explanation for the nematic SC gap in kagome metals. However, the isotropic *s*-wave SC state is realized by introducing impurities.

It is worth noting that the present LC + BO phase gives rise to the 2 × 2 PDM as well as nematic SC gap modulation; see Section “2 × 2 pair-density-modulation” of the Methods and the Supplementary Note A (Supplementary Information) for details. The 2 × 2 density wave was analyzed within the Ginzburg–Landau framework in ref. ^61^, whereas here we derive the PDM directly by solving the gap equation for a twelve-site unit-cell model.

### LC + BO induced a nematic chiral SC state in the spin-fluctuation mechanism

In previous sections, we studied the mechanism of the LC-induced chiral SC state in kagome metals. By focusing on the attractive charge-channel pairing interaction^5,6,8,46^, we found that nematic and chiral SC states are stabilized once a moderate LC order (*η* ~ 200 K) is introduced. To further clarify the universality of the LC-induced chiral SC state, it is essential to investigate a wider variety of nonlocal pairing interactions. To this end, we examine spin-fluctuation-mediated superconductivity under the LC phase. Actually, in kagome metals, repulsive spin-channel pairing mechanisms have also been extensively studied.

**Fig. 5:**
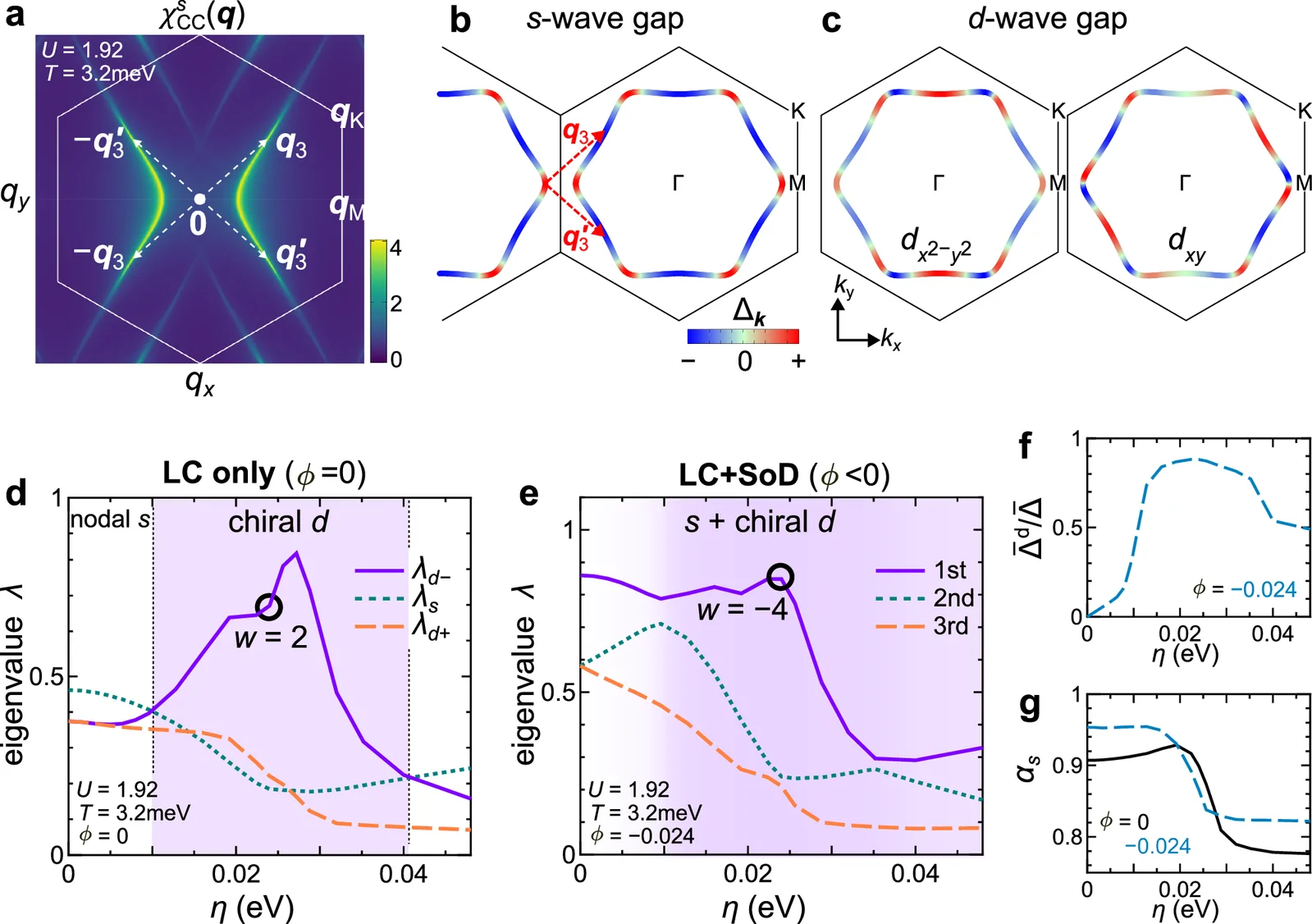
Nematic chiral SC state in the spin-fluctuation mechanism. **a** Spin susceptibility of sublattice C, ${\chi }_{{{\rm{CC}}}}^{s}({{\bf{q}}},0)$, in the original kagome lattice Hubbard model (*η* = *ϕ* = 0) with *U* = 1.92. In the present RPA study, we set $t{\prime}=0.12t$ and *n* = 11.3. Obtained SC gap functions for *η* = *ϕ* = 0: **b** nodal *s*-wave gap function (*λ* = 0.48), and **c** *d*-wave gap function (*λ* = 0.39). **d** The first to third largest eigenvalues as functions of *η* at *ϕ* = 0. The *θ*_**k**_ with *w* = 2 obtained at *η* = 0.02 is shown in the Supplementary Note D (Supplementary Information). **e** Eigenvalues at *ϕ* = − 0.024 (SoD BO). The *θ*_**k**_ with *w* = − 4 obtained at *η* = 0.024 is shown in the Supplementary Note D (Supplementary Information). The purple shading represents the *d*-wave component of the *s* + chiral-*d* wave state. **f** The *d*-wave SC weight ${\bar{\Delta }}^{d}/\bar{\Delta }$ at *ϕ* = − 0.024 (SoD BO) as function of *η*. Here, the weight exceeds 80% over a wide range of *η*. **g** The *η*-dependence of *α*_*S*_ obtained by the RPA.

Here, we perform the random-phase approximation (RPA) analysis, which has been applied to kagome lattice Hubbard models in refs. ^50,62^. The detailed explanations are presented in the Supplementary Note D (Supplementary Information). In Fig. 5a, we show the obtained intra-sublattice spin susceptibility on sublattice C, ${\chi }_{{{\rm{CC}}}}^{s}({{\bf{q}}},0)$, for *U* = 1.92 and *T* = 3.2 meV, where the Stoner factor is *α*_*S*_ = 0.91. (The SDW is realized at *α*_*S*_ = 1). In the present RPA study, we set $t=-0.08,\,{t}^{{\prime} }=0.12t$ and *n* = 11.3. ${\chi }_{{{\rm{CC}}}}^{s}({{\bf{q}}},0)$ exhibits a line-shaped broad peak structure originating from the FS nesting involving the sublattice C. In particular, ${\chi }_{{{\rm{CC}}}}^{s}({{\bf{q}}},0)$ near **q** ≈ ± **q**_3_ and $\pm {{{\bf{q}}}}_{3}^{{\prime} }$ contributes to superconductivity; see the Supplementary Note D (Supplementary Information) for details.

First, we solve the SC gap equation in the absence of the LC order. In this case, spin fluctuations induce a sign-reversing *s*-wave solution as the leading eigenvalue, which is shown in Fig. 5b. Also, a *d*-wave solution emerges as the second-largest eigenvalue, as shown in Fig. 5c. These *s*-wave and *d*-wave solutions are obtained by considering both (i) the optimization of the **k**- and *ϵ*_*n*_-dependent gap function and (ii) off-Fermi-surface contributions. Further details are provided in the Supplementary Note D (Supplementary Information).

Next, we solve the SC gap equation in the pure LC phase based on the twelve-site unit-cell kagome lattice model. Here, we set *T* = 3.2 meV. Figure 5d shows the first to third largest eigenvalues of the SC gap equation as functions of *η* (with *ϕ* = 0). The SC gap functions at *η* = 0 correspond to Fig. 5b, c. Importantly, upon introducing a moderate LC order (*η* ~ 100 K), the chiral SC state with a finite Chern number is realized as the leading instability.

Now, we demonstrate the emergence of the nematic chiral SC states in the LC + BO phase. Figure 5e shows the first to third largest SC eigenvalues at *ϕ* = − 0.024 (SoD BO) as functions of *η*. In this case, the LC-induced *s* + chiral *d*-wave gap function emerges as the leading eigenvalue. The obtained ∣Δ_**k**_∣ and *θ*_**k**_ are explained in the Supplementary Note D (Supplementary Information). Figure 5f exhibits the *d*-wave SC weight ${\bar{\Delta }}^{d}/\bar{\Delta }$ at *ϕ* = − 0.024 (SoD BO) as a function of *η*. This weight exceeds 80% over a wide range of *η*. The *η*-dependence of *α*_*S*_ obtained by the RPA is shown in Fig. 5g.

The obtained nematic chiral SC state is very similar to that obtained by the attraction pairing mechanism shown in Figs. 2 and 4. Therefore, the present LC-driven chiral SC state is robust, independently of the pairing mechanisms. We stress that the nodal *s*-wave state given in Fig. 5b is fragile against impurities. Thus, the charge-channel attractive pairing mechanism can naturally account for the impurity-induced fully gapped *s*-wave state observed in kagome metals^5^.

## Discussions

### Summary and outlook

Rich spontaneous symmetry-breaking phenomena in metals are central to condensed matter physics. In kagome metals *A*V_3_Sb_5_ (*A* = Cs, Rb, K), the normal electronic states exhibit the chiral LC, nematicity, and chirality without in-plane mirror symmetries. The superconducting states in kagome metals also exhibit (i) chirality without TRS, (ii) prominent nematicity, in addition to the (iii) 2 × 2 PDM^63,64^. Unexpectedly, such exotic pairing states change to a (iv) conventional *s*-wave state due to dilute impurities. A comprehensive understanding of this unconventional superconductivity remains elusive to this day.

In this study, we proposed a mechanism of nematic and chiral *d*-wave superconductivity under the coexistence of the LC and BO in the normal state. The LC-induced chiral SC state originates from the “OM-chirality coupling mechanism” owing to the LC-induced finite *M*_orb_; see the Supplementary Note H (Supplementary Information). This mechanism is particularly significant in low-*T*_c_ superconductors, even deep below *T*_c_; see the Supplementary Note K (Supplementary Information). In addition, interesting temperature-induced crossover from ${\bar{\Delta }}^{d}/\bar{\Delta } \sim 0$ to ~0.5 is obtained in the SC state in Supplementary Note K (Supplementary Information). The present LC-induced chiral SC mechanism can emerge for pairing driven by either attractive or repulsive interactions. The attractive interaction may originate from BO fluctuations^8^, in addition to phonons. In contrast, the repulsive interaction may arise from spin fluctuations as well as from the LC-fluctuation mechanism^65^ owing to the odd-parity form factor. (Relatedly, spin-loop-current (sLC) fluctuations can mediate attraction even though they originate in the spin channel, owing to the odd-parity sLC form factor^66^). The pairing strength generated by these fluctuations, which is governed by both proximity to quantum criticality and the magnitude of the coupling constant, remains an important theoretical issue in kagome metals.

When LC order coexists with the BO, nematic chiral SC states due to *s* + chiral *d* state are realized. The obtained ∣Δ_**k**_∣ in Fig. 4 exhibits sizable nematic anisotropy, in spite of the fact that the Fermi surfaces possess almost perfect *C*_6_ symmetry. Therefore, the above-mentioned (i–iii) are naturally explained. In the present mechanism, such unconventional pairing state is easily changed to conventional *s*-wave SC by impurities, consistently with (iv) mentioned above.

In this paper, we analyzed the 2D *d*_*xz*_-orbital kagome lattice model. However, as we explain in the Supplementary Note F (Supplementary Information), real kagome metals possess the p-type Fermi surface made of the *d*_*xz*_-orbital and the m-type Fermi surface made of the *d*_*yz*_-orbitals. The sizes of p-type and m-type Fermi surfaces are comparable, while the bandwidth of the p-type conduction band is narrower. Moreover, the m-type Fermi surface has three-dimensional character, which weakens the singularity of the m-type vHS. Importantly, major portions of the contours are gapped out for $E \sim {E}_{{{\rm{vHS}}}}^{xz}$ due to the inter-orbital hybridization. To verify the robustness of the LC-induced chiral superconductivity, in the Supplementary Note F (Supplementary Information), we perform the SC gap equation analysis based of the 3D (*d*_*xz*_, *d*_*yz*_-orbital) kagome lattice model. The obtained LC-induced chiral state and its wide parameter range are comparable with the numerical results in the main text, strongly indicates the robustness of the LC-induced chiral superconductivity mechanism.

### Intuitive picture of the nematic chiral SC state

**Fig. 6:**
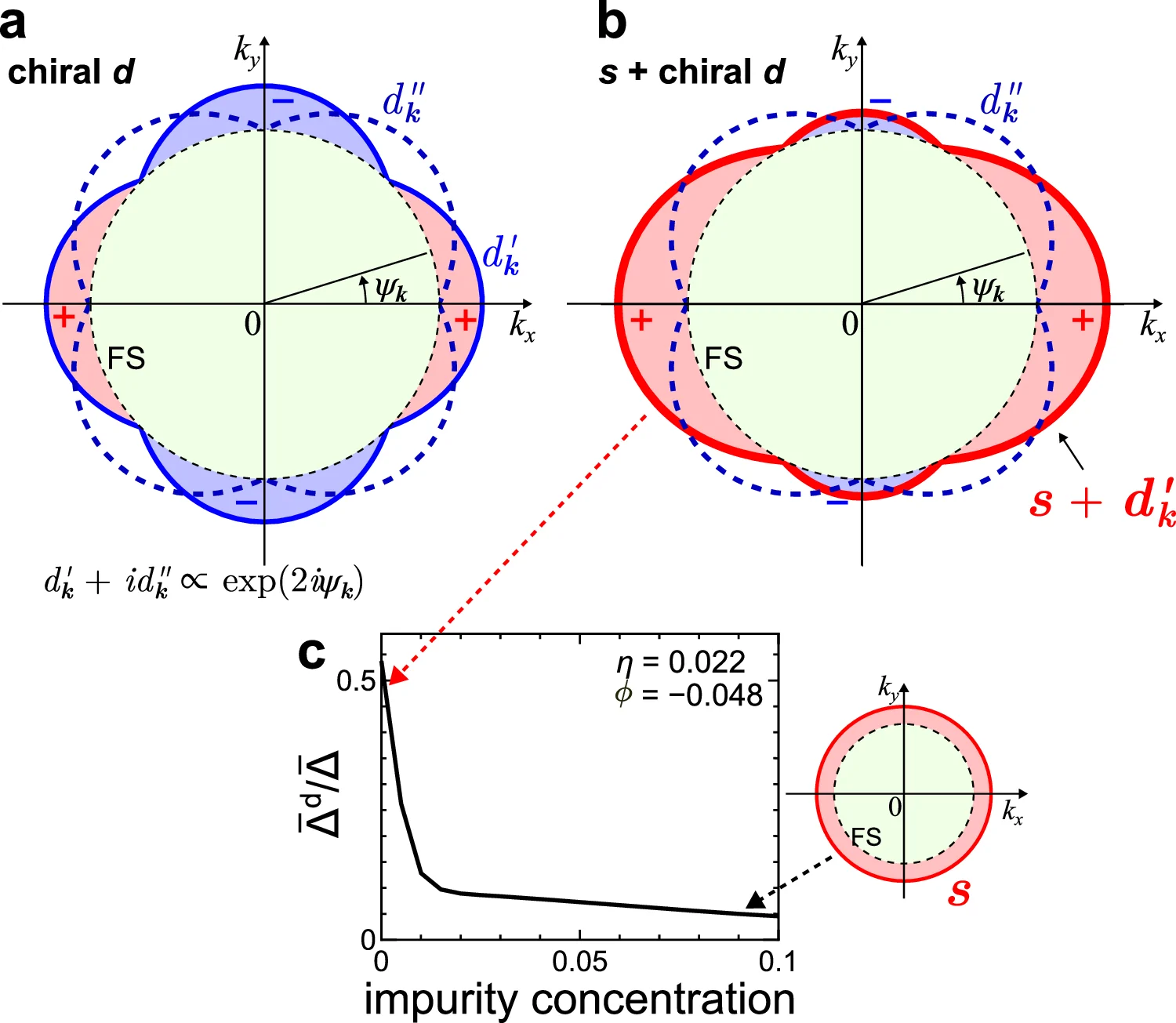
Nematic chiral SC state realized by chiral *d* + *s* state. **a** Chiral *d*-wave gap (${d}_{{{\bf{k}}}}={d}_{{{\bf{k}}}}^{{\prime} }+i{d}_{{{\bf{k}}}}^{{\prime\prime} }$) on a circular Fermi surface. Here, ${d}_{{{\bf{k}}}}={d}_{0}\exp (2i{\psi }_{{{\bf{k}}}}+i{\psi }_{0})$ with ${\psi }_{{{\bf{k}}}}=\arctan ({k}_{y}/{k}_{x})$ for *ψ*_0_ = 0. **b** *s* + chiral *d*-wave gap, given by introducing the isotropic *s*-wave gap (*s *= real) into the chiral *d*-wave gap. The constant phase *ψ*_0_ is fixed to 0, 2*π*/3, or 4*π*/3 under *Z*_3_ LC + BO nematic state^30^. Note that Δ_**k**_ is chiral if ∣*d*_0_∣ > ∣*s*∣. **c** Impurity-induced crossover from chiral *d*-wave type to *s*-wave type gap: ${\bar{\Delta }}^{d}/\bar{\Delta }$ in the LC + BO phase.

Here, we present an intuitive explanation for the nematic chiral SC state due to the *s* + chiral *d* SC state obtained by the present study. Figure 6a represents the chiral *d*-wave gap (${d}_{{{\bf{k}}}}={d}_{{{\bf{k}}}}^{{\prime} }+i{d}_{{{\bf{k}}}}^{{\prime\prime} }$). The dashed circle represents the *E* = 0 level, and ${\psi }_{{{\bf{k}}}}=\arctan ({k}_{y}/{k}_{x})$. Here, ${d}_{{{\bf{k}}}}^{{\prime} }={d}_{0}\cos (2{\psi }_{{{\bf{k}}}}+{\psi }_{0})$ and ${d}_{{{\bf{k}}}}^{{\prime\prime} }={d}_{0}\sin (2{\psi }_{{{\bf{k}}}}+{\psi }_{0})$, where *d*_0_ is a real constant, and the constant phase *ψ*_0_ is fixed to 0, 2*π*/3, or 4*π*/3 under *Z*_3_ LC + BO nematic state^30^. Figure 6b illustrates the *s* + chiral *d* SC state (Δ_**k**_ = *d*_**k**_ + *s*), which is generally realized in the nematic LC + BO phase. Its gap magnitude on the Fermi surface is finite. Its director rotates with *ψ*_0_. Notably, the SC gap Δ_**k**_ has finite chirality (*w* ≠ 0) if ∣*d*_0_∣ > ∣*s*∣. (Note that ∣*d*_0_∣/(∣*s*∣ + ∣*d*_0_∣) corresponds to ${\bar{\Delta }}^{d}/\bar{\Delta }$ shown in Figs. 4 and 5). The nematic SC state found in this study provides a natural explanation for the experimentally observed in-plane anisotropy of *H*_*c*2_ in refs. ^9,10^; see the Supplementary Note I (Supplementary Information) for details.

Figure 6c represents the impurity-induced crossover from chiral *d*-wave type to *s*-wave type gap in the LC + BO phase, as clearly expressed by ${\bar{\Delta }}^{d}/\bar{\Delta }$ as a function of the V-site impurity concentration *n*_imp_; see the Supplementary Note J (Supplementary Information) for details. The obtained impurity-induced *s*-wave state naturally explains the recent real-space STM measurement in ref. ^63,64^. As discussed in the Supplementary Note J (Supplementary Information), *T*_c_ is insensitive to the impurity concentration in the impurity-rich *s*-wave gap region, in contrast to sign-reversing SC states^67–69^. Notably, the temperature also induces pronounced crossover from ${\bar{\Delta }}^{d}/\bar{\Delta } \sim 0$ to ~0.5 in the SC state (*T* < *T*_c_), as we discuss in the Supplementary Note K (Supplementary Information). This result may account for the crossover-like rapid change in the SC gap^70^ and the thermal Hall effect^43^ for *T* ≲ *T*_c_/3. These are important research issues for the future.

In refs. ^53,54^, the important interplay between the LC phase and chiral SC has been discussed. In the present study, from the perspective of the OM-chirality coupling mechanism, we demonstrate the importance of carrier doping away from the van Hove filling and low temperatures and derive the *s* + chiral *d*-wave state induced by the LC + BO phase, thereby providing an explanation for the key experimental observations.

### Comments on key experimental studies

The present study offers crucial insights into the experimentally observed unconventional SC state in kagome metals, including the existence of phase degrees of freedom, TRS breaking, and strong coupling to the LC order. For example, the zero-field SC diode effect and its relation to the LC chirality^41^, switchable Little-Parks oscillation^71,72^, switchable chiral 2 × 2 pair density wave^63,64^, and zero-field Josephson diode effect^73,74^. Notably, recent magnetic circular dichroism ARPES reports the time-reversal-symmetry breaking in CsV_3_Sb_5_^39,40^.

Especially, the recently observed giant thermal Hall effect in CsV_3_Sb_5_ below *T*_c_^43^ would be driven by the present TRS-breaking chiral SC state. The observed ${\kappa }_{xy}^{\exp }$ is several tens of times larger than that expected from the intrinsic mechanism of the chiral superconductor: ${\kappa }_{xy}^{{{\rm{int}}}}=\frac{w\pi {k}_{{{\rm{B}}}}^{2}T}{6\hslash }$, where *w* is the winding number. Based on this observation, ref. ^43^ attributed the giant ${\kappa }_{xy}^{\exp }$ to an extrinsic skew-scattering effect from dilute impurities^75^. The skew-scattering mechanism also operates in the *s* + *d*^±^ wave SC state, irrespective of the value of *w*. This is a highly important issue for future studies.

Another important research direction involves the (4/3) × (4/3) pair-density-wave state and charge-4e and charge-6e superconductivity^44,45^, which remain intriguing open questions. In this study, we examined exotic SC states under the strong influence of LC + BO by treating *ϕ* and *η* as constants, justified by the hierarchy of energy scales *ϕ*, *η* ≫ Δ. However, the achievement of a unified understanding of BO, LC, and SC, as initiated in refs. ^27,28^, remains the ultimate objective.

## Methods

### Method A: Z_3_ nematic LC + BO coexisting state

**Fig. 7:**
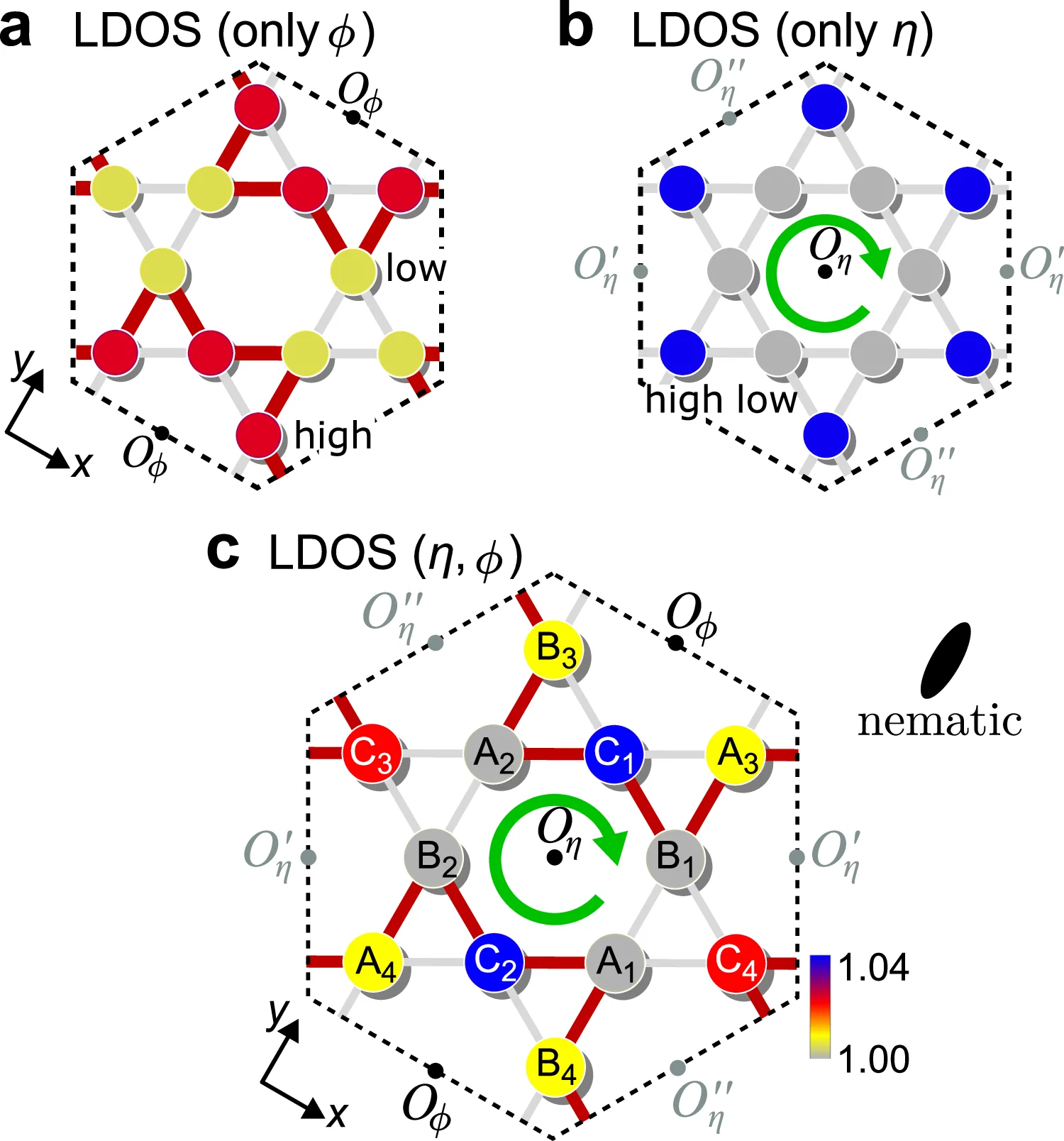
*Z*_3_ nematic LC + BO coexisting state. **a** Schematic LDOS in the BO phase (***ϕ*** ∝ **e**_0_) illustrated in Fig. 1b. The center of *C*_6_ symmetry is shown as *O*_*ϕ*_. **b** Schematic LDOS in the LC phase (*η* ∝ **e**_I_) illustrated in Fig. 1b. The center of *C*_6_ symmetry is shown as *O*_*η*_. **c** LDOS in the *Z*_3_ nematic LC + BO coexisting phase, where the *C*_6_ symmetry center is absent. Here, *η* = − *ϕ* = 0.032. The director is rotated by −2*π*/3 (2*π*/3) by shifting the LC center from *O*_*η*_ to ${O}_{\eta }^{{\prime} }$ (${O}_{\eta }^{{\prime\prime} }$), which corresponds to ${\eta }^{{\prime} }\propto (1,-1,1)$ (*η*^*″*^ ∝ (1, 1, −1)).

In this paper, we analyzed the SC gap equation based on the kagome lattice model with 2 × 2 density waves. A 2 × 2 density wave is described by the combination of three density waves with the wavevector **q** = **p**_1_, **p**_2_, **p**_3_, called the triple-**q** order. Here, we express the set of triple-**q** BO orders as **ϕ** ≡ (*ϕ*_1_, *ϕ*_2_, *ϕ*_3_), where *ϕ*_*n*_ is the order parameter with **q** = **p**_*n*_. Then, *ϕ*_1_, *ϕ*_2_, and *ϕ*_3_ give the BO modulation between the nearest AB-, BC- and CA-bonds, respectively. Figure 1b represents the BO for **ϕ** = *ϕ***e**_0_ with **e**_0_ ≡ (1, 1, 1), which corresponds to *ϕ*_1_ = *ϕ*_2_ = *ϕ*_3_. Then, the center of *C*_6_ symmetry is *O*_*ϕ*_. The BO-induced 2 × 2 modulation of the local density-of-states (LDOS) is schematically shown in Fig. 7a.

In the same way, the 2 × 2 LC is given by the triple-**q** order parameters *η* ≡ (*η*_1_, *η*_2_, *η*_3_). *η*_1_, *η*_2_, and *η*_3_ give the LC orders that induce the charge current along AB-, BC- and CA-bonds, respectively. Then, the chirality of the triple-**q** LC state is described as ${\chi }_{{{\rm{LC}}}}\equiv {{\rm{sgn}}}\{{\eta }_{1}{\eta }_{2}{\eta }_{3}\}$. The uniform OM *M*_orb_ is proportional to *χ*_LC_. Figure 1b represents the LC for *η* = *η***e**_I_ with **e**_I_ ≡ (1, 1, 1), where the center of *C*_6_ symmetry is *O*_*η*_. The LC-induced 2 × 2 modulation of the LDOS is schematically shown in Fig. 7b. As explained in refs. ^29,30^, the center of *C*_6_ symmetry moves to ${O}_{\eta }^{{\prime} }$ (${O}_{\eta }^{{\prime\prime} }$) for ${\eta }^{{\prime} }=\eta {{{\bf{e}}}}_{{\mathrm{II}}}$ (*η*^*″*^ = *η***e**_III_), without changing *χ*_LC_. Here, **e**_II_ ≡ (−1, −1, 1) and **e**_III_ ≡ (−1, 1, −1). See the Supplementary Note A (Supplementary Information) for details.

As explained in the main text, the LC order parameter attaches the effective Aharonov–Bohm phase to conduction electrons. For this reason, the chiral *d*-wave SC state, (Δ_A_, Δ_B_, Δ_C_) ∝ (*ω*, *ω*^2^, 1) or (*ω*^2^, *ω*, 1), can be realized under the 2 × 2 LC state. Its chirality depends on the LC chirality as well as the electron filling.

Next, we consider the coexistence between the BO and the LC. When *O*_*ϕ*_ and *O*_*η*_ are different, the *C*_6_ symmetry center disappears, leading to the electronic nematic state. Figure 7c represents the obtained nematic LDOS pattern under **ϕ** = *ϕ***e**_0_ and *η* = *η***e**_I_. The director is rotated by −2*π*/3 (+2*π*/3) by replacing *η*_I_ with *η*_II_ (*η*_III_). A Ginzburg–Landau analysis shows that the nematic coexistence is realized when *T*_LC_ ≪ *T*_BO_^29,30^. In this *Z*_3_ nematic state, the chiral *d*-wave SC state and the *s*-wave SC state coexist as a consequence of reduced symmetry. This fact leads to the nematic chiral SC state as revealed in this paper. The nematicity and the chirality in the SC state becomes prominent for *n* ~ 11.84, where the original Fermi surface is close to three vHS points. Under the triple-**q** order, three vHS points form a small Fermi pocket as depicted in Fig. 1c, due to the LC-induced band reconstruction. This reconstructed Fermi pocket gives rise to sizable complex number kernel function *Γ*_*ml*_ (*m* ≠ *l*) shown in Fig. 3, which is the origin of the prominent nematic and chiral SC state in the gap equation.

### Method B: 2 × 2 pair-density-modulation

A recent experimental study^14^ reports the emergence of the 2 × 2 PDM in *A*V_3_Sb_5_ below *T*_c_. The modulation of the period 2*a* SC gap due to the PDM is about 5–10%. Such a large modulation would not be simply explained because the 2 × 2 modulation of the local DOS due to LC + BO order is about 1–2%. Here, we explain that the (nematic) chiral *d*-wave state driven by the LC order exhibits sizable 2 × 2 PDM.

**Fig. 8:**
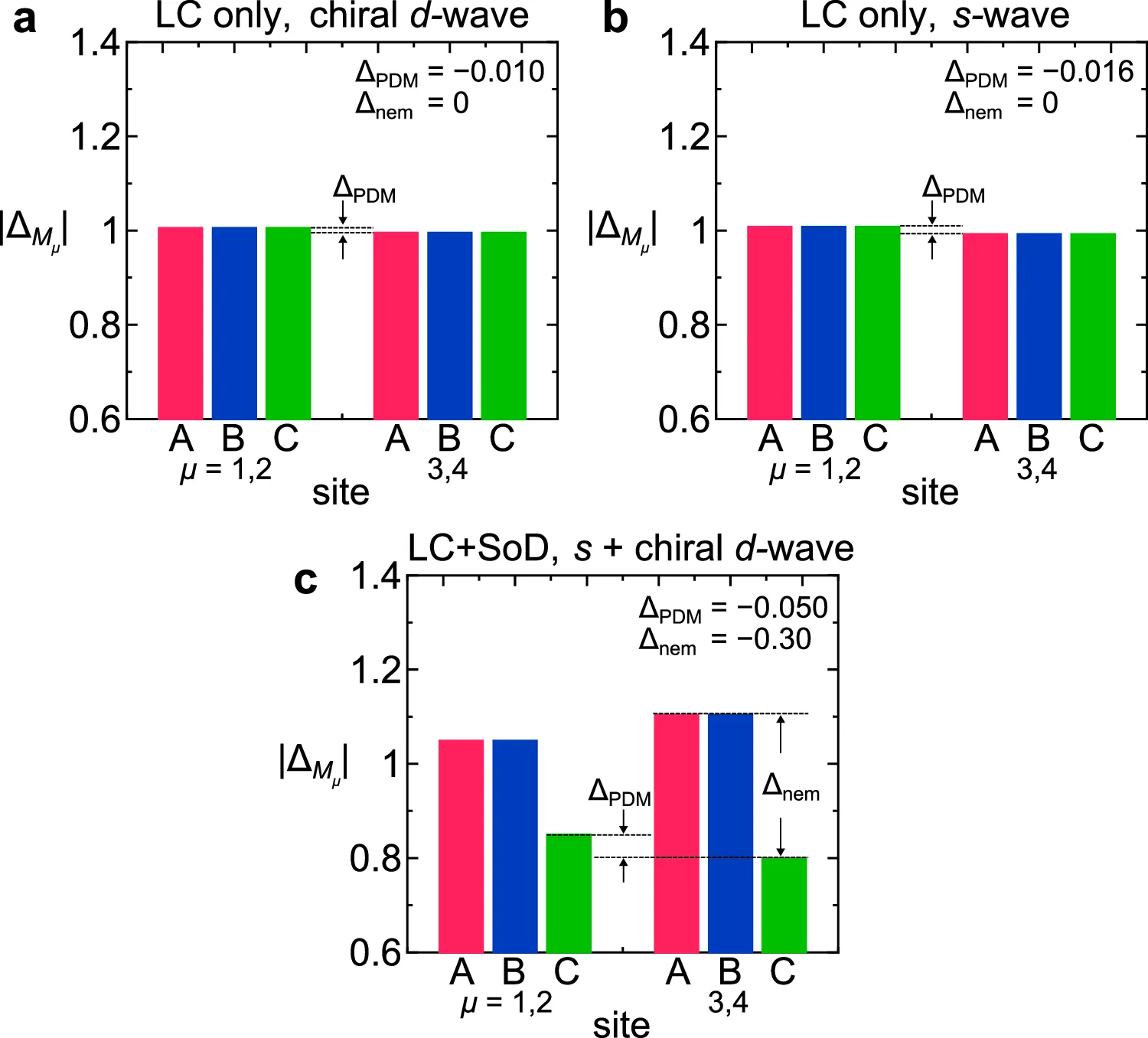
2 × 2 PDM in the LC and LC + BO phases. $| {\Delta }_{{M}_{1}}|$ and $| {\Delta }_{{M}_{3}}|$ (*M *= A,B,C): **a** $| {\Delta }_{{M}_{\mu }}|$ in the chiral *d*-wave state for *η* = 0.032 (*ϕ* = 0) at *T* = 0.8 meV. **b**$| {\Delta }_{{M}_{\mu }}|$ in the *s*-wave state, which is the second largest eigenvalue in the numerical study for (**a**). In **a**, **b**, the 2 × 2 PDM, ${\Delta }_{{{\rm{PDM}}}}\equiv {{\max }_{M}}\{| {\Delta }_{{M}_{3}}| -| {\Delta }_{{M}_{1}}| \}$, is very small. **c**$| {\Delta }_{{M}_{\mu }}|$ in the nematic chiral SC state in the LC + BO (SoD) state with *η* = − *ϕ* = 0.032 at *T* = 0.8 meV. This SC state exhibits large PDM (Δ_PDM_) and large nematicity (${\Delta }_{{{\rm{nem}}}}\equiv {{\max }_{\mu }}\{| {\Delta }_{{{{\rm{A}}}}_{\mu }}| -| {\Delta }_{{{{\rm{C}}}}_{\mu }}| \}$).

First, we discuss the PDM under the pure LC state without BO. In this case, $| {\Delta }_{{M}_{\mu }}|$ is independent of *M *= A, B, and C. Figure 8a exhibits the $| {\Delta }_{{M}_{\mu }}|$ in the chiral *d*-wave state for *η* = 0.032 (*ϕ* = 0) at *T* = 0.8 meV. (Both ∣Δ_**k**_∣ and *θ*_**k**_ in the chiral *d*-wave state for the same parameter are shown in Fig. 2 in the main text). In this case, $| {\Delta }_{{M}_{1}}|=| {\Delta }_{{M}_{2}}|$ deviates from $| {\Delta }_{{M}_{3}}|=| {\Delta }_{{M}_{4}}|$. The second largest eigenvalue corresponds to the *s*-wave state, whose $| {\Delta }_{{M}_{\mu }}|$ is shown in Fig. 8b. In both Fig. 8a, b, finite ${\Delta }_{{{\rm{PDM}}}}\equiv {{\max }_{M}}\{| {\Delta }_{{M}_{3}}| -| {\Delta }_{{M}_{1}}| \}$ means the 2 × 2 PDM, due to the translational symmetry breaking in the SC state.

Next, we discuss the PDM under the *Z*_3_-nematic LC + BO phase discussed in the main text. Figure 8c exhibit the $| {\Delta }_{{M}_{\mu }}|$ in the *s* + chiral *d*-wave state for *η* = − *ϕ* = 0.032. (Both ∣Δ_**k**_∣ and *θ*_**k**_ for the same parameter are shown in Fig. 4 in the main text). Notably, both the PDM Δ_PDM_ and the nematicity expressed by ${\Delta }_{{{\rm{nem}}}}\equiv {{\max }_{\mu }}\{| {\Delta }_{{{{\rm{A}}}}_{\mu }}| -| {\Delta }_{{{{\rm{C}}}}_{\mu }}| \}$ become large in the *s* + chiral *d*-wave state. To summarize, the present *s* + chiral *d*-wave SC state in the LC + BO phase exhibits prominent chirality and nematicity, both of which are widely observed in kagome metals.

### Method C: *n*-dependence of *λ*_*x*_ (*x* = *s*, *d*)

In the main text, we performed the numerical study only for the electron filling *n* = 11.84. Here, we verify the chiral *d*-wave SC state is realized for a finite range of *n*.

**Fig. 9:**
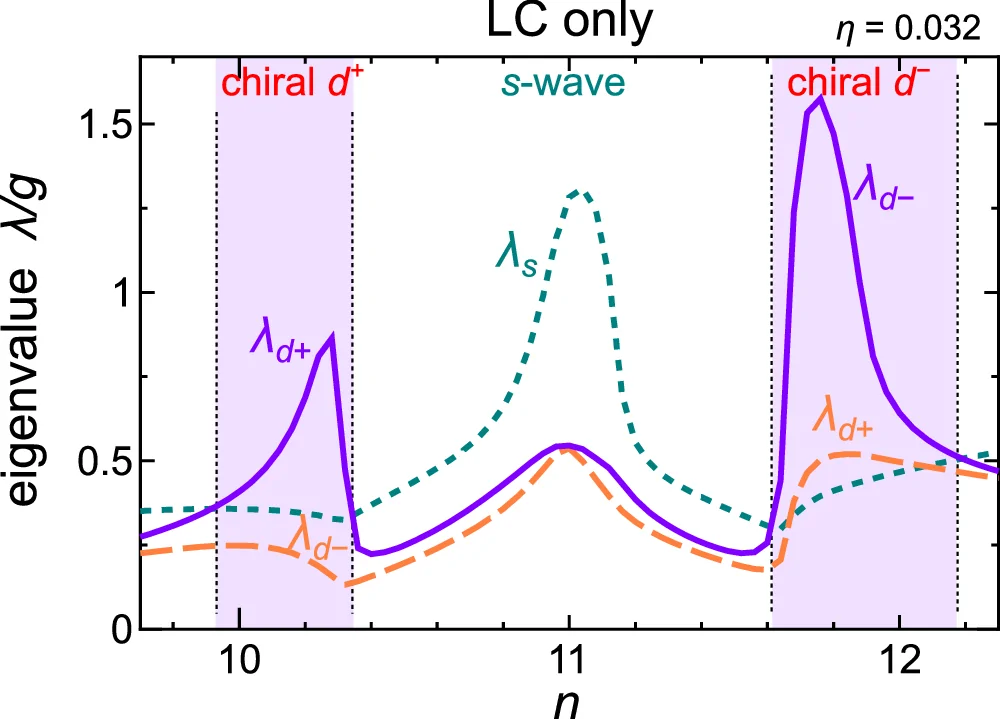
*n*-dependence of *λ*_*x*_ (*x* = *s*, *d*). The largest and second-largest eigenvalues as functions of *n* = 9.7–12.3, where (*η*, *ϕ*) = (0.032, 0) at *T* = 0.8 meV. The chiral *d*-wave state dominates over the *s*-wave state in the region 9.9 ≲ *n* ≲ 10.4 and 11.6 ≲ *n* ≲ 12.2, where a single small Fermi pocket appears around the *Γ* point. The region of the chiral *d*-wave state is enlarges for larger *η*. For CsV_3_Sb_5_, the filling is *n* ≈ 11.84; see the Supplementary Note E (Supplementary Information). The van-Hove filling without LC and BO is *n*_vHS_ ≈ 11.

Figure 9 represents the first to third SC eigenvalues as functions of *n*, where (*η*, *ϕ*) = (0.032, 0) at *T* = 0.8 meV. The chiral *d*-wave state dominates over the *s*-wave state in the region 11.6 ≲ *n* ≲ 12.2 and 9.9 ≲ 10.4, where single small Fermi pockets appear around the *Γ* point. As we discuss in the main text, the chiral *d*-wave SC state emerges when three vHSs composed of all sublattices (A, B, C) form a small hybridized Fermi surface around the *Γ* point under the triple-**q** LC state. Note that *λ*_*s*_ is drastically enlarged near the vHS filling *n* ≈ 11.

### Method D: LC-induced chiral *d*-wave state for hole-doped system (*n* = 10.2)

**Fig. 10:**
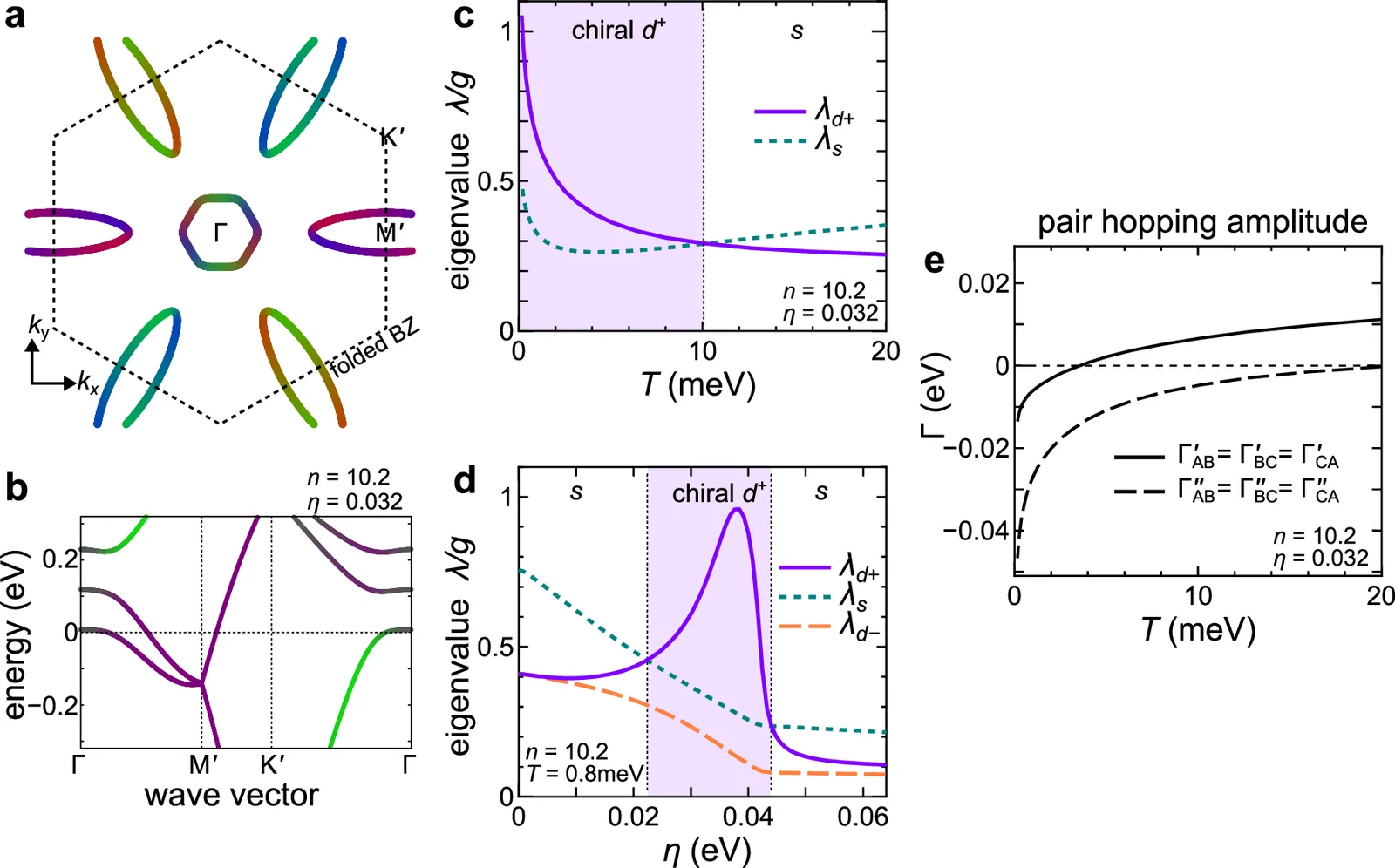
LC-induced chiral *d*-wave state at *n* = 10.2. **a** Fermi surface and **b** band structure for *η* = 0.032 (*ϕ* = 0) at *n* = 10.2. **c** The largest and second-largest eigenvalues as functions of *T*. The chiral *d*-wave state with the transition temperature *T*_c_ ≲ 2 meV is realized for *g* ≳ 2. **d** The three largest eigenvalues as functions of *η* at *T* = 0.8 meV. At *η* = 0, the relation ${\lambda }_{s} > {\lambda }_{{d}^{+}}={\lambda }_{{d}^{-}}$ is satisfied. ${\lambda }_{{d}^{+}}$ gradually increases for finite *η*, and chiral *d*^+^ wave state is realized for *η* > 0.02. **e** Complex off-diagonal kernel functions *Γ*_AB_, *Γ*_BC_, and *Γ*_CA_ as functions of *T*. Here, A ≡ *A*_3_, B ≡ *B*_4_, and C ≡ *C*_3_.

In the present paper, we studied the chiral *d*-wave state under the LC phase in kagome metals, by focusing the electron filling *n* ~ 11.8. To verify the robustness of the present analysis, we perform the numerical study for *n* = 10.2. Figure 10a represents the Fermi surface and **b** band-structure for *n* = 10.2 and *η* = 0.032 (*ϕ* = 0), where hole-like Fermi pockets appear. Figure 10c shows the obtained largest and second-largest eigenvalues as functions of *T*. The chiral *d*-wave state with the transition temperature *T*_c_ ≲ 2meV is realized for *g* ≳ 2. Figure 10d represents the three largest eigenvalues as functions of *η* at *T* = 0.8 meV. At *η* = 0, the relation ${\lambda }_{s} > {\lambda }_{{d}^{+}}={\lambda }_{{d}^{-}}$ is satisfied. ${\lambda }_{{d}^{+}}$ gradually increases for finite *η*, and chiral *d*^+^ wave state is realized for *η* > 0.02. The complex off-diagonal kernel functions *Γ*_AB_, *Γ*_BC_, and *Γ*_CA_, which are the driving force of the chiral SC state, are shown in Fig. 10e. Here, A ≡ *A*_3_, B ≡ *B*_4_, and C ≡ *C*_3_. Thus, the LC-driven chiral *d*-wave SC state is expected to be realized for both *n* ~ 10.2 and *n* ~ 11.8.

### Reporting summary

Further information on research design is available in the Nature Portfolio Reporting Summary linked to this article.

## Supplementary information

Supplementary Information

Reporting Summary

Transparent Peer Review File

## Data Availability

The numerical source data underlying main-text Figs. 1–10 and S1–S12 have been deposited in the figshare repository at 10.6084/m9.figshare.29291783^76^.

## Code availability

The code used to perform the numerical calculations for the superconducting gap equation analysis of kagome-lattice tight-binding models is available from the corresponding author upon request. The code is not publicly released at this time as it is part of ongoing research and has not yet been finalized for broader distribution.
