# Supplementary material for: Nematic and chiral superconductivity emerging within the loop-current phase in kagome metals
Source: Nat Commun. 2026 Jul 27;17:9114. doi: 10.1038/s41467-026-75259-3 (PMC13518961; doi:10.1038/s41467-026-75259-3)
Supplement: Supplementary file 1 [file 41467_2026_75259_MOESM1_ESM.pdf]

[Supplementary Information]

Nematic and chiral superconductivity emerging within the loop-current phase in kagome metals

Rina Tazai[1], Youichi Yamakawa[2], and Hiroshi Kontani[2]

[1] *Center for Emergent Matter Science, RIKEN, Wako, Saitama 351-0198, Japan.*

[2] *Department of Physics, Nagoya University, Nagoya 464-8602, Japan.*

Supplementary Note A: Twelve-site unit cell model with BO and LC order parameters

The bond/current order is the modulation of the hopping integral between i and j atoms due to the electron correlation, $\delta t_{ij}^{\mathrm{b/c}}$. Theoretically, it is the symmetry breaking in the self-energy, and it is derived from the density-wave (DW) equation [1, 2]. The wavevectors of the bond and current orders correspond to the inter-sublattice nesting vectors $\mathbf{q} = \mathbf{p}_n$ ($n = 1-3$). The triple-Q ($3Q$) bond order between the nearest V atoms is given as

$$\delta t_{ij}^{\mathrm{b}} = \phi_1 g_{ij}^{\mathrm{AB}} + \phi_2 g_{ij}^{\mathrm{BC}} + \phi_3 g_{ij}^{\mathrm{CA}}, \tag{1}$$

where ϕ_n ($n = 1-3$) is the BO parameter with the wavevector $\mathbf{p}_n$. ϕ_1, ϕ_2, and ϕ_3 correspond to the BO along the AB-axis, BC-axis, and CA-axis, respectively. $g_{ij}^{\alpha\beta} = g_{ji}^{\alpha\beta}$ is the even-parity BO form factor along $\alpha\beta$-axis ($\alpha\beta$ =AB, BC, CA). Here, we assume that $g_{ij}^{\alpha\beta}$ is finite only for the nearest-neighbor sites (i, j) on the $\alpha\beta$-axis.

In the main text, we denote the twelve sublattices as A_μ, B_μ, and C_μ ($\mu = 1-4$). Here, we introduce an alternative labeling scheme, $(1, 2, \cdots, 12) \equiv (A_4, B_2, C_2,\ A_2, B_3, C_1,\ A_1, B_1, C_4,\ A_3, B_4, C_3)$, following Refs. [1, 2].

Here, we introduce the set of BO parameter $\boldsymbol{\phi} \equiv (\phi_1, \phi_2, \phi_3)$. We also define the BO form factor $g_{ij}^{\mathrm{AB}} = +1$ $[-1]$ when the nearest-neighbor pair (i, j) belongs to the sublattice pair $(1, 2), (4, 5), (8, 10), (11, 7)$ $[(2, 4), (5, 1), (7, 8), (10, 11)]$. In the same manner, other form factors associated with $\mathbf{q} = \mathbf{p}_2$ and $\mathbf{p}_3$, namely g_{ij}^{BC} and g_{ij}^{CA}, are also introduced. Then, the $3Q$ bond order shown in Supplementary Figure 1 **a** is expressed as $\boldsymbol{\phi} = \phi\mathbf{e}_0$, where $\mathbf{e}_0 = (1, 1, 1)/\sqrt{3}$. Then, the TrH (SoD) bond-order is realized for $\phi > 0$ ($\phi < 0$), as shown in Supplementary Figure 1 **a**.

Next, we introduce the $3Q$ current order between the nearest V atoms:

$$\delta t_{ij}^{\mathrm{c}} = \eta_1 f_{ij}^{\mathrm{AB}} + \eta_2 f_{ij}^{\mathrm{BC}} + \eta_3 f_{ij}^{\mathrm{CA}}, \tag{2}$$

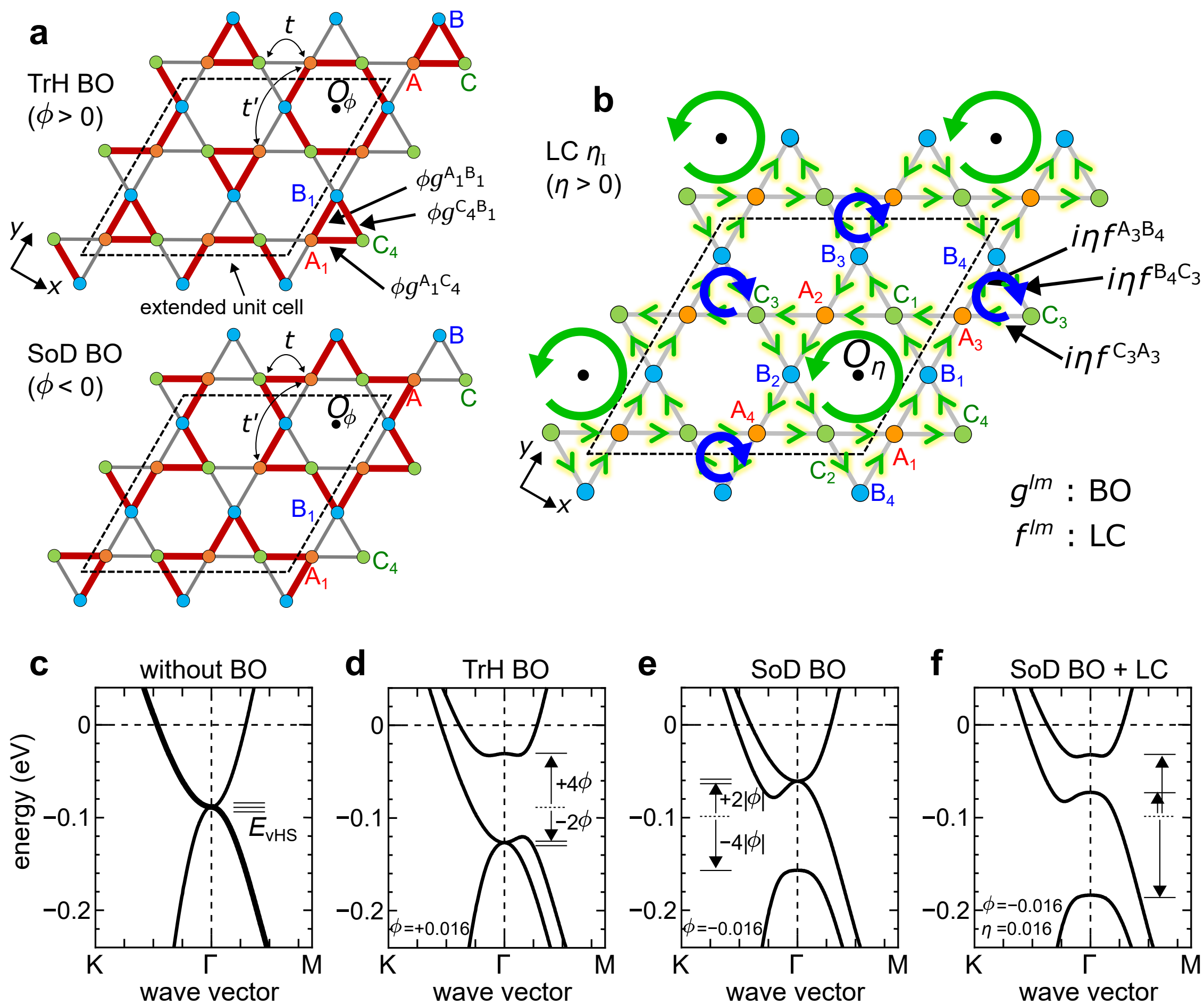

Supplementary Figure 1. **BO and LC order in twelve-site unit-cell kagome lattice model**. **a** BO parameter $\boldsymbol{\phi}_0$ in real space for TrH pattern ($\phi > 0$) and SoD pattern ($\phi < 0$). **b** LC parameter $\boldsymbol{\eta}_\alpha$ (α=I) in real space ($\eta > 0$). **c** Band structure without LC and BO in the folded BZ. **d** Band structure in the TrH BO. **e** Band structure in the SoD BO. **f** Band structure in the SoD BO + LC.

where η_n is the LC parameter with the wavevector $\mathbf{p}_n$. η_1, η_2, and η_3 correspond to the BO along the AB-axis, BC-axis, and CA-axis, respectively. $f_{ij}^{\alpha\beta} = -f_{ji}^{\alpha\beta}$ is the odd-parity LC form factor along $\alpha\beta$-axis ($\alpha\beta$ =AB, BC, CA).

Here, we introduce the set of LC parameter $\boldsymbol{\eta} \equiv (\eta_1, \eta_2, \eta_3)$. We also define the LC form factor $f_{ij}^{\rm AB} = +i$ $[-i]$ when the nearest-neighbor pair (i, j) belongs to the sublattice pair $(l, m) = (1, 2), (2, 4), (4, 5), (5, 1)$ $[(7, 8), (8, 10), (10, 11), (11, 7)]$. Similarly, the form factors $f_{ij}^{\rm BC}$ and $f_{ij}^{\rm CA}$ associated with $\mathbf{q} = \mathbf{p}_2$ and $\mathbf{p}_3$ are defined as illustrated in Supplementary Figure 1 **b**. Then, the $3Q$ current order shown in Supplementary Figure 1 **b** is expressed as $\boldsymbol{\eta} = \eta \mathbf{e}_\alpha$, where $\mathbf{e}_{\rm I} = (a, 1, 1)\sqrt{3/(2 + a^2)}$, $\mathbf{e}_{\rm II} = (-1, -a, 1)\sqrt{3/(2 + a^2)}$, and $\mathbf{e}_{\rm III} = (-1, 1, -a)\sqrt{3/(2 + a^2)}$.

When $|\eta/\phi| \ll 1$, the LC + BO coexisting state is given by ($\boldsymbol{\eta}_\alpha$, $\boldsymbol{\phi}_0$) with $a \lesssim 2$ according to the GL free-energy analysis [2, 3]. (Note that $2 > a > 1$: $a \approx 2$ just below $T_{\rm LC}$, and decreases upon lowering T. For simplicity, we set $a = 1$ in this paper, although similar results are obtained for $a = 2$.) Then, the electronic states become nematic, with the director aligned along the ab direction for α=I, the bc direction for α=II, and the ca direction for α=III.

In the theoretical model of the main text, the tight-binding hopping integral is given as $t_{ij} = t^0_{ij} + \delta t^{\rm b}_{ij} + \delta t^{\rm c}_{ij}$, where $t^0_{i,j}$ is the original hopping integral with D_{6h} symmetry. Then, the tight-binding Hamiltonian in the momentum representation is

$$\hat{H} = \sum_{l,m}^{1-12} \sum_{\mathbf{k},\sigma} t^{l,m}_{\mathbf{k}} c^\dagger_{\mathbf{k},l,\sigma} c_{\mathbf{k},m,\sigma}, \tag{3}$$

where $\mathbf{k}$ is the wavevector and $l(m) = 1 - 12$ represent sublattice in the unit-cell, and $t^{l,m}_{\mathbf{k}}$ is the Fourier transform of the hopping integral $t_{i,j}$.

Here, we introduce the following 12×12 Green functions:

$$\hat{G}(k) = ((i\epsilon_n + \mu)\hat{1} - \hat{t}_{\mathbf{k}})^{-1}, \tag{4}$$

where $k \equiv (\mathbf{k}, \epsilon_n)$ and $\epsilon_n \equiv (n+1)\pi T$. We also introduce the Green function for $\eta = \phi = 0$ as $\hat{\bar{G}}(k)$. At the vHS point, $\bar{G}_{lm}(k) = 0$ when l and m belong to the different sublattices A−C, by reflecting the sublattice interference of kagome lattice model. In contrast, $G_{lm}(k)$ has finite off-diagonal elements when $\eta, \phi \neq 0$.

Supplementary Figures 1 **c**-**f** represent the band dispersion around the Γ point in the cases of **c** without BO and LC, **d** the TrH BO state, **e** the SoD BO state, and **f** the nematic SoD BO + LC state. The parameters ϕ and η are written in these figures. In the BO state, the threefold vHS states at the Γ point split into $E_{\rm vHS} + 4\phi$ (nondegenerate) and $E_{\rm vHS} - 2\phi$ (doubly degenerate). This doubly degeneracy is lifted by introducing the LC order. Thus, in the LC+BO state, the band hybridization causes a small electron-pocket, which plays an important role in the chiral d-wave state with finite η.

In our definition of the SoD BO ($\phi < 0$), the SoD pattern is obtained by connecting bonds (i, j) with $\delta t_{ij} > 0$ (the TrH pattern is obtained for $\phi > 0$.) Then, the BO order induces changes in the V–V bond lengths, δa_{ij}, via finite electron–lattice interactions. However, the constant $L \equiv \delta a_{ij}/\delta t_{ij}$ depends sensitively on the microscopic details of the electron–lattice coupling; therefore, the sign and magnitude of L have been unsolved. Experimentally, various values of δa_{ij} have been reported

for A =Cs, Rb, K, and no clear consensus has yet been reached. In particular, for A = Cs, a 2×2×4 charge order in which SoD and TrH layers coexist has been proposed in several experiments.

As for the origin of the bond order, both an electron-correlation-driven mechanism and an electron–lattice coupling mechanism have been proposed, and it is likely that the BO emerges from the cooperation of these two effects. (We note that lattice relaxations obtained from first-principles calculations tend to overestimate the lattice deformation.) Regarding the origin of the LC order, both the BO-fluctuation mechanism and the second-nearest-neighbor Coulomb repulsion mechanism have been discussed, and these two mechanisms may also cooperate in real materials.

Supplementary Note B: Kernel function $\hat{\Gamma}$ in the SC gap equation

In the main text, we present the some elements of the kernel functions Γ_{lm} obtained by the numerical study in Fig. 10 **e** and Fig. 3 **d**. It is revealed that the sizable imaginary part Γ''_{lm} in the LC and LC+BO phases give rise to the chiral SC state.

Here, we present all the elements of the kernel functions Γ_{lm} Supplementary Figure 2 shows the obtained Γ_{M_μ,L_ν} for M_μ =A_3, B_4, and C_3 at $\phi = 0.032$ eV. (Hereafter, we denote A≡ A_3, B≡ B_4, C≡ C_3.) In each panel, twelve lines for all L_ν are expressed. Thus, Γ_{lm} exhibits sizable imaginary part for all $l \neq m$, Interestingly, the obtained Γ_{A,L_ν}, Γ_{B,L_μ}, and Γ_{C,L_μ} are nearly independent of μ for any M =A, B, C. Therefore, it is verified that Γ_{M_μ,L_ν} is approximately independent of μ and ν, as we mentioned in the main text. The relation $\Gamma_{A,L_\nu} = (\Gamma_{B,L_\nu})^*$ is verified. Unless otherwise noted, energy is given in units of eV hereafter.

Supplementary Note C: LC-induced enhancement of the chiral d-wave eigenvalue

In the main text, we demonstrate the mechanism of the LC-induced chiral SC state based on the SC gap equation formalism. We find that the Cooper pair propagator Γ_{lm} possesses extra terms $\gamma_{lm} = \gamma'_{lm} + i\gamma''_{lm}$ when $\eta, \phi \neq 0$. By solving the SC gap equation, it is revealed that the imaginary pair-hopping induced by $i\gamma''_{lm}$ gives rise to the TRS-breaking chiral SC gap.

Here, we investigate why the chiral d-wave eigenvalue is enhanced by $i\gamma''_{lm}$. For simplicity, we assume the triple-**q** LC and BO orders given by $\eta(1,1,1)$ and $\phi(1,1,1)$, respectively. Then, the expansion of $\hat{\gamma} \equiv \hat{\gamma}' + i\hat{\gamma}''$ is given by

$$\gamma'_{lm} = \beta^{(2,0)}_{lm}\eta^2 + \beta^{(0,2)}_{lm}\phi^2 + \cdots , \tag{5}$$

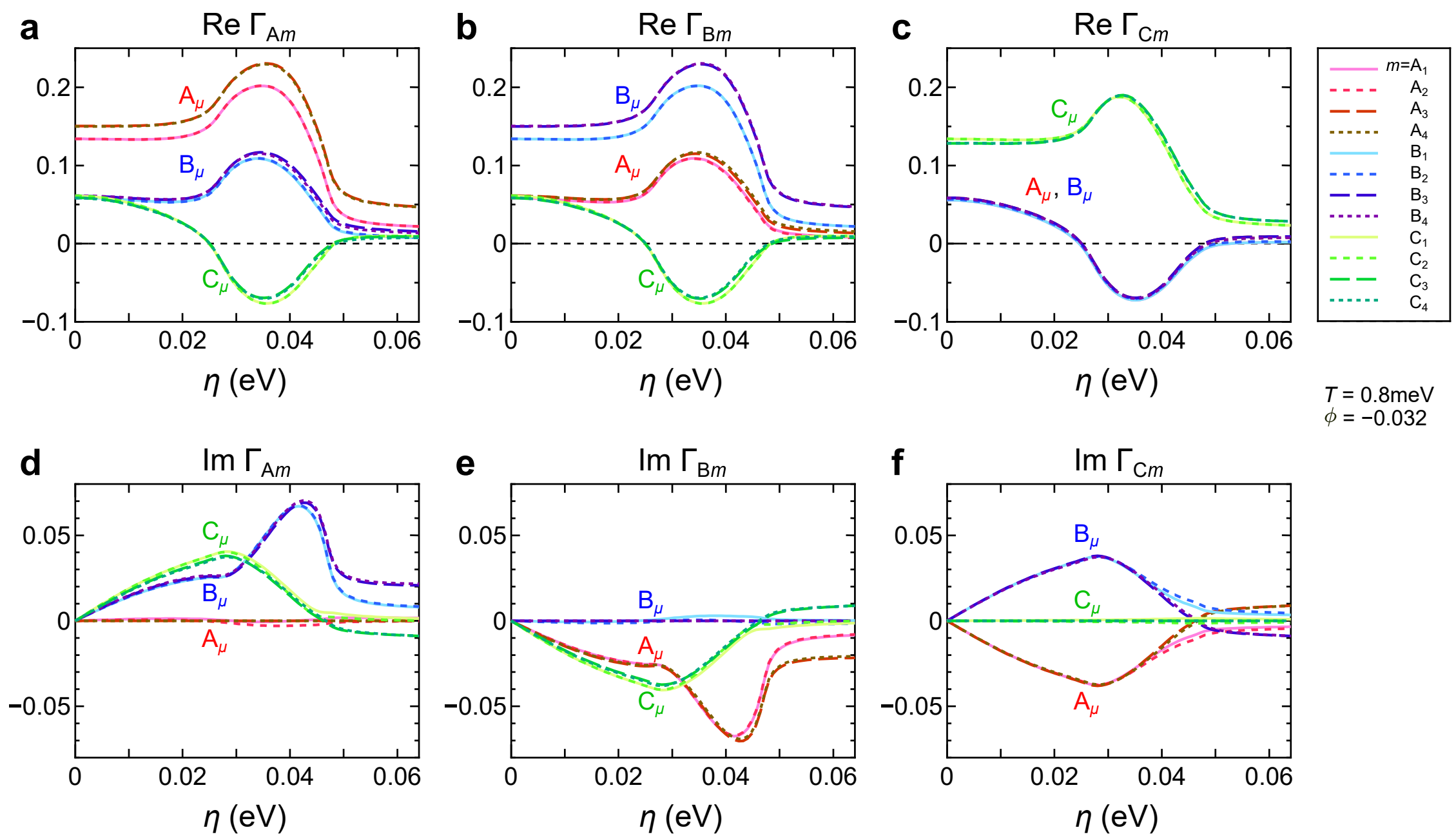

Supplementary Figure 2. **Kernel functions $\Gamma'_{lm} + i\Gamma''_{lm}$ in the LC+BO phase.** **a** Real part Γ'_{lm} and **b** imaginary part Γ''_{lm} of the kernel function as functions of η at l = A$_3$ (right), l = B$_4$ (center), and l = C$_3$ (right) for $\phi = 0.032$. Here, we denote A≡ A$_3$, B≡ B$_4$, C≡ C$_3$. It is found that $\Gamma_{\mathrm{A,L}_\nu}$, $\Gamma_{\mathrm{B,L}_\mu}$, and $\Gamma_{\mathrm{C,L}_\mu}$ are almost independent of μ. (L =A, B, or C.)

$$i\gamma''_{lm} = \delta^{(1,1)}_{lm}\eta\phi + \delta^{(3,0)}_{lm}\eta^3 + \cdots . \tag{6}$$

The diagrammatic expressions of $\hat{\beta}^{(m,n)}$ and $\hat{\delta}^{(m,n)}$ for $l = \mathrm{A}_\mu$ and $m = \mathrm{B}_\nu$ are given in Supplementary Figures 3 **a-b**.

Next, we estimate the increment of the eigenvalue, $\Delta\lambda$, in the presence of γ_{lm}. Here, we neglect the PDM in the 2×2 unit cell to simplify the discussion. Then, the second-order term of the GL free energy is approximately given by

$$\Delta\lambda = \sum_{\alpha\beta}^{\mathrm{A-C}} 4g\gamma_{\alpha\beta}\bar{\Delta}^*_\alpha\bar{\Delta}_\beta, , \tag{7}$$

$$\hat{\gamma} = \begin{pmatrix} d & a-ib & a+ib \\ a+ib & d & a-ib \\ a-ib & a+ib & d \end{pmatrix}, \tag{8}$$

where a, b, d are real coefficients, and b originates from the LC order. Here, α, β are the three sublattices in the original unit cell, and $\bar{\Delta}_\alpha$ is the normalized gap function. Thus, $\Delta\lambda_s = 4g(d + 2a)$ for s-wave state, and $\Delta\lambda_d = 4g(d - a \pm \sqrt{3}b)$ for chiral $d^\pm$-wave states. Therefore, the chiral d^+

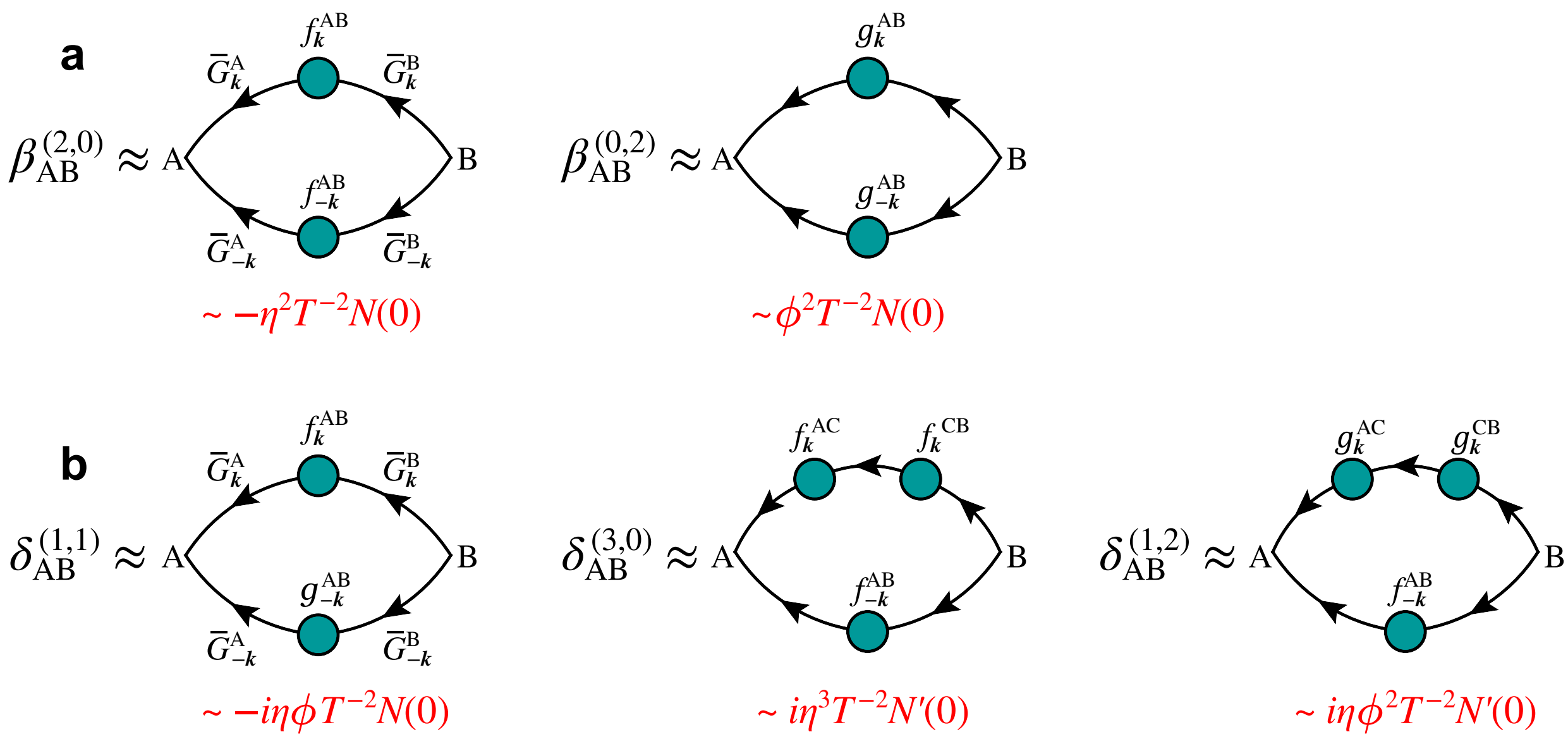

Supplementary Figure 3. **Diagrammatic expressions of GL coefficients**. Diagrammatic expressions of **a** $\beta^{(2,0)}_{\alpha\beta}$ and $\beta^{(0,2)}_{\alpha\beta}$, and those of **b** $\delta^{(1,1)}_{\alpha\beta}$, $\delta^{(3,0)}_{\alpha\beta}$, and $\delta^{(1,2)}_{\alpha\beta}$. Note that $\gamma'_{\alpha\beta} = \sum_{a,b} \beta^{(a,b)}_{\alpha\beta} \eta^a \phi^b$ and $i\gamma''_{\alpha\beta} = \sum_{a,b} \delta^{(a,b)}_{\alpha\beta} \eta^a \phi^b$.

wave SC state is strongly magnified when $b > 0$ in the LC phase.

Supplementary Note D: RPA analysis of kagome lattice Hubbard model

i. *Formalism*

In this paper, we investigate the mechanism of the nematic chiral superconducting (SC) state that emerges from the LC+BO coexisting phase in kagome metals. In the main text, we focus on the SC state mediated by the attractive charge-channel pairing interaction arising from BO fluctuations in addition to phonons, as suggested by recent experimental and theoretical studies [1, 4–6]. Within this mechanism, an anisotropic s-wave SC state is realized in the absence of the LC order. Interestingly, however, we demonstrate that a chiral SC state with a nonzero Chern number is stabilized once a reasonable LC order parameter ($\eta \sim$ 100K) is introduced.

In clean kagome metals, strong evidence for a TRS-breaking chiral SC state has been reported by several experimental techniques [7–11]. In contrast, an s-wave SC state without sign reversal is realized upon the introduction of impurities [4]. This observation supports the attractive charge-channel pairing mechanism proposed in the main text.

In contrast, a repulsive spin-channel pairing mechanism is also regarded as a promising candidate in kagome metals. To date, d-wave and s-wave singlet SC states have been proposed based on the spin-fluctuation mechanism [12, 13]. In addition, p wave and f wave triplet states have also been discussed [12]. To clarify the universality of the LC+BO-induced nematic chiral SC state proposed in this paper, it is important to examine whether the same mechanism is also applicable to spin-fluctuation-mediated superconductivity.

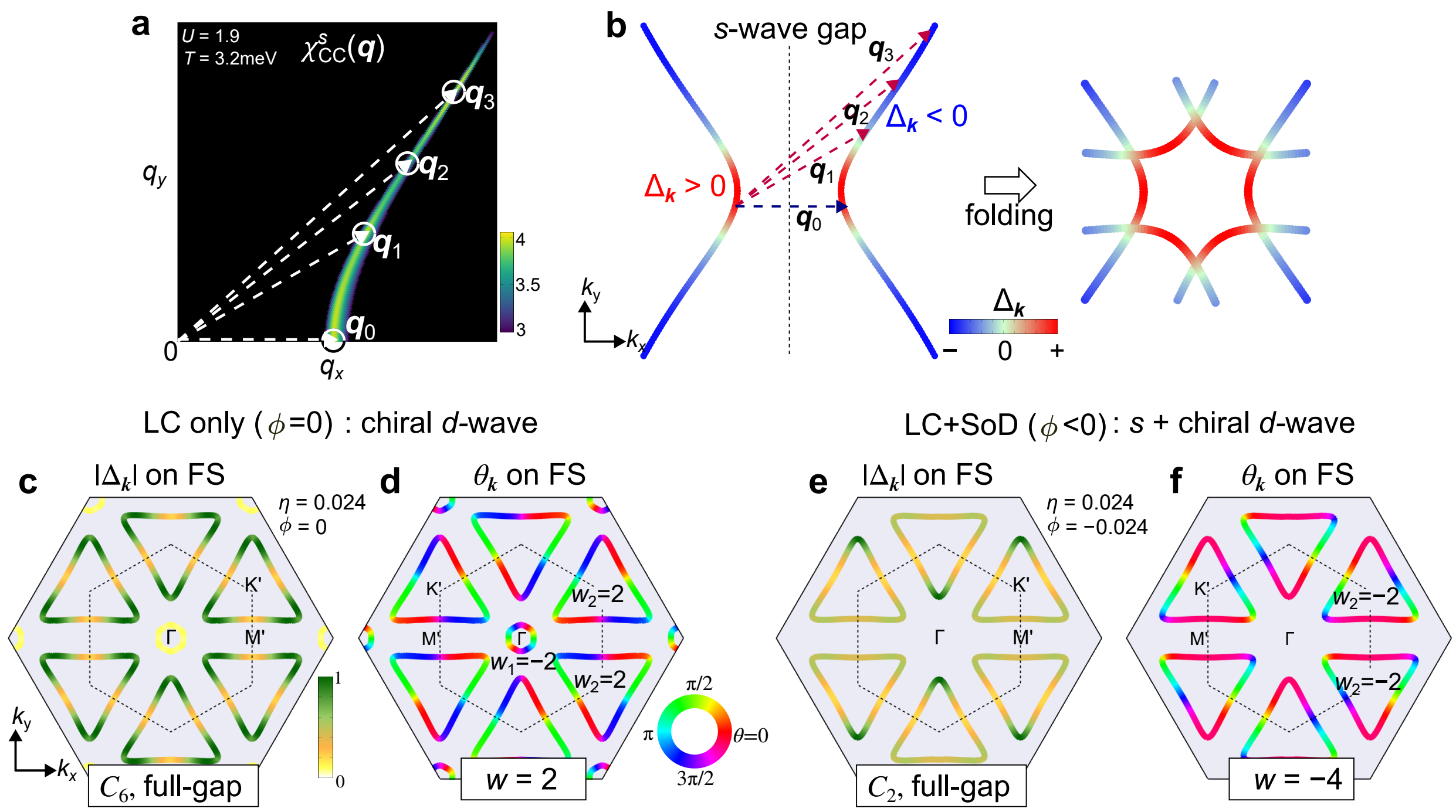

Supplementary Figure 4. **Fermi surface nesting and RPA spin susceptibility in the three-site unit-cell kagome lattice Hubbard model** ($\eta = \phi = 0$). **a** Static spin susceptibility $\chi^s_{\rm CC}(\mathbf{q})$ for $U = 1.92$ at $T = 0.8$meV. Here, $\chi^s_{\rm CC}(\mathbf{q}_0) \approx \chi^s_{\rm CC}(\mathbf{q}_3) \gtrsim \chi^s_{\rm CC}(\mathbf{q}_1) \gtrsim \chi^s_{\rm CC}(\mathbf{q}_2)$. $\chi^s_{\rm CC}(\mathbf{q})$ over the entire Brillouin zone is shown in Fig. 5 in the main text. **b** Nodal s-wave gap function on the Fermi surface $\Delta_{\mathbf{k}}$ mediated by spin fluctuations. Here, $\Delta_{\mathbf{k}}$ is positive near the vHS point at M and negative elsewhere, with accidental nodes appearing at their boundaries. The vectors $\mathbf{q}_1$, $\mathbf{q}_3$, $\mathbf{q}_2$ connects the SC gap functions with opposite signs, giving rise to the s-wave state with sign-reversal. Here, the regions of the Fermi surface with a large DOS near the vHS (i.e., small Fermi velocity) play a dominant role. We also present the s-wave gap function on the folded FS ($\eta = \phi = 0$). **c,d** Chiral d-wave gap function induced by the pure LC state $\eta = 0.024$ ($\phi = 0$): $|\Delta_{\mathbf{k}}|$ and $\theta_{\mathbf{k}}$. **e,f** Nematic chiral $s + d^{\pm}$ wave gap function induced by the LC+BO phase ($\eta = 0.024$, $\phi = -0.024$): $|\Delta_{\mathbf{k}}|$ and $\theta_{\mathbf{k}}$.

From now on, we perform the random-phase approximation (RPA) analysis, which has been

applied to kagome lattice Hubbard models. Here, we analyze the twelve-site unit-cell kagome lattice Hubbard model to calculate the spin fluctuation mediated superconductivity under the LC+BO phase. Using the irreducible susceptibility

$$\chi^0_{lm}(q) = -\frac{T}{N}\sum_{\mathbf{k}} G_{lm}(k+q)G_{ml}(k), \tag{9}$$

where $l, m = 1-12$. In the presence of on-site Coulomb interaction $U(>0)$, the spin and charge susceptibilities in the RPA are given by

$$\hat{\chi}^s(q) = \hat{\chi}^0(q)(\hat{1} - U\hat{\chi}^0(q))^{-1}, \tag{10}$$

$$\hat{\chi}^c(q) = \hat{\chi}^0(q)(\hat{1} + U\hat{\chi}^0(q))^{-1}, \tag{11}$$

where $q \equiv (\mathbf{q}, \omega_l)$ and $\omega_l \equiv 2\pi T l$. Here, $\hat{\chi}^s$ is magnified with finite U, and it diverges when the spin Stoner factor α_S, which is defined as the largest eigenvalue of $U\hat{\chi}^0(q)$, reaches unity. In contrast, $\hat{\chi}^c$ decreases for finite U. Using Supplementary Equations (10) and (11), the pairing interaction in the SC gap equation for the spin-singlet channel is given by

$$V^{\rm SC}_{lm}(q) = \frac{3}{2}U^2\chi^s_{lm}(q) - \frac{1}{2}U^2\chi^c_{lm}(q), \tag{12}$$

where $\chi^s_{lm}(q)$ ($\chi^c_{lm}(q)$) gives the repulsive (attractive) pairing interaction. The SC gap equation with $\mathbf{k}$-dependent gap function is

$$\begin{aligned}\lambda\Delta_{ll'}(k) = -T\sum_{p} V^{\rm SC}_{ll'}(k-p) \\ \times G_{lm}(p)G_{l'm'}(-p)\Delta_{mm'}(p),\end{aligned} \tag{13}$$

ii. *Numerical study*

To understand the spin-fluctuation-mediated SC state, we first study the three-site unit-cell kagome lattice Hubbard model without LC and BO. In the present RPA study, we set $t' = 0.12t$ and $n = 11.3$. Supplementary Figure 4 **a** demonstrates the obtained intra-sublattice spin susceptibility on sublattice C, $\chi^s_{\rm CC}(\mathbf{q}, 0)$, for $U = 1.92$ and $T = 3.2$meV ($\alpha_S = 0.91$). Due to the FS nesting involving sublattice C, $\chi^s_{\rm CC}(\mathbf{q}, 0)$ exhibits a line-shaped broad peak structures characterized by $\mathbf{q} \approx \mathbf{q}_n$ with $n = 0-3$. The spin fluctuations around $\mathbf{q} \approx \mathbf{q}_3$ play a crucial role in the SC pairing as discussed below. $\chi^s_{\rm CC}(\mathbf{q}, 0)$ over the entire Brillouin zone is shown in Fig. 5 in the main text.

Here, we solved the SC gap equation (13) for the three-site unit-cell kagome lattice model for $\eta = \phi = 0$. Supplementary Figure 4 **b** shows obtained the nodal s-wave gap function on the Fermi

surface $\Delta_{\mathbf{k}}$. Here, $\Delta_{\mathbf{k}}$ is positive near the vHS point at M and negative elsewhere, with accidental nodes appearing at their boundaries. The vectors $\mathbf{q}_3$, $\mathbf{q}_1$, $\mathbf{q}_2$ connects the SC gap functions with opposite signs, giving rise to the nodal s-wave state with sign-reversal. Here, the regions of the Fermi surface with a large DOS near the vHS (i.e., small Fermi velocity) play a dominant role. In deriving these s-wave and d-wave solutions, it is crucial to consider both (i) the optimization of the **k**- and ϵ_n-dependent gap function and off-Fermi-surface contribution. Here, the eigenvalue of the spin-triplet SC state is found to be much smaller than the s-wave SC state.

Next, we analyzed the SC gap equation for the twelve-site unit-cell kagome lattice model LC and BO. Supplementary Figures 4 **c** and **d** shows the chiral d-wave gap function induced by the pure LC state $\eta = 0.024$ ($\phi = 0$): **c** $|\Delta_{\mathbf{k}}|$ and **d** $\theta_{\mathbf{k}}$. The obtained nematic chiral SC state is very similar to that obtained by the attraction pairing mechanism explained in the main text. Therefore, the present LC-driven chiral SC state is robust, independently of the pairing mechanisms. Supplementary Figures 4 **e** and **f** shows the nematic chiral $s + d^{\pm}$ wave gap function induced by the nematic LC+BO phase ($\eta = 0.024$, $\phi = -0.024$): **e** $|\Delta_{\mathbf{k}}|$ and **f** $\theta_{\mathbf{k}}$. Thus, prominent nematic chiral SC gap is realized in the LC+BO phase, consistently with the analysis of the attractive pairing (g) model performed in the main text.

To summarize, we have studied the spin-fluctuation-mediated SC state in kagome lattice Hubbard model. In the absence of LC and BO, both a nodal s-wave state and doubly-degenerated d-wave states are obtained thanks to the optimization of the gap function $\Delta(\mathbf{k}, \epsilon_n)$ and the inclusion of off-Fermi-surface scattering processes. In the LC+BO phase, prominent nematic chiral SC state is obtained over a wide parameter range. The obtained results are essentially similar to the results derived from the charge-channel attraction mechanism. Therefore, the LC+BO induced nematic chiral SC state proposed in the main text is expected to emerge quite generally, irrespective of the details of the Cooper pairing mechanism. We stress that the nodal s-wave state given in Fig. 5 **b** in the main text should be fragile against impurities. Thus, impurity-induced fully gapped s-wave state observed in kagome metals [4] can be naturally understood in terms of the charge-channel attraction mechanism.

Supplementary Note E: chiral *d*-wave superconductivity in three-dimensional two-orbital kagome metal model

i. *Multiorbital bandstructure and Fermi surfaces*

In the main text, we analyzed the d_{xz}-orbital kagome lattice model. This model reproduces the "p-type" Fermi surface of kagome metal AV_3Sb_5. In addition, in kagome metals, the d_{yz}-orbitals give the "m-type" Fermi surface, where each of three vHS points is composed of two sublattices. The bandwidth of p-type conduction band is narrower than that of m-type band. In addition, m-type Fermi surface has three-dimensional character, which weakens the singularity of the vHS. Therefore, electron correlations strongly develop in the p-type conduction band according to previous theoretical analyses. For this reason, in the present paper, we introduce the LC and BO only for d_{xz}-orbitals.

To perform more realistic analysis, here we study the three-dimensional d_{xz}+d_{yz} orbital kagome lattice model shown in Supplementary Figures 5 **a** and **b**, which has been introduced in our previous study of the eMChA [14]. We set the model parameters as $t_{xz} = -0.8$, $t'_{xz} = -0.128$, $t_{yz} = -1.6$, $t'_{yz} = 0$, $t_{xz-yz} = \pm 0.08$, $E_{yz} - E_{xz} = 3.8$, and $t_{yz\perp} = t'_{yz\perp} = 0.02$ in units eV to reproduce the first principles 30-orbital unit-cell(or 13-orbital unit-cell) tight-binding model [15, 16]. The electron filling per twelve V sites is $n = 17.84$ for $xz + yz$ orbitals, and $n_{xz} = 11.84$ for xz orbitals. Supplementary Figure 5 **c** shows the band-structure of two-orbital kagome lattice model. The present model captures the characteristic 3D bandstructure of kagome metals near $E \sim E_{\rm F}$ and $E_{\rm vHS}$, including both p- and m-type band dispersion.

We stress that the p- and m-type bands hybridize via t_{xz-yz}, leading to a magnification of the LC-induced orbital magnetization (OM) $M_{\rm orb}$ [3] as well as the eMChA [14, 15]. The band reconstruction and pseudogap formation are more clearly observed around $E \sim E_{vHS}^{xz}$ and $E_{\rm Dirac}$ (see below).

Supplementary Figures 6 **a** and **b** represent the original Fermi surfaces on the $k_z = 0$ plane and $k_z = \pi$ plane, respectively. Here, p-type FS and m-type FS are mainly composed of the d_{xz}-orbital and d_{yz}-orbital, respectively. The *p*-type FS is almost two-dimensional, while the *m*-type FS shows a non-negligible three-dimensional character, due to the inter-layer d_{yz}-orbital hopping integral $t_{yz\perp} = 0.02$.

To estimate a reliable value of $t_{xz\text{-}yz}$, we focus on the band structure near the van Hove energy.

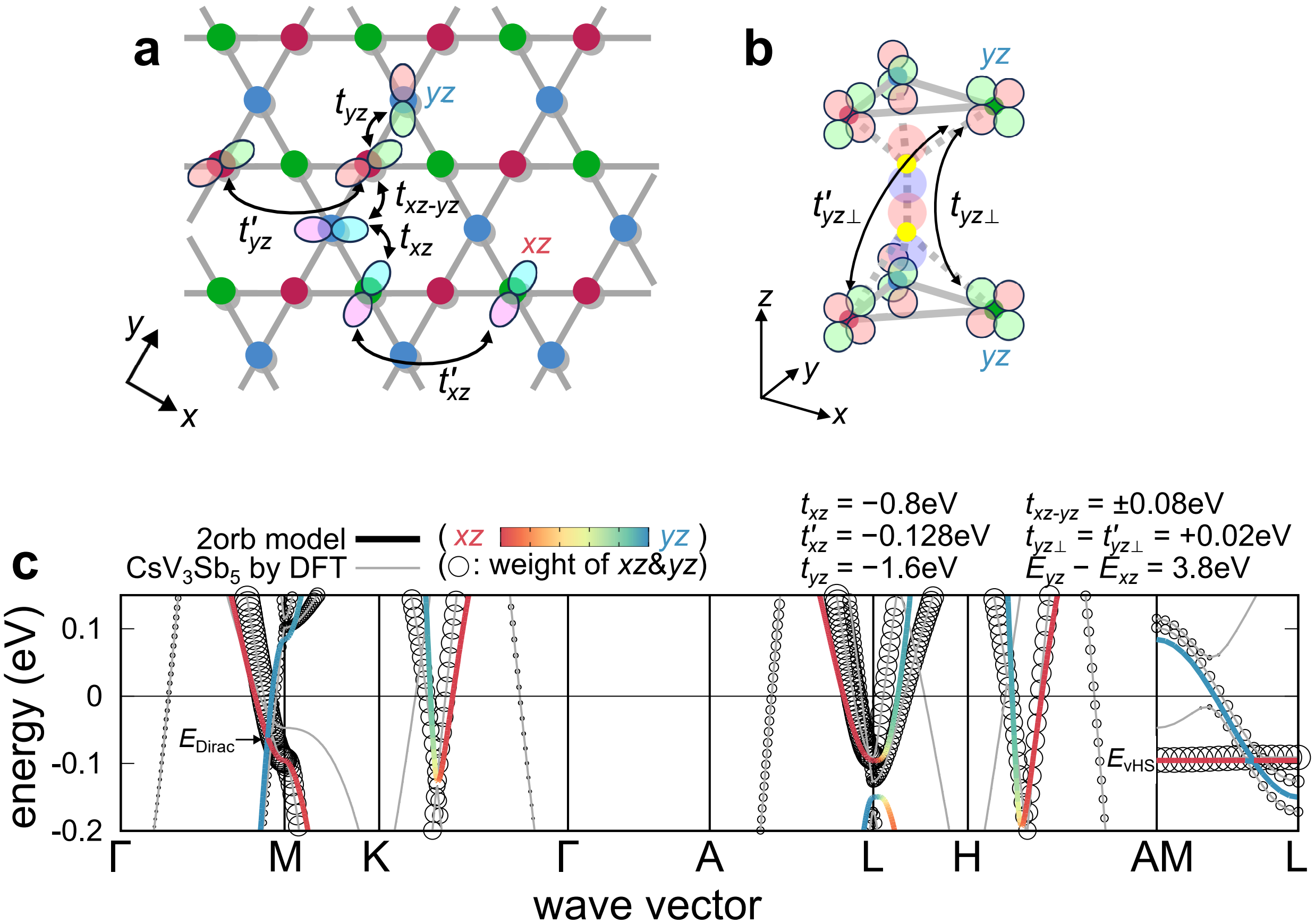

Supplementary Figure 5. **3D kagome lattice model parameters**. **a** Intralayer hopping integrals and **b** inter-layer hopping integrals in the present (d_{xz}, d_{yz})-orbital kagome metal model; See Fig. 5 of Ref. [15]. **c** The 3D bandstructures of the present kagome metal model near the Fermi level. The Red and blue colors represent the weight of the d_{xz}- and d_{yz}-orbitals, respectively. The open circles denote the *ab initio* band structure, with the radius of each circle indicating the $d_{xz} + d_{yz}$-orbital weight.

Supplementary Figure 6 **c** presents the constant-energy contours at $E = E_{\mathrm{Dirac}}$ on the $k_z = 0$ plane, comparing the two-orbital model (left) with the DTF model (right). Here, E_{Dirac} is defined in Supplementary Figure 5**c**, and is slightly higher than E_{vHS}. In both models, inter-orbital hybridization induced by $t_{xz\text{-}yz}$ leads to a reconstruction of the d_{xz}- and d_{yz}-orbital bands, such that major portions of the contours are gapped out. Given the close similarity between the contours obtained from the two models, we conclude that the present choice of the model parameter $t_{xz\text{-}yz} = 0.08$ is reasonable. We note that the giant eMChA observed in CsV_3Sb_5 can be naturally explained within the two-orbital model with $t_{xz\text{-}yz} = 0.08$ [15].

Supplementary Figures 6 **d** and **e** show the folded Fermi surfaces for both $\phi, \eta \to 0$, on the

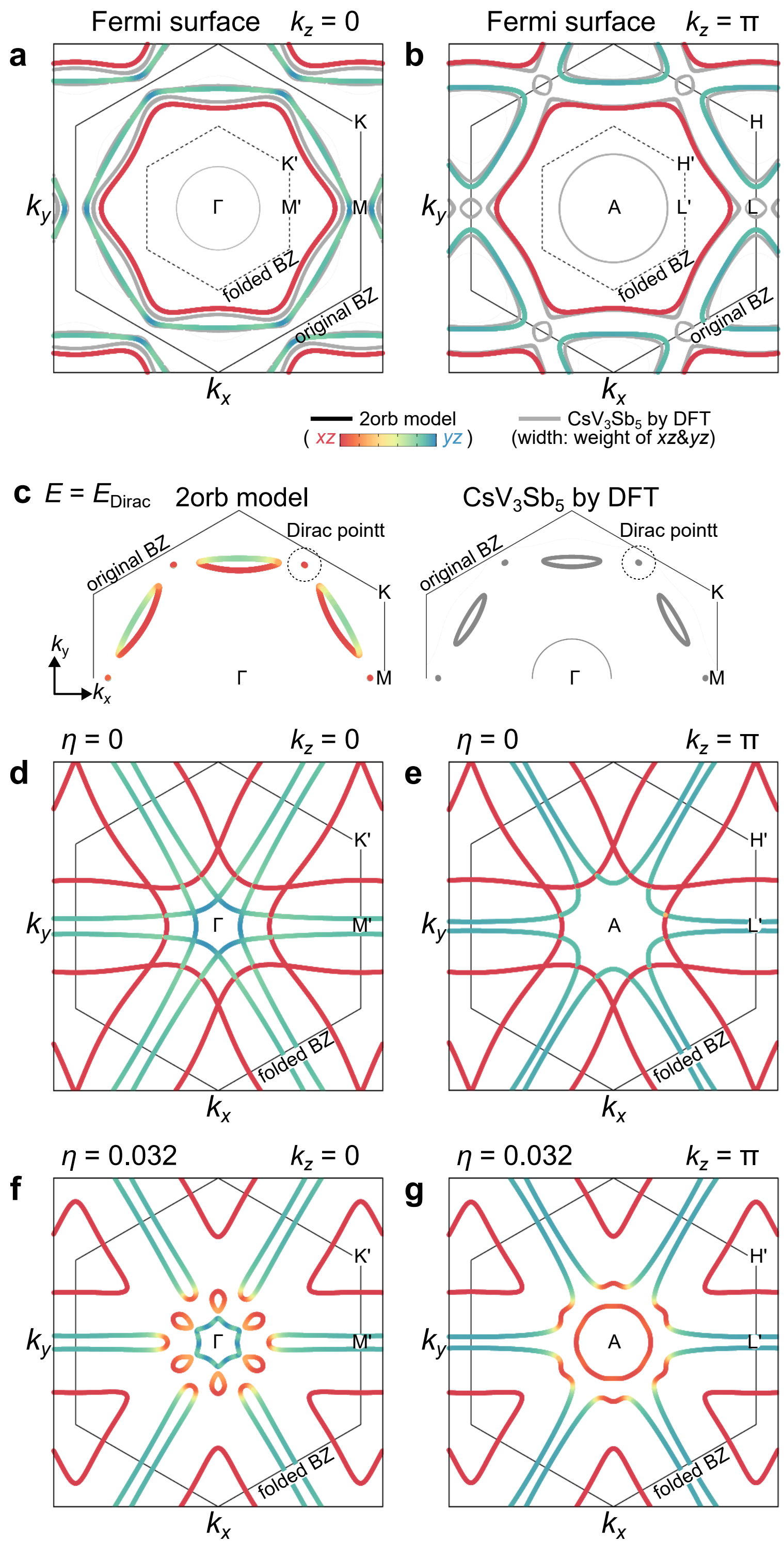

Supplementary Figure 6. **Fermi surfaces of 3D kagome lattice model** ($\phi = 0$). The FSs for $\eta = 0$ in the unfolded BZ on the **a** $k_z = 0$ plane and **b** $k_z = \pi$ plane. **c** Constant-energy contours at $E = E_{\mathrm{Dirac}}$ on the $k_z = 0$ plane: (left) two-orbital model; (right) DTF model. They are equivalent to those in **a** and **b** in the unfolded BZ. **f,g** The FSs for $\eta = 0.032$ in the folded BZ on the **f** $k_z = 0$ plane and **g** $k_z = \pi$ plane.

$k_z = 0$-plane and $k_z = \pi$-plane, respectively. These FSs are modified to Supplementary Figures 6 **f** and **g** for $\eta = 0.032$. Here, the d_{xz}-orbital type FSs hybridize and form a hole-pocket around Γ-point, as we explained based on the single orbital model in the main text. Importantly, the d_{yz}-orbital type FSs also contribute to the LC-induced FS reconstruction, because of the finite (d_{xz}, d_{yz})-orbital hybridization in the original FSs due to t_{xz-yz}. This fact magnify the orbital magnetization $M_{\rm orb}$ [3].

ii. *Numerical study*

Here, we solve the SC gap equation for the 3D (d_{xz}, d_{yz})-orbital model. The LC order is introduced only on the d_{xz}-orbital because it is expected to originate from the strong correlations in the p-type Fermi surface. Supplementary Figure 7 **a** shows the obtained eigenvalues of the SC gap equation as functions of η, when the d_{xz}-orbital electron filling is $n_{xz} = 11.84$. The derived parameter range for the chiral SC state is comparable to that in the 2D d_{xz}-orbital model, which is shown in Supplementary Figure 7 **b**.

Supplementary Figure 7 **c** shows the obtained SC eigenvalues as functions as functions of n_{xz}, when the LC order parameter is $\eta = 0.032$. The derived results are comparable to those in the 2D d_{xz}-orbital model, which is shown in Supplementary Figure 7 **d**.

To summarize, we find that the LC-induced chiral SC is realized within the three-dimensional $d_{xz} + d_{yz}$ orbital model, despite the reduction of the DOS caused by the significant inter-orbital hybridization. In Supplementary Figure 7, the interaction g and the LC order parameter η are introduced only on the d_{xz} orbitals. However, we have confirmed that introducing g and η also on the d_{yz} orbitals yields essentially the same results due to the large d_{xz}-orbital DOS at $E_{\rm F}$. Thus, the present study strongly indicates the robustness of the LC-induced chiral superconductivity mechanism.

Supplementary Note F: Hierarchy of Energy Scales in Kagome Metals

i. *Emergence of the s-wave SC and d-wave SC instabilities derived from the parquet RG study*

The experimental hierarchy of temperature scales "$T_{\rm BO} \gtrsim T_{\rm LC} \gg T_{\rm s-SC} \sim T_{\rm d-SC}$" is naturally obtained by the parquet RG analysis presented in the Supplemental Information of Ref. [2], when

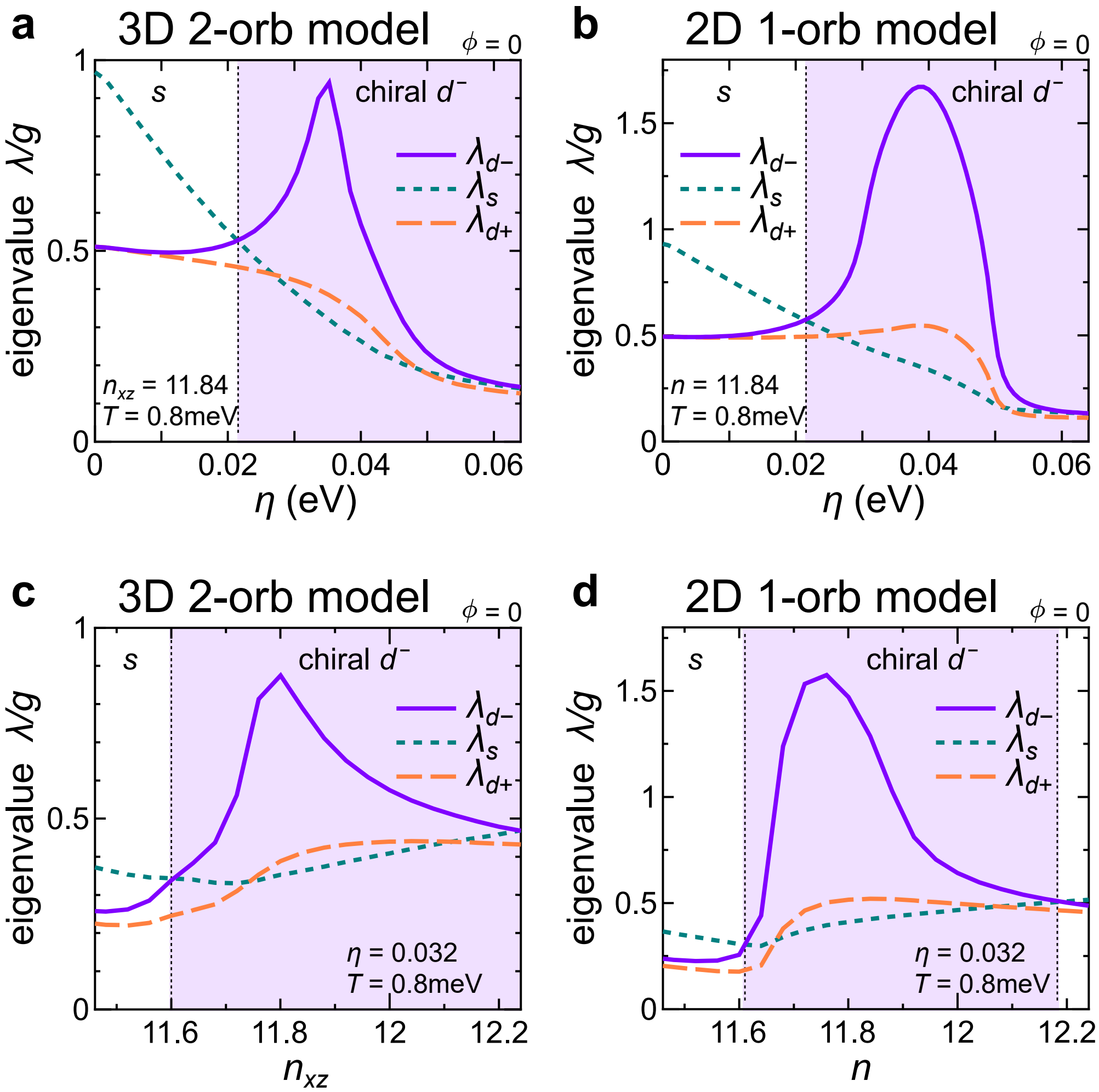

Supplementary Figure 7. **LC-induced chiral d-wave SC states in 3D kagome lattice model ($\phi = 0$).** **a** The first to third largest eigenvalues of the SC gap equation as functions of η in the 3D ($d_{xz} + d_{yz}$)-orbital kagome lattice model. For comparison, numerical results in the 2D d_{xz}-orbital model are shown in **b**. **c** The first to third largest eigenvalues as functions of n_{xz} (per V atom) in the 3D ($d_{xz} + d_{yz}$)-orbital kagome lattice model. For comparison, numerical results in the 2D d_{xz}-orbital model are shown in **d**.

the inter-site attractive interaction v is much smaller than the Coulomb repulsion U. In Supplementary Figures 8 **a** and **b**, we show the numerical results of **a** the renormalized interactions $g_1 \sim g_4$ and **b** the instabilities for the channel X, G_X (X = rCDW, iCDW, rSDW, iSDW, s-SC and d-SC) for $U = 0.9$ and $v = 0.6$. The horizontal axis denotes the RG parameter $z = \ln^2(E_0/T)$, where E_0 is the cutoff-energy ($E_0 \sim |t|$) and T is the temperature: $z = 12$ corresponds to $T = E_0/30$. A detailed explanation of the parquet RG theory is presented in Supplementary Note 5 of Ref. [2]. In Supplementary Figure 8 **b**, the obtained instabilities G_X shown $G_{\mathrm{rCDW}} \sim G_{\mathrm{iCDW}} \gg G_{\mathrm{s-SC}} \sim G_{\mathrm{d-SC}}$

for $z > 10$: $G_{\rm rCDW}$ ($G_{\rm iCDW}$) represents the BO (LC) instability, and $G_{\rm s-SC}$ ($G_{\rm d-SC}$) represents the s-wave (d-wave) SC instability. Essentially similar numerical results are obtained for $U = 3.0$ and $v = 0.6$; see Ref. [2]. Under these conditions, $T_{\rm d-SC} > T_{\rm s-SC}$ can be realized in the LC phase.

Thus, the parquet RG analysis provides a consistent understanding of the relationship among the energy (temperature) scales of the three orders – bond order, loop-current order, and superconductivity – despite their common origin in the charge-channel interactions. The increment of $G_{\rm s-SC} = -g_4 - 2g_3$ and $G_{\rm d-SC} = -g_4 + g_3$ originates from the large negative value of g_4 obtained in Supplementary Figure 8 **a**. Because negative g_4 corresponds to an intra-sublattice attraction, this result indicates that the present on-site attractive (g) model is supported by the parquet RG analysis. However, unfortunately, it is difficult to perform a microscopic calculation of the pairing interaction g in the 2×2 LC+BO phase.

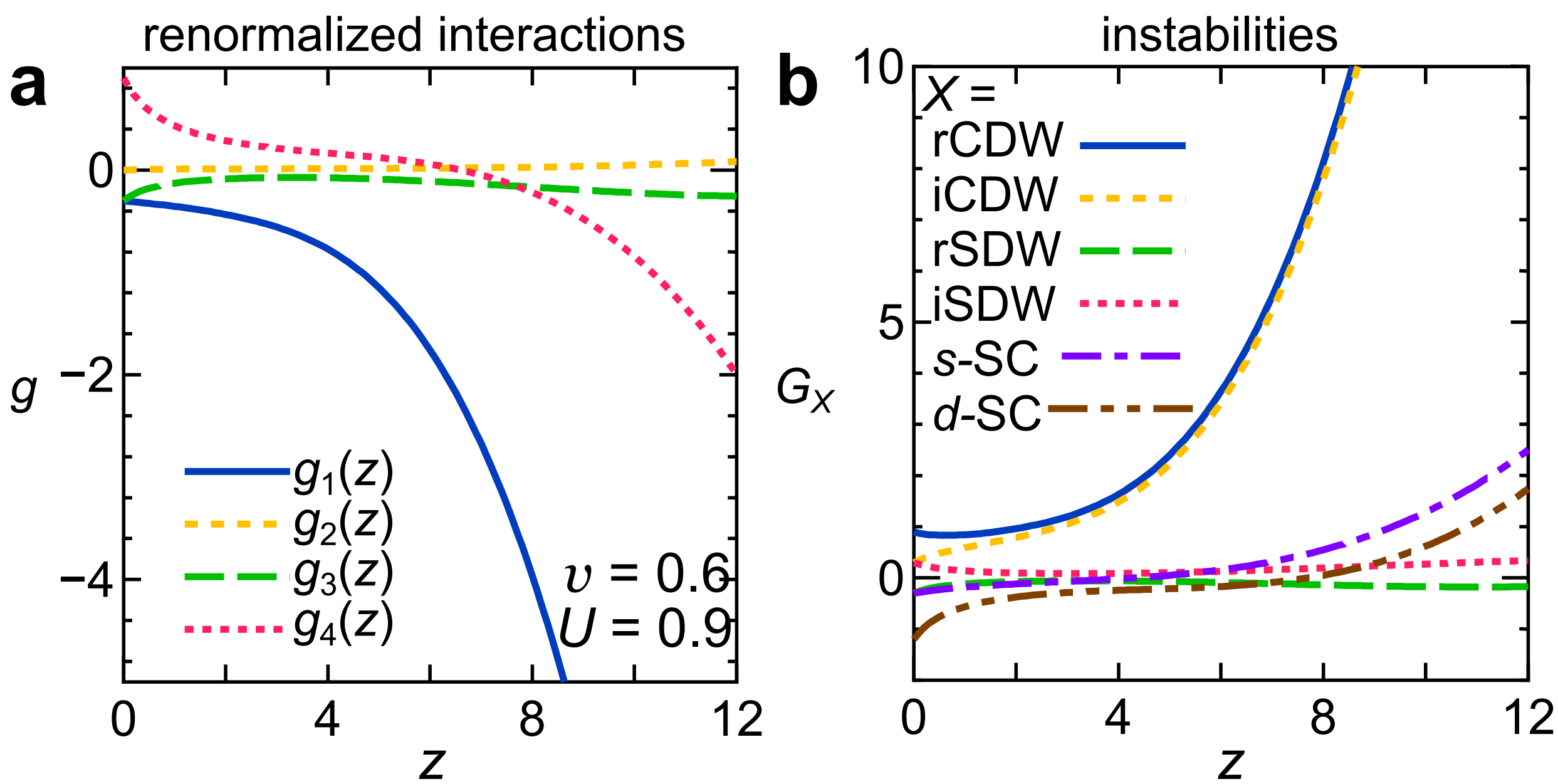

Supplementary Figure 8. **Parquet RG study for kagome metal model with $U = 0.9$ and $v = 0.6$.** **a** Renormalized interactions $g_1 \sim g_4$ as functions of the renormalization parameter z. **b** Instabilities for the channel X, G_X (X = rCDW, iCDW, rSDW, iSDW, s-SC and d-SC), given by $g_1 \sim g_4$.

ii. *Magnitude of the bond order* ϕ

Next, we discuss the magnitude of the order parameters. In the folded Brillouin zone of the 2×2 bond-ordered state, the three van Hove singularities (vHSs) at the M points ($E_{\rm vHS} \simeq -0.05$

to -0.1 eV) are folded onto the Γ point, and their degeneracy is lifted by the bond-order parameter ϕ. As a result, the energies split into a doubly degenerate level at $E_{\mathrm{vHS}} - 2\phi$ and a nondegenerate level at $E_{\mathrm{vHS}} + 4\phi$.

ARPES experiments have shown that, below the bond-order transition temperature T_{BO}, the bands near the vHS are shifted and/or reconstructed on an energy scale of approximately 80 meV. In this work, we take $\phi \sim 30$ meV, for which the energy splittings 2ϕ and 4ϕ are consistent with the experimentally observed reconstruction scales [17, 18]. At this value, $2\phi/T_{\mathrm{BO}} \sim 6$, which is compatible with the expectation from an intermediate-coupling BCS-type scenario.

iii. *Magnitude of the LC order* η

The transition temperature of the loop-current (LC) order in AV_3Sb_5, T_{LC}, remains controversial. Switchable nonreciprocal transport measurements (electrical magneto-chiral anisotropy) [14] and resonator-based tuning measurements [19] have reported broken time-reversal symmetry at $T^* \sim 35$ K, suggesting $T_{\mathrm{LC}} \approx 35$ K. On the other hand, some experiments indicate higher LC transition temperatures, namely 50–60 K from μSR measurements and approximately 120 K from magnetic torque experiments [20].

As discussed above, the order parameters η and ϕ are expected to be of comparable magnitude; however, their precise experimental values remain unsettled. Taking this uncertainty into account, we perform calculations assuming $\eta, \phi \sim 20$–30 meV. As shown in Fig. 5 in the main text, we emphasize that chiral superconductivity can be realized even in the regime $\eta < \phi$.

iv. *Magnitude of the superconducting gap*

In AV_3Sb_5, the superconducting transition temperature T_{SC} is at most ~ 3 K, which is more than one order of magnitude smaller than T_{BO} and T_{LC}. Correspondingly, the superconducting gap Δ is also more than one order of magnitude smaller than η and ϕ. Therefore, the changes of η and ϕ caused by the SC gap are expected to be negligible.

From the viewpoint of the hierarchy of energy scales, this justifies our treatment of η and ϕ as fixed (mean-field) parameters in solving the superconducting gap equation in the present study.

Supplementary Note G: Folded FS and unfolded FS

In the 2×2 BO phase, the Fermi surface is folded into the reduced Brillouin zone and reconstructed by the BO order, as shown in Supplementary Figure 9 **a** for $\phi = -0.032$eV. As a consequence of this BO-induced Fermi-surface reconstruction, a hole pocket appears around the Γ point. This hole pocket is a direct consequence of the geometry of the d_{xz}-orbital Fermi surface and necessarily emerges over a wide parameter range, $|\phi| \lesssim 0.05$eV.

This folded Fermi surface can be unfolded into the original Brillouin zone by approximately restoring translational symmetry. Supplementary Figure 9 **b** shows the resulting unfolded Fermi surface, which corresponds to experimentally observed Fermi surface in the ARPES measurements. Thus, apart from a hybridization gap, the unfolded Fermi surface remains essentially unchanged, and finite experimental resolution and lifetime broadening cause the ARPES-observed Fermi surface in the 2×2 density-wave phase to appear similar to the original one.

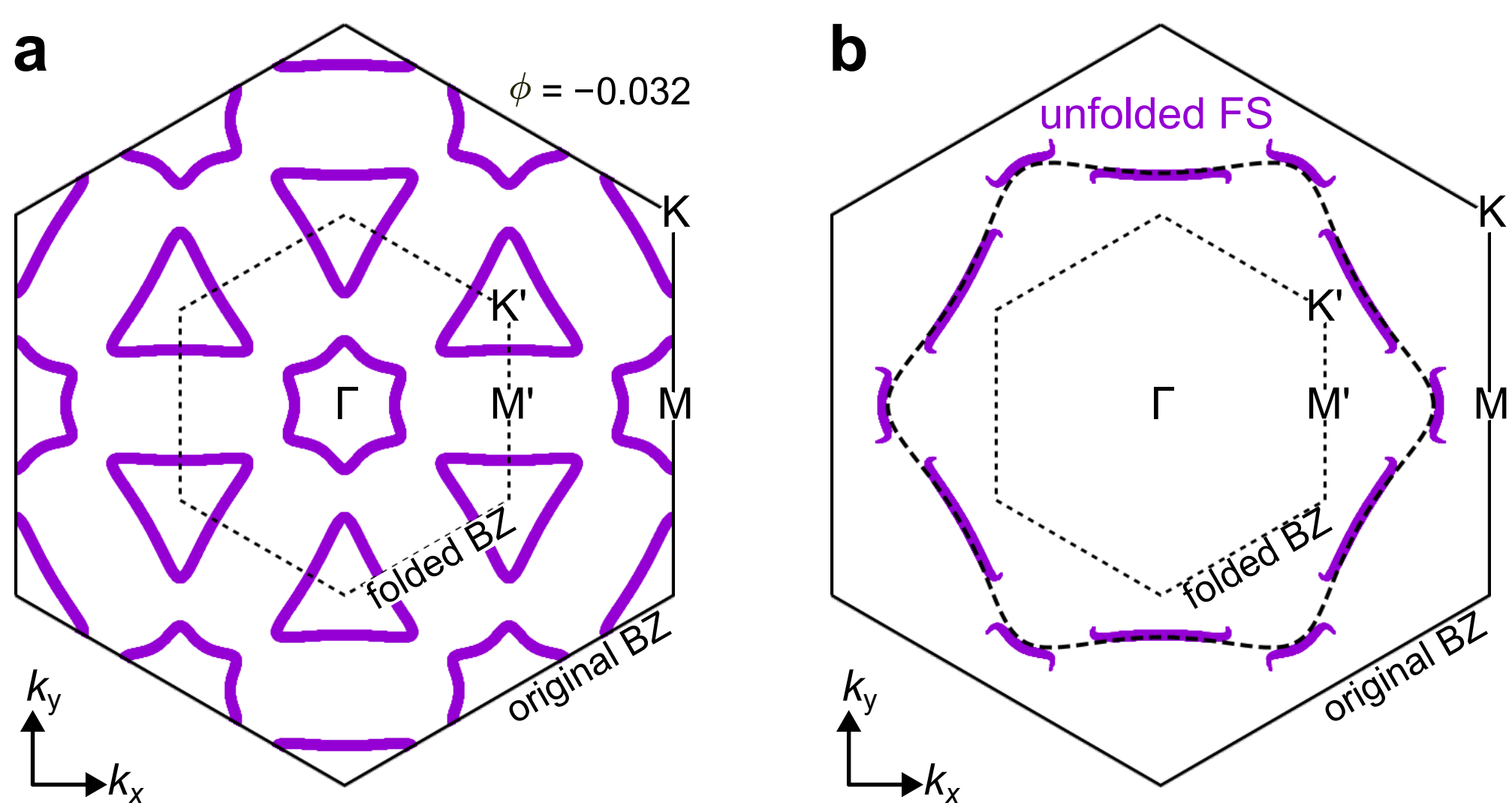

Supplementary Figure 9. **Folded FS and unfolded FS**. **a** Folded Fermi surface into the reduced Brillouin zone for $\phi = -0.032$. A hole pocket appears around the Γ point. **b** Unifded Fermi surface shown in **a** into the original Brillouin zone, which corresponds to experimentally Fermi surface in the ARPES measurements. The dotted line represents the original Fermi surface.

Supplementary Note H: Derivation of the GL free energy: OM-chirality coupling mechanism

i. *Why the LC order enhances chiral d-wave superconductivity*

In this paper, we analyze the superconducting gap equation in the loop-current (LC) phase of kagome metals and demonstrate that one of the chiral d-wave superconducting states, $d_\pm = d_{x^2-y^2} \pm id_{xy}$, is selectively enhanced by the LC order and eventually dominates over the s-wave superconducting state. As a compelling physical explanation, we newly propose a scenario focusing on the uniform orbital magnetization (OM) $M_{\rm orb}$ induced by the LC order.

The microscopic expression of $M_{\rm orb}$ is given by [21].

$$M_{\rm orb} = \frac{1}{N}\sum_{\mathbf{k}} M^{\rm orb}_{\mathbf{k}}, \tag{14}$$

$$M^{\rm orb}_{\mathbf{k}} = \frac{1}{\pi}\sum_{b}\{m^b_{\mathbf{k}} f(\epsilon_{b,\mathbf{k}}) -\Omega^b_{\mathbf{k}} T \ln(1+e^{-(\epsilon_{b,\mathbf{k}}-\mu)/T})\}, \tag{15}$$

where b is the band index, $\epsilon_{b,\mathbf{k}}$ is the Bloch band dispersion, and $f(\epsilon)$ is the Fermi function. At $T=0$, the integrand of Supplementary Equation (15) becomes $(m^b_{\mathbf{k}} + (\epsilon_{b,\mathbf{k}}-\mu)\Omega^b_{\mathbf{k}}) f^{T=0}(\epsilon_{b,\mathbf{k}})$. $m^b_{\mathbf{k}}$ is the self-rotating orbital magnetic moment of the electron at $\mathbf{k}$ in the b-th band, and $\Omega^b_{\mathbf{k}}$ is the Berry curvature. They are given by

$$m^b_{\mathbf{k}} = \frac{1}{2}\sum_{b'(\neq b)} \frac{{\rm Im}\{v^x_{b,b',\mathbf{k}} v^y_{b',b,\mathbf{k}} - v^y_{b,b',\mathbf{k}} v^x_{b',b,\mathbf{k}}\}}{\epsilon_{b,\mathbf{k}}-\epsilon_{b',\mathbf{k}}}, \tag{16}$$

$$\Omega^b_{\mathbf{k}} = \sum_{b'(\neq b)} \frac{{\rm Im}\{v^x_{b,b',\mathbf{k}} v^y_{b',b,\mathbf{k}} - v^y_{b,b',\mathbf{k}} v^x_{b',b,\mathbf{k}}\}}{(\epsilon_{b,\mathbf{k}}-\epsilon_{b',\mathbf{k}})^2}, \tag{17}$$

where the off-diagonal velocity $v^\nu_{b,b',\mathbf{k}}$ ($\nu = x,y$) in the band representation is given by the unitary transform of $\partial t^{l,m}_{\mathbf{k}}/\partial k_\nu$ in the sublattice representation.

The Ginzburg–Landau (GL) expression for $M_{\rm orb}$ originating from normal-state electrons in kagome metals was derived in Ref. [15] based on symmetry arguments and diagrammatic calculations:

$$M_{\rm orb} = m_1(-\eta_1\phi_1 + \eta_2\phi_2 + \eta_3\phi_3) +m_2(-\eta_1\phi_2\phi_3 + \eta_2\phi_3\phi_1 + \eta_3\phi_1\phi_2) -m_3\,\eta_1\eta_2\eta_3 + \cdots, \tag{18}$$

where the coefficients m_1, m_2, and m_3 are functions of the filling n and temperature T, and they satisfy the relation $m_2 = -m_3$. (Supplementary Equation (18) is equivalent to that in Ref. [3], up to a sign change of η_1 reflecting a different LC form factor f_{ij}^{AB}.)

Since Cooper pairs in the $d_\pm$ wave superconducting states carry orbital angular momentum ± 2, a finite M_{orb} produces a Zeeman-like energy splitting that stabilizes one of the two chiral states. Indeed, here we derive the following OM-chirality coupling energy:

$$F_{\text{OM-ch}} \propto -\bar{M}_{\mathrm{orb}} \left(|\varphi_{d-}|^2 - |\varphi_{d+}|^2 \right), \tag{19}$$

where $\varphi_\pm$ denote the order-parameter amplitudes of the $d_\pm$ wave superconducting states. The coefficient $\bar{M}_{\mathrm{orb}}$ is expressed as

$$\begin{aligned} \bar{M}_{\mathrm{orb}} = {} & \bar{m}_1(-\eta_1\phi_1 + \eta_2\phi_2 + \eta_3\phi_3) \\ & + \bar{m}_2(-\eta_1\phi_2\phi_3 + \eta_2\phi_3\phi_1 + \eta_3\phi_1\phi_2) \\ & - \bar{m}_3\, \eta_1\eta_2\eta_3 + \cdots . \end{aligned} \tag{20}$$

In the Supplementary Note H **iii**, we derive the expression given in Supplementary Equation (20). In the Supplementary Note H **iv**, an approximate relation $\bar{M}_{\mathrm{orb}} \sim M_{\mathrm{orb}}$ is numerically obtained.

In summary, the M_{orb} induced by the LC (and bond-order) phase stabilizes one of the two chiral $d_\pm$ wave superconducting states via its orbital angular momentum, leading to the realization of chiral d-wave superconductivity.

ii. *Why the superconducting chirality is reversed under $\phi \to -\phi$*

When η and ϕ are sufficiently small, M_{orb} is dominated by the quadratic term ($\bar{m}_1\eta\phi$) in Supplementary Equation (20) and thus changes sign under the transformation $\phi \to -\phi$. Consequently, the chirality of the d-wave superconducting state induced by $F_{\text{OM-ch}}$ is reversed. This behavior is confirmed in Supplementary Figure 10.

An intuitive explanation for the sign reversal of M_{orb} under $\phi \to -\phi$ is as follows. The pure LC state of kagome metals $\eta \neq 0$ ($\phi = 0$) is a kind of ferrimagnetic state: the local magnetization is linear in η, but the fluxes in the $+z$ and $-z$ directions cancel each other, resulting in the absence of the η-linear contribution to M_{orb}. When bond order (BO) develops ($\phi \neq 0$), this cancellation becomes incomplete, and an η-linear term emerges. As a result, in the BO phase, a small η can induce a large chiral superconducting component.

iii. *Outline of the derivation of Supplementary Equations (19) and (20)*

To understand Supplementary Equations (19) and (20) in more detail, we clarify the relation between the superconducting gap equation analyzed in this paper and the phenomenological GL free-energy theory. The coefficients $\bar{m}_1$, $\bar{m}_2$, and $\bar{m}_3$ in $\bar{M}_{\rm orb}$ are functions of n and T, and satisfy $\bar{m}_2 = -\bar{m}_3$. Here, we explain that these coefficients are derived from the diagrammatic calculations of $i\gamma''_{lm}$ shown in Supplementary Figure 3 of the main text.

As we explained in the Supplementary Note C, the kernel of the linearized superconducting gap equation, Γ_{lm}, is expanded in terms of the order parameters η and ϕ as $\Gamma_{lm} = \Gamma^0_{lm} + \gamma_{lm}$, $\gamma_{lm} = \sum_{a,b} \gamma^{a,b}_{lm}\, \eta^a \phi^b$ $(a, b = 0, 1, 2, \ldots)$. (For simplicity, we set $\boldsymbol{\eta} = \eta(1,1,1)$ and $\boldsymbol{\phi} = \phi(1,1,1)$, following the Supplementary Note C.) Since η and ϕ carry momenta $\pm\mathbf{p}_n$ $(n = 1, 2, 3)$, momentum conservation requires $\gamma^{a,b}_{lm} = 0$ for $a+b = 1$. On the other hand, $\gamma^{a,b}_{lm}$ can become finite for $a+b = 2$ and 3, considering the relations $2\mathbf{p}_n = \mathbf{0}$ and $\sum_n \mathbf{p}_n = \mathbf{0}$ $(n = 1, 2, 3)$. When a is odd (even), $\gamma^{a,b}_{lm}$ is purely imaginary (real). Note that the relation $\gamma_{lm} = (\gamma_{ml})^*$ holds.

Let λ_ρ denote an eigenvalue of the matrix $g\Gamma_{lm}$, and let $(\bar{\Delta}^\rho_1, \bar{\Delta}^\rho_2, \ldots, \bar{\Delta}^\rho_{12})$ be the corresponding normalized eigenvector. Here, ρ denotes the irreducible representation. When $\lambda_\rho > 1$, the superconductivity is realized. By introducing the gap amplitude φ_ρ, the superconducting gap is written as $\Delta^\rho_l = \varphi_\rho\, \bar{\Delta}^\rho_l$.

Based on the gap equation, the change in the eigenvalue due to γ_{lm} is $\Delta\lambda_\rho = \sum_{l,m} \gamma_{lm} (\bar{\Delta}^\rho_l)^* \bar{\Delta}^\rho_m$. It can be expanded with respect to η and ϕ as $\Delta\lambda_\rho = \sum_{a,b} D^{a,b}_\rho \eta^a \phi^b$, where $D^{a,b}_\rho = \sum_{l,m} \gamma^{a,b}_{lm} (\bar{\Delta}^\rho_l)^* \bar{\Delta}^\rho_m$. Here, $D^{a,b}_\rho$ is finite for both $a + b = 2$ and $a + b = 3$ as mentioned above.

Next, we consider the GL free-energy $F^{(2)} \propto \sum_\rho A_\rho |\varphi_\rho|^2$. Based on the Luttinger-Ward-Eliashberg formalism, the relation $A_\rho \sim 1 - \lambda_\rho$ is derived in Ref. [22] because $\chi^{0\phi}_{\rm pp}$ in this reference is proportional to λ_ρ. Using this relation, the variation of the coefficient A_ρ is simply given by $\Delta A_\rho \propto -\Delta\lambda_\rho$. Comparing the pair of chiral solutions $\rho = d_+$ and $\rho' = d_-$, which satisfy $\Delta^\rho_m = (\Delta^{\rho'}_m)^*$, the coefficient $\bar{M}_{\rm orb}$ in Supplementary Equation (19) is found to be proportional to $A_{d+} - A_{d-} \propto \Delta\lambda_{d-} - \Delta\lambda_{d+}$. This quantity is given by the imaginary pair hopping term γ_{lm} $(l \neq m)$, namely $\gamma^{a,b}_{lm}$ with $a = 1, 3, \cdots$. By comparison with Supplementary Equation (20), and restricting to $a + b \leq 3$, we obtain the relation $(\bar{m}_1, \bar{m}_2, \bar{m}_3) \propto (\delta D^{1,1}_d, \delta D^{1,2}_d, \delta D^{3,0}_d)$, where $\delta D^{a,b}_d \equiv D^{a,b}_{d-} - D^{a,b}_{d+}$. We also note that, up to third order, $\Delta\lambda_{d-} - \Delta\lambda_{d+} = \delta D^{1,1}_d \eta\phi + \delta D^{1,2}_d \eta\phi^2 + \delta D^{3,0}_d \eta^3$.

iv. *Relation between $M_{\rm orb}$ and chirality of the SC gap*

We further clarify the relation between the winding number w of the d-wave superconducting state and the uniform magnetization $M_{\rm orb}$. Since $M_{\rm orb}$ is given by an integral over the Fermi sea [21], it originates from normal-state electrons when $|\Delta| \ll |\eta|$. Here, we set $\boldsymbol{\eta} = \eta(1, 1, 1)$ and $\boldsymbol{\phi} = \phi(1, 1, 1)$ to express the nematic LC+BO phase.

Supplementary Figure 10 **a** shows the η dependence of $M_{\rm orb}$ per V site at $T = 0.8$meV, which is derived from the exact expression of $M_{\rm orb}$ given in Supplementary Equation (14). Here, $|\eta|$ and $|\phi|$ are taken to be sufficiently small. For $\phi = 0$, $M_{\rm orb}$ is positive and scales as η^3, which arises from the $-m_3\eta^3$ term in Supplementary Equation (18). For $\phi = +0.008$ (-0.008), $M_{\rm orb}$ is positive (negative) and proportional to η. This result comes from the $m_1\eta\phi$ term in Supplementary Equation (18). Thus, $m_1 > 0$ and $m_3 < 0$.

Supplementary Figure 10 **b** shows the η dependence of the splitting in the chiral $d_\pm$ wave superconducting eigenvalues, $\Delta\lambda_d \equiv \lambda_{d-} - \lambda_{d+}$, at $T = 0.8$meV. For $\phi = 0$, the obtained $\Delta\lambda_d$ at $g = 1$ decreases proportionally to η^3. Considering the relation $\bar{M}_{\rm orb} \propto \Delta\lambda_d$ derived in the Supplementary Note H **iii**, this result originates from the $-\bar{m}_3\eta^3$ term in Supplementary Equation (20). For $\phi = +0.008$ (-0.008), $\Delta\lambda_d$ decreases (increases) linearly with η. This result comes from the $\bar{m}_1\eta\phi$ term in Supplementary Equation (20). Thus, $\bar{m}_1 > 0$ and $\bar{m}_3 < 0$.

Therefore, the expression for the LC-SC coupling free energy $F_{\text{OM-ch}}$ given in Supplementary Equations (19) and (20) is numerically validated. In addition, overall proportionality relation $m_n \propto \bar{m}_n$ $(n = 1, 2, 3)$ is obtained, although m_n and $\bar{m}_n$ are not identical mathematically. (Note that $m_2 = -m_3$ and $\bar{m}_2 = -\bar{m}_3$.)

Supplementary Figures 10 **c**-**e** shows the obtained $M_{\mathbf{k}}^{\rm orb}$, which represents the orbital magnetization given by the quasiparticle at $\mathbf{k}$, for the **c** LC phase $(\eta > 0)$, **d** LC + BO(TrH) phase $(\phi > 0)$, and **e** LC + BO(SoD) phase $(\phi < 0)$. We see that $M_{\mathbf{k}}^{\rm orb}$ exhibits a pronounced $\mathbf{k}$-dependence and takes large values near the FS, especially around the Γ-point Fermi pocket. Thus, the LC-induced $M_{\mathbf{k}}^{\rm orb}$ will act on the Cooper pairs around Γ Fermi pocket.

Supplementary Figures 10 **f**-**h** show the phase $\theta_{\mathbf{k}}$ of the chiral SC gap for the **f** LC phase $(\eta > 0)$, **g** LC + BO(TrH) phase $(\phi > 0)$, and **h** LC + BO(SoD) phase $(\phi < 0)$. Here, we set $T = 0.8$meV. The relation $w_1 = -w_2$ is realized for sufficiently small ϕ, η. Thus, the winding number is $w = +2$ in **f** and **g**, while $w = -2$ in **h**. The present result indicates that the orbital magnetization $M_{\mathbf{k}}^{\rm orb}$ for the Γ Fermi pocket determines the chirality of the d-wave SC gap w_1.

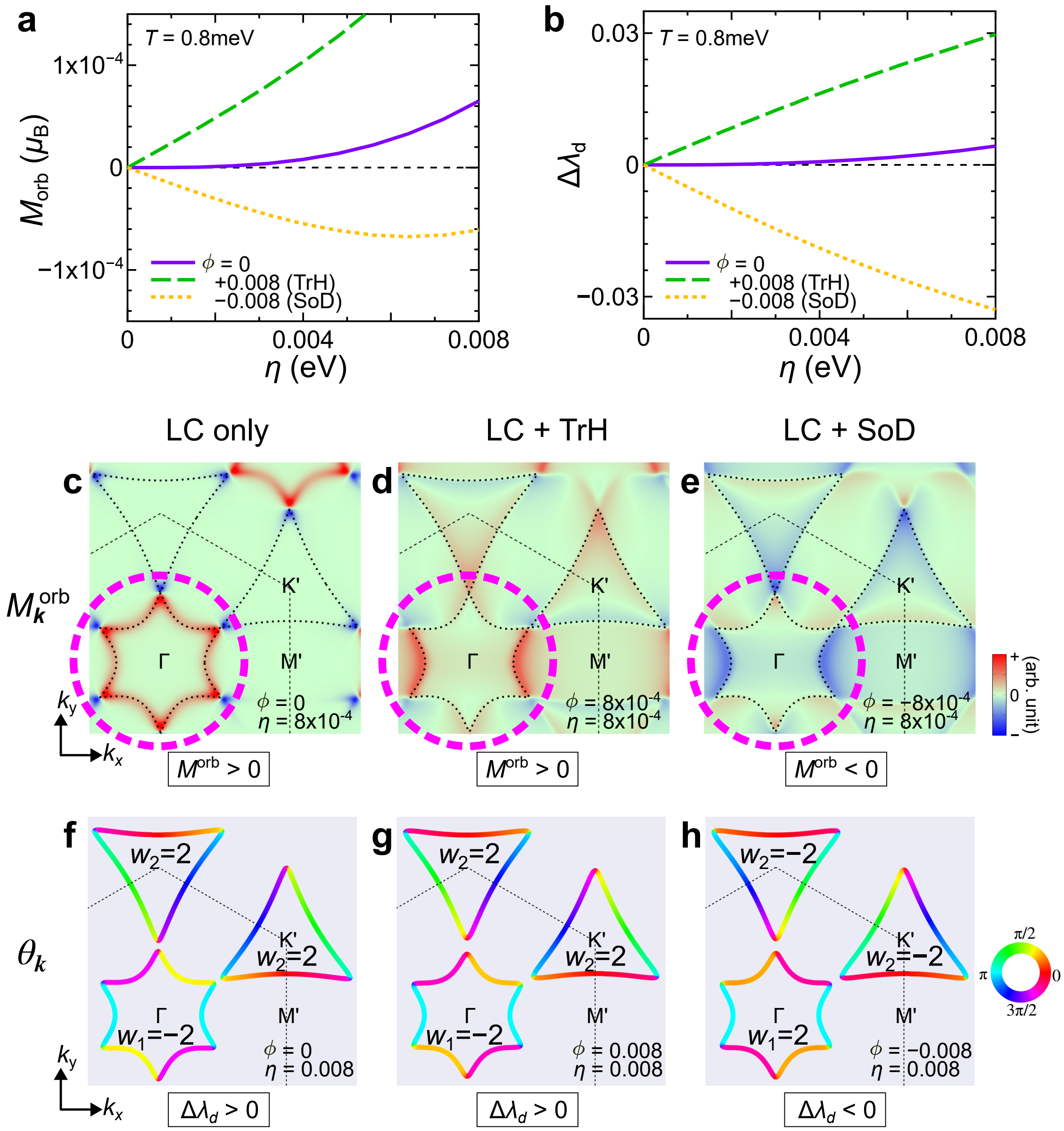

Supplementary Figure 10. **Relationship between $M_{\rm orb}$ and $\Delta\lambda_d$.** **a**,**b** Numerical results for $n = 11.84$: $M_{\rm orb}$ per V site and $\Delta\lambda_d$ at $g = 1$ as functions of η for $\phi = 0, \pm 0.008$. For $\phi = 0$, both quantities are proportional to η^3. For $\phi \neq 0$, both quantities are linear-in-η. Both $M_{\rm orb}$ and $\Delta\lambda_d$ are positive for $\phi = 0$ and $+0.008$, while they are negative for $\phi = -0.008$. **c**-**e** Orbital magnetization at **k**, $M^{\rm orb}_{\mathbf{k}}$, for **c** only LC, **d** LC + TrH, and **e** LC + SoD phases. It is found that $M_{\rm orb}$ is mainly originates from the Γ-point Fermi pocket. (Note that we set $T = 8$meV in **c**-**e** for clearer visualization of the colormap.) **f**-**h** $\theta_{\mathbf{k}}$ for the obtained chiral d-wave SC gap for the **f** only LC, **g** LC + TrH, and **h** LC + SoD phases. The winding number is $w = +2$ in **h** and **i**, while $w = -2$ in **j**.

To summarize, the present numerical results provide clear support for the LC-SC coupling free energy $F_{\text{OM-ch}}$ given in Supplementary Equations (19) and (20). The GL coefficient $\bar{M}_{\text{orb}}$ has a functional form similar to M_{orb}. This OM-chirality coupling mechanism stabilizes chiral superconductivity in the LC phase. From the numerical results in Supplementary Figure 10 **a** and the analytic expression in Supplementary Eq. (18), we estimate $|M_{\text{orb}}| = 10^{-4} - 10^{-3}$ $[\mu_B]$ per V atom for $\phi, \eta = 10 \sim 20$meV [3]. Importantly, this mechanism becomes even more significant deep in the SC phase ($T \ll T_{\text{c}}$); see the Supplementary Note K. The present study constitutes one of the key results of the manuscript.

Supplementary Note I: In-plane anisotropy of H_{c2} in the nematic SC state

Here we discuss the upper critical magnetic field H_{c2} arising from the orbital depairing effect in the clean limit; $H^{\mu}_{c2} = \Phi_0/2\pi\xi_\nu\xi_z$, where $\mu, \nu = x, y$ with $\mu \neq \nu$, ξ_μ is the superconducting coherence length along the μ direction, and $\Phi_0 = h/2e$ is the magnetic flux quantum. The in-plane anisotropy of H_{c2} is therefore expressed as $H^x_{c2}/H^y_{c2} = \xi_x/\xi_y$. Within the BCS theory, the coherence length is given by $\xi^2_\mu \propto \left\langle v^2_{k,\mu}|\Delta_{\mathbf{k}}|^2\right\rangle_{\text{FS}} (\bar{\Delta}T_{\text{c}})^{-2}(1-T/T_{\text{c}})^{-1}$, where $v_{k,\mu}$ is the μ component of the Fermi velocity at momentum $\mathbf{k}$, and $\langle\cdots\rangle_{\text{FS}}$ denotes the Fermi-surface average. Thus, the anisotropy of the upper critical field can be written as

$$\frac{H^x_{c2}}{H^y_{c2}} = \frac{\left\langle v^2_{k,x}|\Delta_{\mathbf{k}}|^2\right\rangle^{1/2}_{\text{FS}}}{\left\langle v^2_{k,y}|\Delta_{\mathbf{k}}|^2\right\rangle^{1/2}_{\text{FS}}}. \tag{21}$$

In kagome metals, the Fermi velocity approximately preserves C_6 symmetry even in the nematic LC+BO phase. In contrast, the superconducting gap amplitude $|\Delta_{\mathbf{k}}|$ exhibits pronounced C_2 symmetry in the nematic chiral superconducting state, as shown in Figs. 4 **b** and **f** of the main text. Therefore, the experimentally observed in-plane anisotropy of H_{c2} reported in Refs. [23, 24] originates from the in-plane anisotropy of $|\Delta_{\mathbf{k}}|$.

In this paper, the y direction corresponds to the nearest-neighbor V–V direction. For the C_2 LC+SoD BO state shown in Fig. 4**b** in the main text, we obtain $\xi_x/\xi_y = H^x_{c2}/H^y_{c2} < 1$, in agreement with Ref. [24] in the main text. In contrast, for the C_2 LC+TrH BO state shown in Fig. 4**f** in the main text will lead to $\xi_x/\xi_y = H^x_{c2}/H^y_{c2} > 1$. More detailed analysis based on a more realistic multiorbital model is equired for a quantitative understanding of the in-plane H_{c2} anisotropy.

Importantly, Ref. [23] reports that $(H^x_{c2} - H^y_{c2})/(H^x_{c2} + H^y_{c2})$ becomes finite just below T_c, indicating that the electronic state is already nematic in the normal state. This observation is fully

consistent with the present theory of a nematic chiral superconducting state emerging from the C_2-symmetric LC+BO phase.

Supplementary Note J: Remarkable nonmagnetic impurity effects on the $s + d^{\pm}$ wave SC states

In the main text, we found that the $s+d^{\pm}$ wave SC state is realized in the nematic LC+BO phase. As shown in Fig. 4 and Fig. 5 in the main text, the d-wave SC component $\bar{\Delta}^d/\bar{\Delta}$ exceeds 50% for a wide parameter range. On the other hand, as is well-known, the impurity dependence differs markedly between the d-wave and s-wave gaps, and the sign-changing d-wave gap is strongly suppressed by impurity scattering. As studied in Ref. [25], in kagome lattice models, the d-wave SC state is suppressed by on-site impurities, although the reduction ratio is significantly smaller than the simple Abrikosov–Gorkov value due to sublattice interference effects.

Therefore, one can intuitively expect that the $s + d^{\pm}$ wave SC state is transformed into a simple s-wave SC state upon the introduction of nonmagnetic impurities. To understand the impurity effect on the SC states in kagome metals, we apply the self-consistent T-matrix approximation. The T-matrix due to the onsite impurity potential at m-sublattice is $T_m(\epsilon_n) = I/(1 - g_m(\epsilon_n)I)$, where I is the impurity potential and $g_m(\epsilon_n)$ is the local Green function. In the T-matrix approximation, the impurity-induced local self-energy is $\Sigma^m(\epsilon_n) = n_{\rm imp}T_m(\epsilon_n)$, where $n_{\rm imp}$ is the impurity concentration. The Dyson equation for the Green function with $n_{\rm imp} \neq 0$ is given by $(\hat{\tilde{G}}_{\mathbf{k}}(\epsilon_n))^{-1} = (\hat{G}_{\mathbf{k}}(\epsilon_n))^{-1} - \hat{\Sigma}(\epsilon_n)$, where $\hat{G}_{\mathbf{k}}(\epsilon_n)$ is the Green function for $n_{\rm imp} = 0$. Within the self-consistent T-matrix approximation, we numerically solve the T-matrix $\tilde{T}_m(\epsilon_n)$ and the local Green function $\tilde{g}_m(\epsilon_n) = \frac{1}{N}\sum_{\mathbf{k}} \tilde{G}^{mm}_{\mathbf{k}}(\epsilon_n)$ in a self-consistent manner. Hereafter, we set $I = 100$ eV to discuss the unitary impurity case.

Next, we solve the following SC gap equation in the presence of impurities [26]:

$$\lambda\Delta_m(\epsilon_n) = gK_m(\epsilon_n) + \Delta_m^{\rm imp}(\epsilon_n), \tag{22}$$

$$K_m(\epsilon_n) = \frac{T}{N}\sum_{\mathbf{k},n',l} \tilde{G}^{ml}_{\mathbf{k}}(\epsilon_{n'})\tilde{G}^{ml}_{-\mathbf{k}}(-\epsilon_{n'}) \times\Delta_l(\epsilon_{n'})\Theta(\epsilon_{n'};\Omega), \tag{23}$$

$$\Delta_m^{\rm imp} = |T_m(\epsilon_n)|^2\frac{n_{\rm imp}}{N}\sum_{\mathbf{k},l} \tilde{G}^{ml}_{\mathbf{k}}(\epsilon_n)\tilde{G}^{ml}_{-\mathbf{k}}(-\epsilon_n) \times\Delta_l(\epsilon_n). \tag{24}$$

For $n_{\rm imp} \neq 0$, the ϵ_n-dependence of the gap function is essential for obtaining physically reliable results.

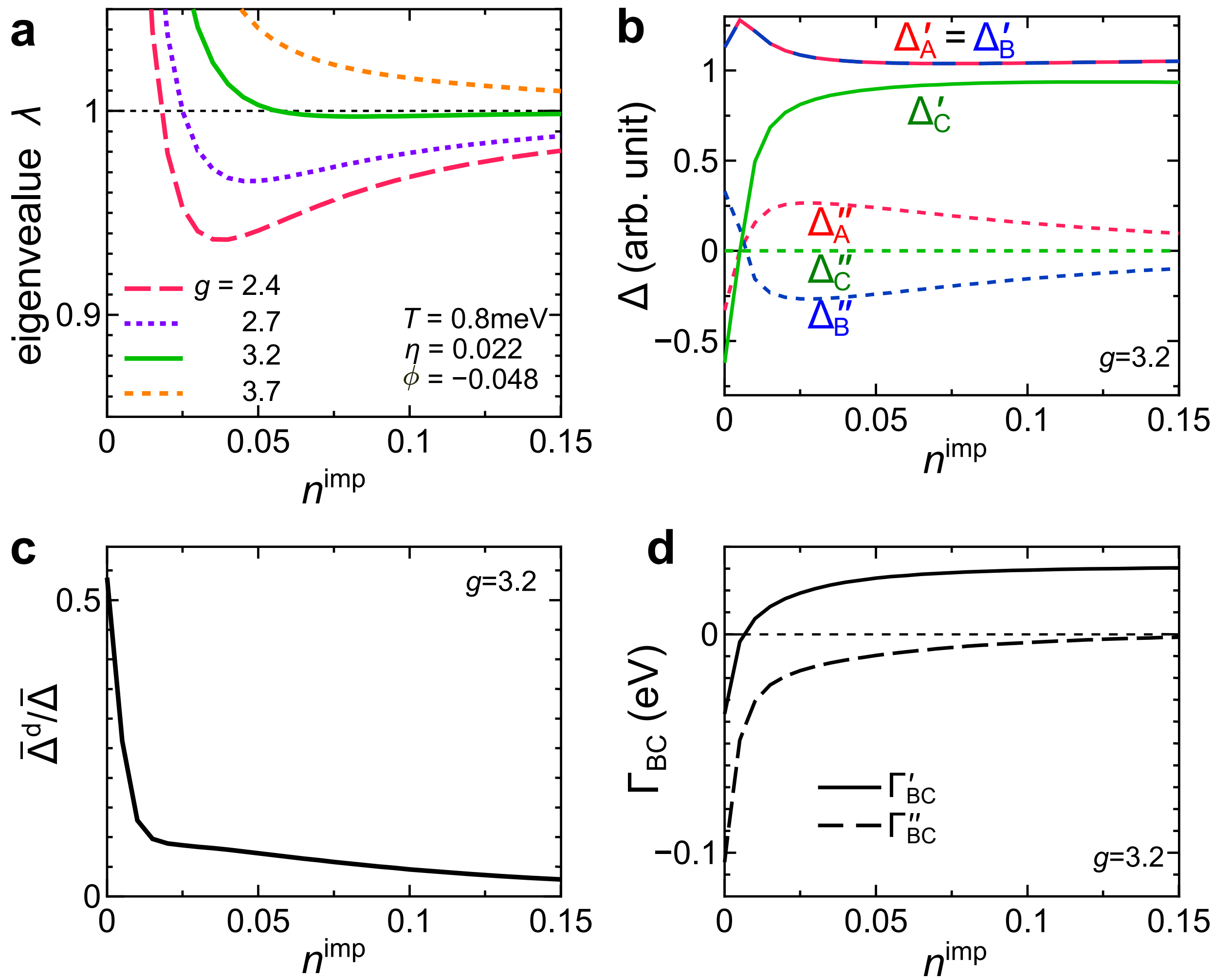

Supplementary Figure 11. **Impurity induced crossover from chiral d-wave gap to s-wave gap in the LC+BO phase.** **a** λ, **b** Δ_m, and **c** $\bar{\Delta}^d/\bar{\Delta}$ as functions of n_{imp} in the case of $\phi = -0.048, \eta = 0.022$ ($T = 0.8$meV). At $n_{\text{imp}} = 0$, the SC gaps is dominated by a significant d-wave component ($\bar{\Delta}^d/\bar{\Delta} \sim 0.6$). For $n_{\text{imp}} \gtrsim 1\%$, however, the SC gaps changed to nearly s-wave state ($\bar{\Delta}^d/\bar{\Delta} \sim 0.1$). In **a**, λ approaches unity with increasing n_{imp} for $g = 2.4 \sim 3.7$. In particular, at $g = 3.2$, the relation $\lambda \approx 1$ (i.e., $T_{\text{c}} \approx 0.8$meV) is realized for $n_{\text{imp}} \gtrsim 3\%$. (Note that T_{c} changes with g.) These behaviors are hallmarks of an impurity-insensitive s-wave state. **d** Complex off-diagonal kernel function $\Gamma'_{\text{BC}} + i\Gamma''_{\text{BC}}$ as a function of n_{imp}.

Here, we study the gap equation in the case of $\phi = -0.048, \eta = 0.022$ at $T = 0.8$meV. Supplementary Figures 11 **a-c** show the obtained eigenvalue of the SC gap equation λ, the SC gap function $\Delta_m(\epsilon_n)$ at $\epsilon_n = \pi T$, and the d-wave SC gap component $\bar{\Delta}^d/\bar{\Delta}$, respectively, as functions of n_{imp}. At $n_{\text{imp}} = 0$, the SC gaps is dominated by a significant d-wave component ($\bar{\Delta}^d/\bar{\Delta} \sim 0.6$). For $n_{\text{imp}} \gtrsim 1\%$, however, the SC gaps changed to nearly s-wave state ($\bar{\Delta}^d/\bar{\Delta} \sim 0$), as demon-

strated in Supplementary Figures 11 **b** and **c**. In Supplementary Figure 11 **a**, λ approaches unity with increasing $n_{\rm imp}$ for $g = 2.4 \sim 3.7$. Especially, at $g = 3.2$, the relation $\lambda \approx 1$ is realized for $n_{\rm imp} \gtrsim 3\%$, indicating that $T_{\rm c} = 0.8$meV is nearly independent of $n_{\rm imp}$. (Note that $T_{\rm c}$ changes with g.) These behaviors are hallmarks of an impurity-insensitive s-wave state. (In contrast, the eigenvalue of non-s-wave SC states decreases monotonically and crosses the horizontal $\lambda = 1$ line as $n_{\rm imp}$ increases.)

Therefore, the Anderson theorem is satisfied in the s-wave SC state in the dirty region $n_{\rm imp} \gg 1\%$. This crossover behavior is reminiscent of the impurity-induced crossover from the $s_\pm$ wave to the s_{++} wave state discussed in Fe-based superconductors [26].

As we explain in the main text, the complex kernel function $\Gamma_{l,m}$ ($l \neq m$) due to the LC-induced phase factor gives rise to the chiral SC state. As shown in Supplementary Figures 11 **d**, its imaginary part $\Gamma''_{\rm B,C}$ is suppressed for $n_{\rm imp} \gg 1$ due to the impurity-induced self-energy. Note that $\Gamma'_{\rm B,C} + i\Gamma''_{\rm B,C}$ correspond to $a - ib$ in Eq.(3) of the main text. As a result, the Anderson theorem holds for the dirty s-wave SC state in the LC phase. Physically, this occurs because the orbital motion of electrons induced by the effective magnetic field generated by the LC is suppressed by impurity scattering.

Supplementary Note K: Analysis of nonlinear gap equation in the LC+BO phase

In the present study, we analyze the linearlized SC gap equation. In the obtained solution, the eigenvalue λ represents the instability of the superconductivity; the relation $\lambda = 1$ holds at $T = T_{\rm c}$. However, the obtained superconducting gap function, given by the linearized superconducting gap equation, neglects nonlinear effects and is therefore justified only in the immediate vicinity of T_c. Therefore, to obtain a reliable gap function for $T \ll T_c$, the following nonlinear superconducting gap equation must be analyzed:

$$\Delta_m(\epsilon_n) = g\frac{T}{N}\sum_{\mathbf{k},n'} F^{mm}_{\mathbf{k}}(\epsilon_{n'};\hat{\Delta})\Theta(\epsilon_{n'};\Omega), \tag{25}$$

where $F^{mm}_{\mathbf{k}}(\epsilon_{n'};\hat{\Delta})$ is the anomalous Green function in the presence of the SC gap $\hat{\Delta}$. $\Theta(\epsilon_{n'};\Omega)$ is a step function that allows Matsubara frequencies with energies below the BCS cutoff energy Ω. Here, we set $\Omega = 0.01$.

First, we solve Supplementary Equation (25) for the LC phase without BO. Supplementary Figure 12 **a** shows the LC-induced chiral d-wave SC gap function for $\phi = 0$, $\eta = 0.032$, and

$g = 1.6$. (When $\eta = 0$, simple s-wave SC gap is obtained.) The chiral d-wave SC gap function is realized for $T \ll T_c$.

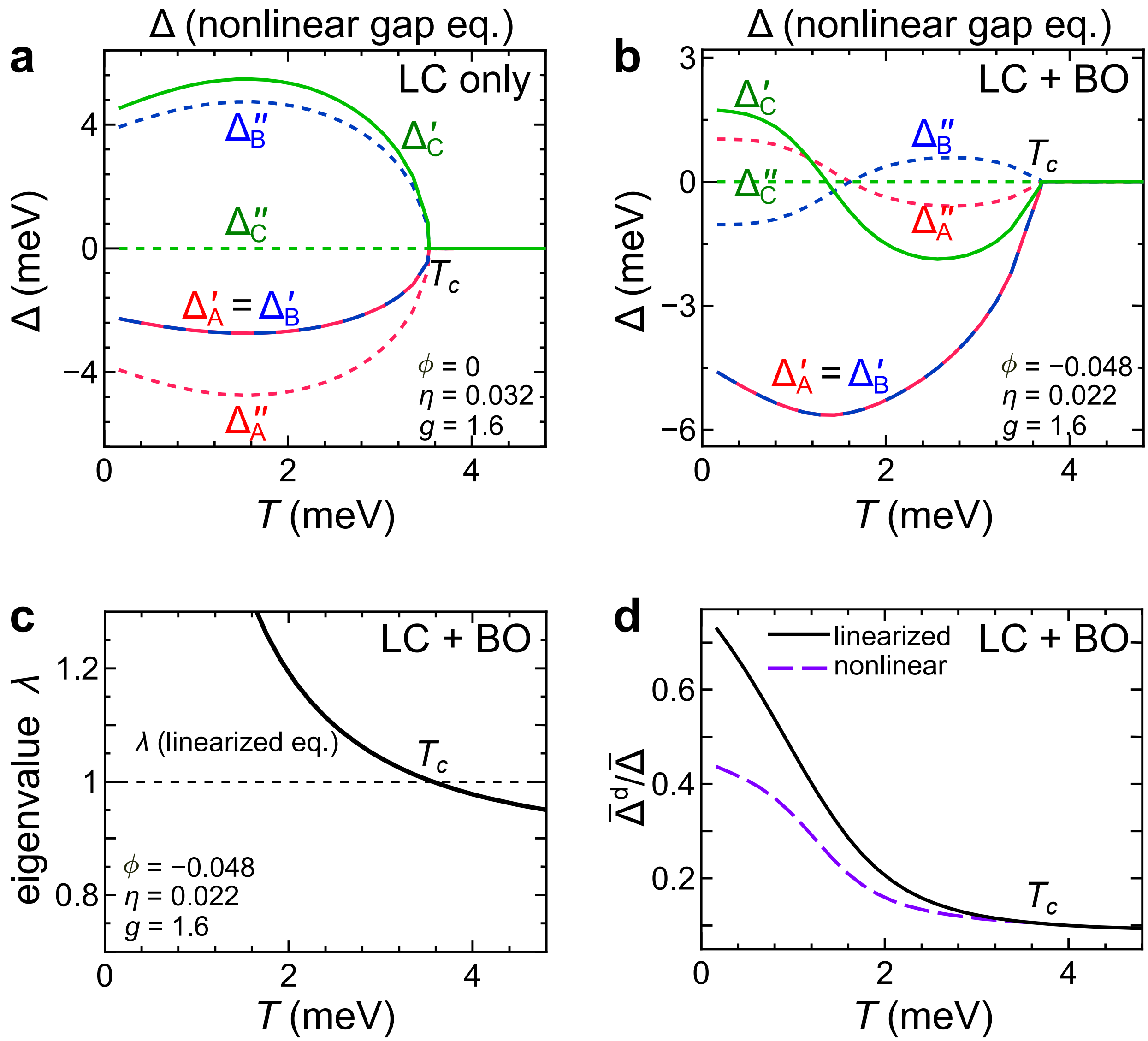

Supplementary Figure 12. **Crossover from s-wave type gap to chiral d-wave gap in the LC+BO phase below T_c.** **a** Chiral d-wave gap in the LC phase below $T_c = 3.5$meV given by the nonlinear gap equation. The parameters are $\phi = 0$, $\eta = 0.022$, $g = 1.6$. **b** s + chiral d-wave state in the LC+BO phase below $T_c = 2.7$meV given by the nonlinear gap equation. The parameters are $\phi = -0.048$, $\eta = 0.022$, $g = 1.6$. **c** Eigenvalue of the linearized gap equation in the LC+BO phase. **d** d-wave SC gap component $\bar{\Delta}^d/\bar{\Delta}$ given by the nonlinear gap equation and linearized one.

Next, we solve Supplementary Equation (25) for the LC+BO phase. Supplementary Figure 12 **b** shows the s + chiral d-wave state for $\phi = -0.048$, $\eta = 0.032$, $g = 1.6$. Interestingly, the

obtained SC gap exhibits nontrivial temperature dependence below T_c, which cannot be achieved by the linearized gap equation analysis. For reference, Supplementary Figure 12 **c** shows the temperature dependence of the eigenvalues of the linearized gap equation. Supplementary Figure 12 **d** exhibits the d-wave SC gap component $\bar{\Delta}^d/\bar{\Delta}$ given by the nonlinear gap equation and linearized one. Interestingly, the d-wave component increases drastically below T_c, owing to the prominent complex kernel function $\Gamma_{l,m}$ at low temperatures, as revealed in the main text.

In summary, a novel and pronounced enhancement of the d-wave SC weight within the s + chiral d-wave SC phase is obtained by solving the nonlinear superconducting gap equation. Importantly, the OM-chirality coupling mechanism for the chiral SC state becomes more pronounced at low temperatures, even for $T \ll T_c$. The present study constitutes one of the key results of the manuscript. We note that, in CsV_3Sb_5, an abrupt change of the SC gap function around $T \sim T_c/3$ has been reported in Ref. [27]. In addition, the thermal Hal effect observed below T_c in kagome metals also increases sharply below $T \sim T_c/3$ [28]. The abrupt change in the SC gap obtained in the present theory may provide a possible explanation for these experimental findings.

---

[1] Tazai, R., Yamakawa, Y., Onari, S. & Kontani, H. Mechanism of exotic density-wave and beyond-Migdal unconventional superconductivity in kagome metal AV_3Sb_5 (A=K, Rb, Cs). *Sci. Adv.* **8**, eabl4108 (2022).

[2] Tazai, R., Yamakawa, Y. & Kontani, H. Charge-loop current order and Z_3 nematicity mediated by bond order fluctuations in kagome metals. *Nat. Commun.* **14**, 7845 (2023).

[3] Tazai, R., Yamakawa, Y. & Kontani, H. Drastic magnetic-field-induced chiral current order and emergent current-bond-field interplay in kagome metals. *Proc. Natl Acad. Sci. USA* **121**, e2303476121 (2024).

[4] Roppongi, M. et al. Bulk evidence of anisotropic s-wave pairing with no sign change in the kagome superconductor CsV_3Sb_5. *Nat. Commun.* **14**, 667 (2023).

[5] Zhang, W. et al. Nodeless superconductivity in kagome metal CsV_3Sb_5 with and without time reversal symmetry breaking. *Nano Lett.* **23**, 872 (2023).

[6] Zhong, Y. et al. Nodeless electron pairing in CsV_3Sb_5-derived kagome superconductors. *Nature* **617**, 488 (2023).

[7] Deng, H. et al. Chiral kagome superconductivity modulations with residual Fermi arcs. *Nature* **632**,

775 (2024).

[8] Yan, X.-Y. et al. Phase-sensitive evidence for 2×2 pair density wave in a kagome superconductor. *Proc. Natl. Acad. Sci. U.S.A.* **123**, e2604142123 (2026).

[9] Song, W. et al. Switchable chiral pair density wave in pure CsV_3Sb_5. *Phys. Rev. B* **113**, 125127 (2026).

[10] Le, T. et al. Superconducting diode effect and interference patterns in kagome CsV_3Sb_5. *Nature* **630**, 64 (2024).

[11] Ge, J. et al. Nonreciprocal superconducting critical currents with normal state field trainability in kagome superconductor CsV_3Sb_5. *Nat. Commun.* (2026), doi:10.1038/s41467-026-72799-6. (Preprint at https://arxiv.org/abs/2506.04601 (2025).)

[12] Wu, X. et al. Nature of Unconventional Pairing in the Kagome Superconductors AV_3Sb_5 (A = K, Rb, Cs). *Phys. Rev. Lett.* **127**, 177001 (2021).

[13] Gao, Y. Robust A_1 superconductivity in the kagome lattice. *Phys. Rev. B* **109**, 214501 (2024).

[14] Guo, C. et al. Switchable chiral transport in charge-ordered kagome metal CsV_3Sb_5. *Nature* **611**, 461 (2022).

[15] Tazai, R., Yamakawa, Y., Morimoto, T. & Kontani, H. Quantum-metric-induced giant and reversible nonreciprocal transport phenomena in chiral loop-current phases of kagome metals. *Proc. Natl Acad. Sci. USA* **122**, e2503645122 (2025).

[16] Schultz, D. J., Palle, G., Mitra, A., Kim, Y. B., Fernandes, R. M. & Schmalian, J. Superconductivity in kagome metals due to soft loop-current fluctuations. *Nat. Commun.* **17**, 4557 (2026).

[17] Zhong, Y. et al. Photoemission Insights to Electronic Orders in Kagome Superconductor AV_3Sb_5. *J. Phys. Soc. Jpn.* **93**, 111001 (2024).

[18] He, G. et al. Anharmonic strong-coupling effects at the origin of the charge density wave in CsV_3Sb_5. *Nat. Commun.* **15**, 1895 (2024).

[19] Gui, H. et al. Probing orbital magnetism of a kagome metal CsV_3Sb_5 by a tuning fork resonator. *Nat. Commun.* **16**, 4275 (2025).

[20] Asaba, T. et al. Evidence for an odd-parity nematic phase above the charge-density-wave transition in a kagome metal. *Nat. Phys.* **20**, 40 (2024).

[21] Shi, J., Vignale, G., Xiao, D. & Niu, Q. Quantum Theory of Orbital Magnetization and Its Generalization to Interacting Systems. *Phys. Rev. Lett.* **99**, 197202 (2007).

[22] Tazai, R., Matsubara, S., Yamakawa, Y., Onari, S. & Kontani, H. Rigorous formalism for unconventional symmetry breaking in Fermi liquid theory and its application to nematicity in FeSe. *Phys. Rev.*

B **107**, 035137 (2023).

[23] Fukushima, K. et al. Violation of emergent rotational symmetry in the hexagonal Kagome superconductor CsV_3Sb_5. *Nat. Commun.* **15**, 2888 (2024).

[24] Xiang, Y. et al. Twofold symmetry of c-axis resistivity in topological kagome superconductor CsV_3Sb_5 with in-plane rotating magnetic field. *Nat. Commun.* **12**, 6727 (2021).

[25] Holbæk, S. C., Christensen, M. H., Kreisel, A. & Andersen, B. M. Unconventional superconductivity protected from disorder on the kagome lattice. *Phys. Rev. B* **108**, 144508 (2023).

[26] Onari, S. & Kontani, H. Violation of Anderson's Theorem for Sign-Reversing s-Wave State of Iron-Pnictide Superconductors. *Phys. Rev. Lett.* **103**, 177001 (2009).

[27] Hossain, M. S. et al. Unconventional gapping behaviour in a kagome superconductor. *Nat. Phys.* **21**, 556 (2025).

[28] Yoshida, H. et al. Observation of Anomalous Thermal Hall Effect in a Kagome Superconductor. *Sci. Adv.* **11**, eadu2973 (2025).
