# Supplementary material for: Nematic and chiral superconductivity emerging within the loop-current phase in kagome metals
Source: Nat Commun. 2026 Jul 27;17:9114. doi: 10.1038/s41467-026-75259-3 (PMC13518961; doi:10.1038/s41467-026-75259-3)
Supplement: Supplementary file 3 [file 41467_2026_75259_MOESM3_ESM.pdf]

nature portfolio

Peer Review File

Nematic and chiral superconductivity emerging within the loop-current phase in kagome metals

Corresponding Author: Professor Hiroshi Kontani

This file contains all reviewer reports in order by version, followed by all author rebuttals in order by version.

Version 0:

Reviewer comments:

Reviewer #1

(Remarks to the Author)
The authors present a microscopic superconducting theory based on the kagome lattice, incorporating loop-current and bond-order charge density wave, aiming to address several key issues in kagome SC. These include: 1) time-reversal symmetry breaking chiral SC states, 2) nematic SC gap anisotropy, 3) a 2×2 pair density wave and 4) the emergence of an s-wave superconducting state under disorder. The pairing interactions is mediated by fluctuations in the charge channel, which also give rise to the complex bond order. The origin of the complex d-wave SC states is attributed to the complex Josephson coupling in the off-diagonal entries of the kernel functions, as explained through the leading order expansion of the off-diagonal scatterings. In the presence of the LC+BO, a nematic chiral SC with significant nematic gap anisotropy is obtained.

This study represents a timely contribution to the field of kagome metals and superconductors and attempts to resolve several key questions regarding the superconducting mechanisms. The article is well-written and the structure is clear. However, the there are some major concerns that need to be clarified before the paper can be considered for publication on Nature Communications. My concerns are listed in the following.

1) The authors state that the pairing interaction arises from the attractive charge-channel interactions which are also held to be responsible for the LC order (line 69). It would be helpful to clarify the different energy scales or critical temperatures associated with the two (SC, LC) orderings observed in experiments.

2) The V-based kagome metals maintain a complex atomic orbital content. Although a single-orbital model can be illustrative in addressing the symmetry breaking features, could the authors further justify the use of the simplified model?

3) Could the authors justify the filling choice n=11 based on experimental observations? In particular, as the filling is away from the van Hove singularity, one would expect a much weaker CDW instability. The authors should first provide evidence of the CDW order normal state obtained under such a filling.

4) The TRS breaking feature due to the LC order in the normal state can lead to non-trivial topological properties, which can be characterized by a non-zero Chern number. In this work, the obtained chiral d-wave SC state is also claimed to be topologically non-trivial. Could the authors elaborate on the connections between the normal state and the SC state in terms of topology? Is the non-trivial topology of the chiral d-wave SC an intrinsic SC property, or does it simply stem from an ordinary pairing in a doped Chern insulator? In addition, is the s-wave state obtained in the presence of LC order also topologically non-trivial?

5) Is there any sign of TRS breaking in the obtained s-wave superconducting state under a pure LC phase? If not, how to understand the complete “screening” of the LC in the s-wave state? A related question: does the pairing angle also possess some small variations along the FS?

6) The authors mentioned prominent Berry curvature around line 91, but its relation to the emergence of the chiral d-wave state remains unclear. In particular, chiral SC states has been obtained on the kagome lattice from complex CDW Hamiltonians [arXiv: 2408.03056, 2504.02751]. The authors should provide more understanding regarding whether the chiral d-wave is favored due to a large Berry curvature or whether there is connection between the two. A related question:

how does the winding of the chiral d-wave order parameter along the FS relate to the Berry phase of the parent state?

7) The Gamma-pocket appears essential in generating the chiral d-wave state and a pronounced 2×2 SC pattern. Do the Fermi surfaces in real materials exhibit similar dispersions?

8) In the analytical analysis of the pair-hopping mechanism depicted in Fig. 3(c), the imaginary part of the off-diagonal kernel function depends on the both BO (ϕ) and the LC (η) orders to an odd power. This implies that changing the BO from a SoD to a tri-hexagonal pattern may yield a different chirality of the SC state. It is surprising that a TRS breaking pattern is influenced by a TRS symmetric term. Could the authors provide further explanations for this?

10) The superconducting state, in spite of various interesting features such as nematicity and TRS breaking, doesn't appear to break additional symmetries beyond those already broken by the parent BO+LC state. In particular, it doesn't break additional translation symmetry beyond the CDW and is therefore a pair density modulation inside the CDW unit cell. Referring to it as a pair density wave would be confusing as it implied translation symmetry breaking. Could the authors clarify whether there are connections to a translation symmetry breaking 4a0 and 4a0/3 PDW as observed in experiments?

11) The Fermi surface shown in Fig. 4 seems to follow C6 symmetry, while the normal state is already nematic in the presence of BO and LC. Is it because the nematicity is relatively weak? The scale of the pairing gap is plotted from 0 to 1, which should be understood as the relative gap value. In that sense, the authors should further estimate the true gap value instead of its relative amplitude.

12) The superconducting solution is proposed to be nematic in the presence of bond order and loop current. Is the nematic direction of the normal state the same as that in the SC state? Would the result be consistent with the experimental observations in, e.g. Nat. Comm. 12, 6727?

13) It would be helpful if the authors could comment on the SC state on the hole-doped side with respect to the van Hove singularity. As one would expect a different dispersion in the hole-doped region, would the chiral d-wave remain favored?

Reviewer #2

(Remarks to the Author)
The paper entitled Nematic chiral Superconductivity driven by chiral loop current order in kagome metals by Rina Tazai, Youchi Yamakawa, Hiroshi Kotani sets out to explain recent experimental results reporting superconducting states in the of the Kagome metals (AV_3Sb_5) with TRSB, nematicity, PDW modulation, and a transition to s-wave SC in the presence of disorder.

The authors consider a simple tight-binding model for the Kagome lattice with hoppings up to next-nearest-neighbours. The authors "assume" that the system has developed loop-current (LC) or charge-bond order (BO), placing in "by-hand" the (possibly) complex modulations of the hopping parameters.

The authors examine the behaviour of the linearized gap equation in the presence of loop-current (LC) order. In the absence of LC order, the s-wave state is selected. In the presence of LC order, a chiral d-wave state is selected. The authors use a 12-site superlattice corresponding to the 2x2 CDW order. The d-wave state is shown to display a nonzero (~5%) dependence within the supercell sublattice, i.e. a PDW.

The authors provide some explanation for this behaviour by presenting a phenomenological kernel function with only three parameters based on their numeric results of the full kernel. The phenomenological kernel is effective in explaining which SC state is selected, assuming the numeric results. However, the attempt to explain the mechanism underlying the numerics themselves (i.e. Fig. 3a-d) may be difficult to follow for readers unfamiliar with the formalism.

The authors cite their previous work [Nat. Comm. 14, 7845 (2023)] to invoke nematicity. According to that paper, nematic order develops when the centres of symmetry of the CO and LC orders are out of phase. The authors then study the behaviour of the gap equation in the presence of coexisting CO and LC orders, finding that the SC gap is also nematic, even though the Fermi surface retains its nearly-perfect C6 symmetry. The selected superconducting state is s + chiral d-wave. The authors invoke Anderson's theorem to explain the suppression of the chiral component in the presence of non-magnetic impurities, but do not support this claim quantitatively.

I have following questions:

1. The tight-binding model used is very simple, and assumes filling at the p-type vHS. Real Kagome metals have multiple vHS near the FS. How do the authors justify the use of this model compared to a more realistic tight-binding model based on DFT (e.g. arXiv:2507.16892)? Does the mechanism rely on a p-type vHS?

2. The amplitudes \phi and \eta for the bond orders are put in by hand, and tuned freely. How do the authors justify their choices of these amplitudes in comparison with experiment or self-consistent theoretical calculations? How does it affect the constant attractive interaction "g", as the attractive interaction is due to BO fluctuations?

3. While it was claimed that the proposed mechanism qualitatively reproduces a number of observed features of the SC state (TRSB, nematicity, PDW, disorder), the connection to experiment is largely descriptive. Can the authors offer more specific connections to experiment?

4. The authors assume a constant attractive interaction. How do the authors expect their results to change with a more sophisticated treatment of the pairing interaction like momentum dependence, etc?

5. Nematicity is introduced by the coexistence of LC and CB order, as was previously argued by the authors in Nat. Comm. 14, 7845 (2023). It is not clear to me what is novel in this work compared to their previous publications (Refs. 10, 37, and 38), aside from introducing the ad hoc constant g and studying the pairing instabilities. Since SC, LC, and CB are all broken-symmetry states, should they be treated on equal footing?

Minor points (mainly typos):
a. Change “sensitively” to“sensitivity” in abstract
b. Change “disorders” to “disorder” in abstract
c. Change “holded" to “folded” on line 173
d. Lines 243 and 244 are repeated on lines 254 and 255.
f. References [4] and [8] are repeated.

In summary, I do not recommend the publication unless the above points are addressed in a satisfactory manner.

Reviewer #3

(Remarks to the Author)
In the manuscript, the authors seek to combine the reported experimental evidence for kagome metals into a consistent formation mechanism of superconductivity. The chosen key aspects from the authors' opinion are time-reversal symmetry breaking, nematicity, and a propensity for pair density wave formation. This phenomenological ansatz adopted by the authors is not obvious. For instance, it is rather a subtle point to assess the current experimental evidence as to whether time reversal symmetry is broken in the kagome superconductors or not: while muSR supports this claim, Kerr spectroscopy discards this possibility. The same holds for pair density wave signatures: it is not clear whether there are just electrons proximized by a charge order which when suggests to be a pair density wave. Only nematicity is largely undisputed uniformly shown by different experimental techniques.

Starting from their phenomenological assumptions, the authors employ sophisticated mean field ansätze to follow up on their suggestion of an s+(d+id) superconducting state, which is the central new proposal in the manuscript in comparison to the current states quo. This finding must be taken with some reservation, as there are so many competing orders involved that any mean field ansatz might fall for its systematic bias.

In total, with regard to publication in Nature Communications, I take a rather neutral stand, slightly leaning against publication in Nature Communications. I simply cannot assign too much relevance to mean field proposals that, in particular in the case of a system as complicated as kagome metals, involve a cascade of mean fields (nematicitiy at higher energy, orbital currents at intermediate energy, superconductivity at low energy. I do, however, acknowledge that the authors' suggestion of s + (d+id) has not yet appeared in the literature.

Two minor points:

1. The manuscript needs a detailed spellcheck. Example: Fermi -> Fermi

2. There is highly related work on the microscopic formation of orbital currents in kagome medals, which the authors might want to consider: https://arxiv.org/pdf/2506.01648, https://arxiv.org/abs/2405.09451.

Version 1:

Reviewer comments:

Reviewer #1

(Remarks to the Author)
The authors have addressed most of my previous questions and concerns satisfactorily. I find that the manuscript has improved significantly, both in terms of technical content and physical clarity. In particular, the introduction of the orbital-magnetization–chirality coupling mechanism provides a more transparent understanding of how loop-current order selects the chiral superconducting state.

With the following points clarified, I would be supportive of publication in Nature Communications.

1. Energy scale of the OM–chirality coupling

One of the key advances in the revision is the identification of the orbital magnetic moment as the driving force for the

splitting between different chiral superconducting channels. I find this mechanism very interesting.

It would be helpful if the authors could provide an estimate of the magnitude of this splitting, for example by comparing the chiral splitting (e.g., \lambda_{d-}-\lambda_{d+}) to the overall pairing strength or to T_c. This would give a clearer sense of how dominant this mechanism is relative to the underlying pairing interaction.

In addition, it would be useful to further clarify the relation between orbital magnetization and Berry curvature in this context, i.e., whether the orbital magnetization should be viewed as the primary driver of chirality selection, or as a phenomenological manifestation of the underlying Berry-phase structure.

2. Role of fluctuations and consistency of the mechanism

In the revised manuscript, the loop-current and bond-order states are assumed to form at temperatures significantly higher than the superconducting transition and to be pinned by the lattice.

Given this, I would appreciate some clarification on the role of fluctuations in the superconducting pairing mechanism. In particular, what type of fluctuations are implicitly involved in generating the effective pairing kernel in this low-temperature regime, and how this is consistent with the assumed rigidity of the loop-current and bond-order background.

3. Topological characterization of the superconducting state

In Reply 1-6, the authors discuss the topology in terms of the winding number of the superconducting gap function. While this is a useful diagnostic, it appears somewhat different from the standard definition of topology in superconductors based on the BdG Chern number.

It would be helpful if the authors could clarify the relation between these two characterizations, and under what conditions the winding number they compute is equivalent to the BdG Chern number. A brief discussion would be sufficient.

Version 2:

Reviewer comments:

Reviewer #1

(Remarks to the Author)
The authors have addressed my remaining questions satisfactorily. The revised manuscript is significantly improved in clarity and technical content, especially through the added discussion of the orbital-magnetization–chirality coupling mechanism.

I find that the main concerns raised in the review process have now been adequately addressed. I support publication in Nature Communications.

Open Access This Peer Review File is licensed under a Creative Commons Attribution 4.0 International License, which permits use, sharing, adaptation, distribution and reproduction in any medium or format, as long as you give appropriate credit to the original author(s) and the source, provide a link to the Creative Commons license, and indicate if changes were made.
In cases where reviewers are anonymous, credit should be given to 'Anonymous Referee' and the source.
The images or other third party material in this Peer Review File are included in the article’s Creative Commons license, unless indicated otherwise in a credit line to the material. If material is not included in the article’s Creative Commons license and your intended use is not permitted by statutory regulation or exceeds the permitted use, you will need to obtain permission directly from the copyright holder.
To view a copy of this license, visit https://creativecommons.org/licenses/by/4.0/

Reply to the Comments by Reviewer #1

○ **Comment 1-1**

The authors present a microscopic superconducting theory based on the kagome lattice, incorporating loop-current and bond-order charge density wave, aiming to address several key issues in kagome SC. These include: 1) time-reversal symmetry breaking chiral SC states, 2) nematic SC gap anisotropy, 3) a 2×2 pair density wave and 4) the emergence of an s-wave superconducting state under disorder. The pairing interactions is mediated by fluctuations in the charge channel, which also give rise to the complex bond order. The origin of the complex d-wave SC states is attributed to the complex Josephson coupling in the off-diagonal entries of the kernel functions, as explained through the leading order expansion of the off-diagonal scatterings. In the presence of the LC+BO, a nematic chiral SC with significant nematic gap anisotropy is obtained.

○ **Reply 1-1**

We sincerely thank Reviewer #1 for accurately and clearly summarizing the main findings of our manuscript. As the reviewer appropriately summarized, the central contributions of our manuscript lie in the development and analysis of a microscopic theory of superconductivity on the kagome lattice, including the roles of loop-current (LC) and bond-order (BO) charge-density waves, the emergence of chiral and nematic superconducting (SC) states, and disorder-induced *s*-wave pairing.

○ **Comment 1-2**

This study represents a timely contribution to the field of kagome metals and superconductors and attempts to resolve several key questions regarding the superconducting mechanisms. The article is well-written and the structure is clear. However, the there are some major concerns that need to be clarified before the paper can be considered for publication on Nature Communications. My concerns are listed in the following.

○ **Reply 1-2**

We thank the reviewer for the positive assessment of the timeliness and relevance of our study to the field of kagome metals and superconductors, as well as for the constructive comments and important concerns raised.

In response to these comments, we have performed substantial additional analyses and calculations to clarify the issues identified. We believe that these new results significantly strengthen the manuscript and improve its overall clarity and presentation.

Below, we provide detailed responses to each comment and explain how the manuscript has been revised accordingly.

○ Comment 1-3

1) The authors state that the pairing interaction arises from the attractive charge-channel interactions which are also held to be responsible for the LC order (line 69). It would be helpful to clarify the different energy scales or critical temperatures associated with the two (SC, LC) orderings observed in experiments.

○ Reply 1-3

We thank the reviewer for this important and constructive suggestion.

Regarding the energy scales associated with the SC, LC, and BO orders, several experimental studies, including angle-resolved photoemission spectroscopy (ARPES) measurements, have provided relevant insights into the magnitudes of these order parameters. Although this issue remains not fully resolved, we summarize the available experimental results below and discuss the corresponding energy scales of the order parameters adopted in the present model.

【Energy scales of the BO and LC】

In the folded Brillouin zone corresponding to the 2×2 BO state, the three van Hove singularity (vHS) points at the M points ($E_{\rm vHS}\sim -0.1$ eV) are folded onto the Γ point, where their degeneracy is lifted by the BO order ϕ, yielding energies $E_{\rm vHS}-2\phi$ (twofold degenerate) and $E_{\rm vHS}+4\phi$ (nondegenerate); see **Fig.S1 c-f** in the Supplementary Information A (**SI A**). According to ARPES experiments, several bands near the vHS are shifted and/or reconstructed on an energy scale of approximately 80 meV below the BO transition temperature $T_{\rm BO}$ (~100K). In the present study, we take

$\phi \sim 30\,\text{meV}$, considering that 2ϕ, 4ϕ correspond to the experimental band-energy reconstruction scale [Y. Zhong *et al.*, JPSJ **93**, 111001 (2024); G. He *et al.*, Nat. Commun. **15**, 1895 (2024); Y.-X. Jiang *et al.*, Nat. Mater. **20**, 1353 (2021); Z. Liang *et al.*, Phys. Rev. X **11**, 031026 (2021); Z. Wang *et al.*, Phys. Rev. B **104**, 075148]. At this point, the ratio $2\phi/T_{\rm BO} \sim 6$, which corresponds to the value in the intermediate-coupling BCS theory.

The magnitude of the LC order parameter remains uncertain. However, within a mean-field-type approximation, it is reasonable to expect $\eta \sim (T_{\rm LC}/T_{\rm BO})\,\phi$. The precise transition temperature $T_{\rm LC}$ also remains an open issue. Various experiments, including switchable nonreciprocal transport (electrical magneto-chiral anisotropy) measurements [C. Guo et al., Nature 611, 461 (2022)] and tunable resonator experiments [H. Gui et al., Nat. Commun. 16, 4275 (2025)], report signatures of broken time-reversal symmetry below $T^* \sim 35$ K, suggesting $T_{\rm LC} \sim 35$ K. The magnetic-field history dependence of the superconducting diode effect (SDE) reported in Ref. [G. Ge et al., arXiv:2506.04601 (2025)] also suggests $T_{\rm LC} \sim 30$ K. On the other hand, μSR measurements have reported higher transition temperatures $T_{\rm LC} \sim 50$–60 K [C. Mielke et al., Nature 602, 245 (2022)]. Interestingly, magnetic torque measurements have identified a single-q LC order that preserves global time-reversal symmetry at $T_{\rm LC}^{\text{single-q}} \sim 120$ K [T. Asaba et al., Nat. Phys. 20, 40 (2024)].

Regarding the SC gap, STM and ARPES experiments report $\Delta \sim 0.5\,\text{meV}$ ($\gtrsim T_{\rm SC}$). Taken together, these observations establish the following hierarchy of energy scales in kagome metals: $\phi \sim 30\,\text{meV}$, $\eta \lesssim \phi$, and $\Delta \ll \phi$. In the present study, we adopt this hierarchy of energy scales. Given the separation of energy scales, the influence of Δ on η and ϕ below $T_{\rm SC}$ is expected to be negligible. Accordingly, they can be treated as constant mean-field parameters in the gap equation.

Action:

To clarify the above important discussion, we have added **Supplementary Information (SI) F**, "**Hierarchy of Energy Scales in Kagome Metals**" (on page14 in the SI).

i. *Emergence of the s wave SC and d wave SC instabilities derived from the parquet RG*,

ii. *Magnitude of the bond order ϕ*,

iii. *Magnitude of the LC order η,*

iv. *Magnitude of the superconducting gap.*

○ Comment 1-4

2) The V-based kagome metals maintain a complex atomic orbital content. Although a single-orbital model can be illustrative in addressing the symmetry breaking features, could the authors further justify the use of the simplified model?

○ Reply 1-4

As the reviewer correctly points out, the Fermi surface of kagome metals has a pronounced multiorbital character. To assess the role of multiorbital effects, in the revised manuscript, we introduce a realistic "**three-dimensional (3D) $xz + yz$ orbital model**", which is an improved version of the model in Ref. [R. Tazai et al., PNAS 122, e2503645122 (2025)]. Additional extensive numerical analyses based on this realistic model have been included in **SI E**. As shown in **Fig. S7**, the chiral SC state emerges over a broad parameter space in η and filling n, which is even wider than that obtained in the 2D xz-orbital model due to the inter-orbital hybridization. Therefore, the theoretical analysis based on the 2D xz-orbital model presented in the main text successfully captures the essential physics.

Thus, the newly added **SI E** not only further supports the robustness of the **LC-induced chiral SC mechanism**, but also provides a minimal yet realistic 3D $xz + yz$ orbital model that will serve as a useful platform for future theoretical investigations.

Note that, in **Fig. S7**, the interaction g and the LC order parameter η are introduced only on the xz orbitals. However, we have confirmed that introducing g and η also on the yz orbitals yields essentially the same results due to the smaller yz-orbital density-of-states (DOS) at E_F. Notably, recent magnetic circular dichroism ARPES experiments have observed broken time-reversal symmetry predominantly originating from the **xz-orbital vHS** [H. J. Elmers et al., PRL 134, 096401 (2025); H. J. Elmers et al., arXiv:2511.01238 (2025)].

In the following, we summarize the essential ingredients of the present three-

dimensional $xz + yz$ orbital kagome metal model.

【Decoupling and relevance of the $xz + yz$ orbitals in kagome metals】

Here, we briefly summarize the multiorbital electronic structure of kagome metals AV_3Sb_5. **Figure R1** in this Reply shows the DOS of the V $3d$ electrons near the Fermi energy E_F in AV_3Sb_5. The DOS associated with the $xz + yz$ orbitals is significantly larger than that of the $xy + (x^2 - y^2)$ and $(3z^2 - r^2)$ orbitals, indicating the emergence of strong electron correlations in the $xz + yz$ sector. Notably, in the two-dimensional limit, the $xz + yz$ orbitals do not hybridize with the other d-orbitals and are therefore decoupled from the latter. For this reason, the $xz + yz$ orbital model provides a particularly suitable minimal description of kagome metals.

【Essence ingredients of the $xz + yz$ orbital model】

The resulting three-dimensional band structures of the present $xz + yz$ orbital model are shown in **Figs. S5** and **S6**: The band dispersions and Fermi surfaces near E_F, which are crucial for superconducting properties, are faithfully reproduced, by setting $t = -0.8$eV and $t' = 0.16\,t$.

The xz-orbital band: This band exhibits a large DOS near the Fermi level E_F, approximately twice that of the yz-orbital band. Importantly, the vHS energy is almost k_z independent, and it lies close to the Fermi level, $E_{vHS}^{xz} - E_F = -0.05 \sim -0.1$eV. Furthermore, each vHS points is composed of a single sublattice (A, B, or C), corresponding to a "sublattice–pure type". These features indicate that strong electron correlations predominantly develop in the xz-orbital band, which is therefore expected to play a central role in the emergence of BO, LC, and superconductivity.

The yz-orbital band: This band has a relatively small DOS near E_F and exhibits pronounced k_z dispersion across the Fermi level: As shown in **Fig. S5(b)**, the van Hove singularity (vHS) energy satisfies $E_{vHS}^{yz} - E_F \sim +0.1$ eV at $k_z = 0$ and $E_{vHS}^{yz} - E_F \sim -0.2$ eV at $k_z = \pi$, highlighting the importance of analyzing the full three-dimensional model. Furthermore, each vHS point is composed of multiple sublattices, i.e., the "sublattice–mix type". These properties suggest that electron correlations in the yz-

orbital band are comparatively weaker.

Inter-orbital mixture: In kagome metal models, large portions of the band contours are gapped out at $E = E_{\mathrm{Dirac}}$ due to the substantial inter-orbital mixing (see **Fig. S6c**). Despite this, strong electron correlation develops in the xz orbital, leading to the BO solution in the density-wave (DW) equation analysis (see **Figs. R2** in this Reply) and the LC -induced chiral SC states (see **Figs. S7**), as we will discuss in detail below.

Action:

To clarify the above discussion, we have added **SI E**, "**chiral d-wave superconductivity in three-dimensional two-orbital kagome metal model**" (on page10 in the SI).

This section includes three important figures:

・**FIG. S5**. **Two-orbital 3D kagome lattice model parameters**; the characteristic band dispersion near E_F is properly reproduced.

・**FIG. S6**. **Fermi surfaces of 3D kagome lattice model**; the FS and inter-orbital band reconstruction at $E=E_{\mathrm{Dirac}}$ are properly reproduced.

・**FIG. S7. LC-induced chiral d-wave SC states in 3D kagome lattice model**; the chiral SC state is robustly obtained.

〇 **Comment 1-5**

3) Could the authors justify the filling choice n=11 based on experimental observations? In particular, as the filling is away from the van Hove singularity, one would expect a much weaker CDW instability. The authors should first provide evidence of the CDW order normal state obtained under such a filling.

〇 **Reply 1-5**

Thank you very much for this important question. By setting t=-0.8 and t′/t=0.16 in the revised manuscript, the filling has been revised to n = 11.84.

As shown **Figs. S5** and **S6**, the present system is away from exact vHS filling ($E_{\mathrm{vHS}}^{xz} - E_{\mathrm{F}} \sim -0.1\mathrm{eV}$). To examine the effect of this deviation, we extended our previous analysis of the BO formation based on the DW equation in Ref. [R. Tazai et al, Sci. Adv. 8, eabl4108 (2022)] by introducing a more realistic three-dimensional multiorbital model. It is

confirmed that a nearly commensurate BO is still obtained even at $E^{xz}_{\mathrm{vHS}} - E_{\mathrm{F}} \sim -0.08$eV. Below, we provide a detailed explanation of this result.

【DW equation analysis for three-dimensional Hubbard model】

Motivated by the comment by Reviewer 1, here we study the CDW instability by analyzing the three-dimensional 30-orbital Hubbard model introduced in Ref. [S. Onari at al., arXiv:2408.11337]. Because the computational cost of this analysis is extremely large, we consider the interaction U only on the xz orbital. In **Figs. R2 (upper panels)** in this Reply, we show the obtained DW equation eigenvalue for the total electron filling n = 31.0 ~ 31.2, which corresponds to $E^{xz}_{\mathrm{vHS}} - E_{\mathrm{F}}$ = −0.05eV ~ −0.08eV. The band dispersions for n=31.0 and 31.2 are shown in **Fig. R2 (lower panels)** in this Reply: The eigenvalue of the DW equation, $\lambda_{\mathbf{q}}$, reaches unity, and it decreases as the temperature increases.

We further discuss the effect of the electron-phonon interaction, which enlarges the total CDW eigenvalue as $\lambda^{tot}_{\mathbf{q}} = \lambda_{\mathbf{q}} + \gamma_{\mathbf{q}}$, where $\gamma_{\mathbf{q}}(> 0)$ originates from the electron–phonon interaction, as discussed for Fe-based superconductors [H. Kontani et al., Adv. Phys. 70, 355 (2021)]. Therefore, even when $\lambda_{\mathbf{q}}$ is slightly incommensurate (for $n = 31.2$), $\lambda^{tot}_{\mathbf{q}}$can become commensurate due to the $\mathbf{q}$-dependence of $\gamma_{\mathbf{q}}$. Furthermore, the triple-$\mathbf{q}$ CDW transition in kagome metals is weak first order due to the "third-order term" in the Ginzburg–Landau free energy [R. Tazai et al, Sci. Adv. 8, eabl4108 (2022)]. As a result, even if $\lambda_{\mathbf{q}}$ is slightly incommensurate, 2×2 CDW may emerge through a first-order transition driven by lattice deformation.

It is natural to expect that the 2×2 CDW observed in kagome metals arises from the cooperative effects of Coulomb repulsion and electron–phonon interactions.

Action:

We show Figure.R1, "DW equation analysis for 3D 30 orbital model"**;** This figure has recently been added to Ref. [S. Onari et al., arXiv:2408.11337; to be published in JPSJ].

○ Comment 1-6

4) The TRS breaking feature due to the LC order in the normal state can lead to non-

trivial topological properties, which can be characterized by a non-zero Chern number. In this work, the obtained chiral d-wave SC state is also claimed to be topologically non-trivial. Could the authors elaborate on the connections between the normal state and the SC state in terms of topology? Is the non-trivial topology of the chiral d-wave SC an intrinsic SC property, or does it simply stem from an ordinary pairing in a doped Chern insulator? In addition, is the s-wave state obtained in the presence of LC order also topologically non-trivial?

○ **Reply 1-6**

Thank you very much for this important and insightful comment.

In our study, we focus on the topological numbers of the "superconducting gap function" and its breaking of time-reversal symmetry (TRS). In the revised manuscript, we have clarified the following findings in greater detail:

(i) LC-only case (without BO): The obtained d-wave gap function has a finite winding number w, indicating a topological SC state. The resulting complex SC gap implies broken TRS. In contrast, the s-wave gap function obtained for $\eta \neq 0$ is real (thus preserving TRS) and has $w = 0$.

(ii) LC+BO case: Due to the C_2 symmetry of the normal electronic state, an s +chiral d-wave SC state emerges, which necessarily breaks TRS. Here, w changes from zero to a nonzero when the weight of the d-wave component $\bar{\Delta}^d/\bar{\Delta}$ exceeds ~50%.

In the normal electronic state, LC order generates a finite Berry curvature and can give rise to interesting quantum-geometric effects, such as the anomalous Hall effect. However, the topological invariant is ill-defined because of the gapless metallic state.

However, based on our current understanding, we consider the central and most significant finding of the present study to be "the emergence of the nematic s + chiral d-wave SC state" in the nematic LC+BO phase in kagome metals. This state is characterized by a "**sizable $\bar{\Delta}^d/\bar{\Delta}$ that grows linearly with η.**" In the revised manuscript, we therefore emphasize that superconductivity with pronounced time-reversal symmetry breaking is realized, as evidenced by the ratio $\bar{\Delta}^d/\bar{\Delta}$ reaches values on the order of ~ 0.5 over a wide range of η. We have also added a discussion of the following experimental observations that support this scenario.

【Exotic emergent phenomena in the LC-induced chiral SC states】

In the SC states of kagome metals, a number of experiments have reported phenomena consistent with time-reversal-symmetry-breaking superconductivity, such as the zero-field superconducting diode effect and the emergence of π-flux states. The present study provides an important theoretical foundation toward resolving these outstanding experimental observations.

In particular, we focus on the "giant zero-field thermal Hall effect κ_{xy} below T_c" recently reported in Ref. [H. Yoshida et al, Sci. Adv. 11, eadu2973 (2025)]. The observed magnitude is several tens of times larger than the intrinsic thermal Hall effect expected from chiral edge states, $\kappa_{xy} = w\pi k_B^2 T/(6\hbar)$, and is therefore understood as an extrinsic (i.e., impurity-induced) thermal Hall effect. The extrinsic mechanism analyzed in a theoretical study [V. Ngampruetikorn et al., Phys. Rev. Lett. **124**, 157002 (2020)] can arise in the s + chiral d-wave state even when $w = 0$. Accordingly, a giant extrinsic κ_{xy} can naturally emerge in the LC+BO state based on the present work (see also **Reply 1-7**). Currently, we have begun a theoretical analysis of this mechanism; however, obtaining reliable and quantitatively robust results will require additional time. Therefore, in the revised manuscript, we restrict ourselves to a basic discussion based on previous theoretical studies that are not specific to the kagome lattice.

Action:

To clarify the above discussion on "**Exotic emergent phenomena in the LC-induced chiral SC states**" as an important direction for future scope, we have added the following two paragraphs in the SUMMARY Section in the main text:

(on pages 19 in the main text) "The present study offers crucial insight into the experimentally observed unconventional SC state in kagome metals, including the existence of phase degrees of freedom, time-reversal symmetry breaking, and strong coupling to the LC order. For example, zero-field SC diode effect and its relation to the LC chirality [43], switchable Little-Parks oscillation [71, 72], switchable chiral 2 × 2 pair density wave [65, 66], and zero-field Josephson diode effect [73, 74]. Notably, recent magnetic circular dichroism ARPES reports the time-reversal symmetry breaking in

CsV_3Sb_5 [40, 42].

Especially, recently observed giant thermal Hall effect in CsV_3Sb_5 below Tc [44] would be driven by from present TRS-breaking chiral SC state. The observed κ_{xy}^{exp} is several tens of times larger than that expected from the intrinsic mechanism of the chiral superconductor: $\kappa_{xy}^{int} = w\pi T/6\hbar$, where w is the winding number. Based on this observation, Ref. [44] attributed the gian κ_{xy}^{exp} to an extrinsic skew-scattering effect from dilute impurities [75]. The skew scattering mechanism also operates in the $s + d_{\pm}$ wave SC state, irrespective of the value of w. This is a highly important issue for future studies."

○ **Comment 1-7**

5) Is there any sign of TRS breaking in the obtained s-wave superconducting state under a pure LC phase? If not, how to understand the complete "screening" of the LC in the s-wave state? A related question: does the pairing angle also possess some small variations along the FS?

○ **Reply 1-7**

Thank you very much for this fundamentally important question.

In the revised manuscript, we have analyzed a generalized superconducting gap equation that allows for a finite center-of-mass momentum $\mathbf{Q}$ of Cooper pairs. As a result, we confirm that the eigenvalue $\lambda_{\mathbf{Q}}$ of the gap equation takes its maximum at $\mathbf{Q} = 0$ for $\phi, \eta \sim 0.05$, as shown in **Fig. R3**. (We note that spatial inversion symmetry is preserved in the nematic LC+BO state.) In the present work, we therefore restrict our numerical analysis to the superconducting gap equation at $\mathbf{Q} = 0$, and obtain the following results (This partially overlaps with **Reply 1-6**.):

(i) In the pure LC state ($\eta \neq 0, \phi = 0$), the s-wave gap function preserves TRS, in the sense that the phases of all gap components Δ_{m} ($\mathrm{m} = 1$–12) are identical.

(ii) In contrast, in the LC+BO state ($\phi, \eta \neq 0$), an s +chiral d-wave SC state is realized, in which the phases of Δ_{m} ($\mathrm{m} = 1$–12) are not aligned, corresponding to a SC state with broken TRS.

Below, we provide a more detailed explanation.

【SC gap equation for general Q】

Next, we discuss the possibility of the SC state with finite Cooper pair momentum **Q**. The gap equation for general q is given by

$$\lambda(Q)\Delta_m(Q) = g\sum_l \Gamma_{ml}(Q)\,\Delta_l(Q), \tag{R1}$$

$$\Gamma_{ml}(Q) = \frac{T}{N}\sum_{k,n} G^{ml}_{k+Q/2}(\epsilon_n)G^{ml}_{-k+Q/2}(-\epsilon_n)\,\Theta(\epsilon_n;\Omega) \tag{R2}$$

(Gap equations (1) and (2) in the main text correspond to **Eqs. (R1)** and **(R2)** at **Q**=**0**.)

In the LC+BO phase, the band dispersion E_b(**k**) preserves inversion symmetry. That is, the relation E_b(**k**) = E_b(-**k**) is satisfied. Under this symmetry condition, the eigenvalue λ(**Q**) in **Eq. (R1)** exhibits an extremum at **Q**=**0**. As shown in **Fig. R3**, the calculated λ(**Q**) attains its maximum value at **Q**=**0** in both the LC phase and the LC+BO phase.

On the other hand, when the LC+BO state and the "stripe-CDW" coexist, the relation E_b(**k**) = E_b(-**k**) is no longer satisfied reflecting the inversion-symmetry breaking electronic states, as we discussed in Ref. [R. Tazai et al., PNAS 122, e2503645122 (2025)]. In this case, λ(**Q**) should exhibit an extremum at **Q**≠0 due to the emergence of the **Q**-odd terms in the GL free energy. We would like to discuss this issue in a future publication.

Action

We have added the following paragraph at the end of the Subsection "**SC gap equation for kagome lattice model with 2 × 2density waves"** in the RESULTS Section:

(on page 7 in the main text) "In Eq. (1), we assume a zero Cooper-pair momentum **Q**. The validity of this assumption is verified in the LC+BO phase for ϕ,η≲0.05.

○ Comment 1-8

6) The authors mentioned prominent Berry curvature around line 91, but its relation to the emergence of the chiral d-wave state remains unclear. In particular, chiral SC states has been obtained on the kagome lattice from complex CDW Hamiltonians [arXiv: 2408.03056, 2504.02751]. The authors should provide more understanding regarding whether the chiral d-wave is favored due to a large Berry curvature or whether there is

connection between the two. A related question: how does the winding of the chiral d-wave order parameter along the FS relate to the Berry phase of the parent state?

○ **Reply 1-8**

Thank you very much for this important and thoughtful comment.

In the present theory, chiral SC state is induced by the complex pair-hopping amplitudes originating from the LC order parameter η, which are encoded in the complex phases of the kernel function $\Gamma_{l,m}$ shown in **Fig. 3**. We agree that the complex phases of $\Gamma_{l,m}$ are closely related to the Berry connection $\mathbf{A}_{\mathbf{k}}$ and the Berry curvature $\Omega_{\mathbf{k}} = \nabla_{\mathbf{k}} \times \mathbf{A}_{\mathbf{k}}$ in the band representation.

Motivated by this insightful reviewer's comment, in the revised manuscript, we focus on the fact the orbital motion of electrons in the LC phase gives rise to a macroscopic orbital magnetization (OM) $M_{\mathrm{orb}} = \sum_{\mathbf{b},\mathbf{k}} M_k^{orb}$, where M_k^{orb} is the orbital magnetization given by the quasiparticle at $\boldsymbol{k}$. At $T = 0$, $M_k^{orb} \sim \sum_b \left(\boldsymbol{m}_{\boldsymbol{k}}^{\boldsymbol{b}} + (E_{b,k} - \mu)\boldsymbol{\Omega}_{\boldsymbol{k}}^{\boldsymbol{b}}\right) f^{T=0}(E_{b,k})$, where $\boldsymbol{\Omega}_{\mathbf{k}}^{\boldsymbol{b}}$ is the Berry curvature and $\boldsymbol{m}_{\mathbf{k}}^{\boldsymbol{b}}$ is the self-rotating orbital magnetic moment. In **SI H**, we show that M_{orb} plays a crucial role in stabilizing the chiral SC state characterized by finite angular momentum of the Cooper pairs.

In **Figs. S10(c–e)**, we find that the LC-induced $M_{\mathbf{k}}^{orb}$ takes large values near the Fermi pocket around Γ. Thus, the LC-induced $M_{\mathbf{k}}^{orb}$ should act on the chiral Cooper pair (***k***, -***k***) on the Fermi pocket with finite angular momentum.

Furthermore, we have microscopically derived the Ginzburg–Landau free energy that describes the coupling between the LC and chiral SC in **Eq. (S19)**:

$$\boldsymbol{F}_{\mathbf{OM-ch}} = -\overline{\boldsymbol{M}}_{\boldsymbol{orb}}(|\boldsymbol{\Delta}_{\boldsymbol{d}-}|^2 - |\boldsymbol{\Delta}_{\boldsymbol{d}+}|^2),$$

where the GL coefficient $\overline{M}_{orb}$ has a functional form similar to M_{orb}. This free energy describes the bilinear coupling between the chiral Cooper pairs and LC-induced M_{orb}.

Owing to this ``**OM-chirality coupling mechanism**", the chiral SC state is stabilized in the LC phase in kagome metals. With these revisions and clarifications, we believe that the revised manuscript provides a clearer physical picture and offers improved insight into the mechanism of LC-induced chiral superconductivity. This discussion is clearly presented in detail in **SI H**.

We sincerely thank the reviewer for bringing to our attention the two important papers (arXiv:2408.03056 and arXiv:2504.02751), in which important interplay between the LC phase and chiral d-wave superconductivity has been revealed. In the present study, from the perspective of the OM-chirality coupling mechanism, we demonstrate the importance of carrier doping away from the vHS filling and low temperatures, and derive the $s+$ chiral d-wave state induced by the LC+BO phase, thereby providing an explanation for the key experimental observations.

We have added a corresponding discussion to the manuscript; see **Action**.

Action

To clarify the above important discussion on the "**OM-chirality coupling mechanism**", we have added **SI H**, "**Derivation of the GL free energy: OM-chirality coupling mechanism**" (on page 17- in the SI):

i. *Why the LC order enhances chiral d-wave superconductivity,*

ii. *Why the superconducting chirality is reversed under $\phi \rightarrow -\phi$,*

iii. *Outline of the derivation of Eqs. (S19) and (S20),*

iv. *Relation between M_{orb} and chirality of the SC gap.*

We have also added the following short paragraph in the SUMMARY Section: (on page 19 in the main text) *"In Refs. [arXiv:2408.03056, arXiv:2504.02751], the important interplay between the LC phase and chiral SC has been discussed. In the present study, from the perspective of the OM-chirality coupling mechanism, we demonstrate the importance of carrier doping away from the van Hove filling and low temperatures, and derive the s+ chiral d-wave state induced by the LC+BO phase, thereby providing an explanation for the key experimental observations."*

○ **Comment 1-9**

7) The Gamma-pocket appears essential in generating the chiral d-wave state and a pronounced 2×2 SC pattern. Do the Fermi surfaces in real materials exhibit similar dispersions?

○ **Reply 1-9**

Thank you very much for this insightful and relevant comment.

In **Fig. 1(c)**, we present the Fermi surface reconstructed by the 2×2 density wave in the folded Brillouin zone. However, for a direct comparison with ARPES experiments, the folded Fermi surface should be unfolded into the original Brillouin zone. We have therefore presented the "**unfolded Fermi surface**" in **SI G** and **Fig.S9**, where $\phi = -0.032$. (This unfolding procedure is performed by approximately restoring translational symmetry.) As a result, apart from the hybridization gap, the unfolded Fermi surface remains essentially unchanged. Moreover, finite experimental resolution and lifetime broadening cause the Fermi surface observed by ARPES in the 2×2 density-wave phase to appear similar to that of the original phase.

In summary, as correctly pointed out by the reviewer, the Fermi pocket around the Γ point in the folded Fermi surface plays a crucial role in the emergence of chiral superconductivity. This pocket is generically present due to band folding, provided that $|\eta|$ and $|\phi|$ remain sufficiently small ($\lesssim 0.05$eV).

Action

We have added the above discussions in **SI G**:"**Folded FS and unfolded FS**" (on page 17 in the SI)

○ **Comment 1-10**

8) In the analytical analysis of the pair-hopping mechanism depicted in Fig. 3(c), the imaginary part of the off-diagonal kernel function depends on the both BO (ϕ) and the LC (η) orders to an odd power. This implies that changing the BO from a SoD to a tri-hexagonal pattern may yield a different chirality of the SC state. It is surprising that a TRS breaking pattern is influenced by a TRS symmetric term. Could the authors provide further explanations for this?

○ **Reply 1-10**

We deeply appreciate the reviewer's important comment, which is actually essential for

the present s + chiral d-wave SC in the LC+BO phase.
As discussed in **Reply 1-8,** a finite orbital magnetization $M_{\rm orb}$ emerges in the LC phase. In this case, "OM-chirality coupling mechanism" stabilizes the chiral SC state. This mechanism can be intuitively understood as an additional term in the Ginzburg–Landau (GL) free energy, given by **Eq. (S19)** in **SI H.** Further analytical and numerical details are presented in **SI H**.

As explained in **SI H**, the leading-order term in the expansion of $M_{\rm orb}$ with respect to η and ϕ is given by $m_1\eta\phi$; see Ref. [R. Tazai et al., PNAS 121, e2303476121 (2024)]. Therefore, the sign of $M_{\rm orb}$ is reversed under the transformation $\phi \to -\phi$, as numerically demonstrated in **Fig. S10(a)**. Consequently, the LC-induced chiral SC state reverses its chirality under $\phi \to -\phi$.

An intuitive explanation for this sign reversal of $M_{\rm orb}$ is as follows. The pure LC state of kagome metals ($\phi = 0$, $\eta \neq 0$) can be regarded as a type of ferrimagnetic state. Although the local magnetization is linear in η, the η-linear contribution in $M_{\rm orb}$ vanishes due to the cancellation between fluxes in the $+z$ and $-z$ directions. Importantly, when BO is introduced ($\phi \neq 0$), this cancellation becomes incomplete, giving rise to an η-linear contribution to $M_{\rm orb}$. As a result, in the BO phase, a small η can induce a large chiral superconducting component.

In summary, the orbital magnetization $M_{\rm orb}$ generated by LC (and BO) order stabilizes one of the two chiral d-wave SC states with finite angular momentum, thereby favoring the chiral d-wave state over the s-wave state. We have added this physical interpretation to the main text and provided a more detailed discussion in **SI H**.

Action

To explain above important nature of the present LC-induced SC mechanism, we added the Subsection "**(ii)Why the superconducting chirality is reversed under ϕ → −ϕ**" in **SI H** (on page 19-20 in the SI).

We also added the following explanations in the Subsection "**Nematic chiral SC states driven by LC + BO order**":

(on page 13 in the main text) "In the LC+BO phase, a prominent chiral SC state emerges

due to the finite $\eta\phi$ *term in* θ_{ml}, as illustrated in Fig. 3 c. This $\eta\phi$ term is significantly enhanced when an LC-induced small Fermi pocket forms around the Γ point, since the process shown in Fig. 3 b becomes large owing to the enhanced magnitude of $|G_{Ak}||G_{Bk}|$ at $\boldsymbol{k} = \mathbf{0}$. The LC-induced chiral SC can be physically understood in terms of an "OM-chirality coupling mechanism" within the Ginzburg-Landau framework; see SI H [30] for details. As shown in SI K [30], the LC-induced chiral SC is further stabilized deep in the SC phase $T \ll T\mathrm{c}$."

○ Comment 1-11

10) The superconducting state, in spite of various interesting features such as nematicity and TRS breaking, doesn't appear to break additional symmetries beyond those already broken by the parent BO+LC state. In particular, it doesn't break additional translation symmetry beyond the CDW and is therefore a pair density modulation inside the CDW unit cell. Referring to it as a pair density wave would be confusing as it implied translation symmetry breaking. Could the authors clarify whether there are connections to a translation symmetry breaking 4a0 and 4a0/3 PDW as observed in experiments?

○ Reply 1-11

Thank you very much for this helpful comment. Following the usage adopted in recent literature, we have replaced the term 2x2 *pair-density wave (PDW)* with 2x2 *pair-density modulation (PDM)* in the revised manuscript.

In the revised manuscript, we have added numerical results based on the theoretical model with $t'/t = 0.16$ to **Methods B** and **Fig. 8**. In the LC+BO state, the PDM component reaches values of approximately 5%, which corresponds to the STM observations reported experimentally. In addition, a substantial nematic gap component is obtained, consistent with the pronounced nematic anisotropy in in-plane H_{c2} measurements [K. Fukushima et al., Nat. Commun. 15, 2888 (2024)].

These results provide a natural theoretical interpretation of key experimental observations and represent one of the central outcomes of the present study.

As for the $4a_0$ stripe CDW order observed below ~ 30 K in $A = \mathrm{Cs}$ and Rb, we have recently submitted a theoretical study focusing on a newly emerging nesting vector

in the reconstructed band structure induced by the 2×2 density wave; Y. Murata et al., arXiv:2511.06995 (submitted to PRB).

We are also very interested in the experimentally observed $4a_0/3$ pair-density-wave, which constitutes an important direction for future research.

Action

New numerical results of the pair-density-modulation in the 2x2 unit cell are presented in Fig. 8 in the section "**Methods B**" (on page 22 in the main text).

In addition, to express important direction of future study, we have added the following sentence at the bottom of the SUMMARY Section:

(on page 19 in the main text) "Another important research direction involves the $(4/3)¥times(4/3)$ pair-density-wave state and charge-4e and charge-6e superconductivity [46, 47], which remain intriguing open questions."

○ Comment 1-12

11) The Fermi surface shown in Fig. 4 seems to follow C6 symmetry, while the normal state is already nematic in the presence of BO and LC. Is it because the nematicity is relatively weak? The scale of the pairing gap is plotted from 0 to 1, which should be understood as the relative gap value. In that sense, the authors should further estimate the true gap value instead of its relative amplitude.

○ Reply 1-12

We thank the reviewer for this important and constructive comment. As noted by the referee, the BO and LC orders employed in the present study are not large (of order ~30meV); consequently, the induced nematic deformation of the FS is minimal.

In **SI K**, we have performed a careful analysis of the nonlinear superconducting gap equation below T_c and obtained several intriguing results. The numerical results are summarized in **Fig. S12**. In the $s+$ chiral d-wave state realized in the nematic LC+BO phase, the d-wave component just below T_c is just ~10%. However, surprisingly, it exhibits a pronounced temperature dependence and reaches about 40% for $T \ll T_c$. This calculation may provide a possible explanation for the crossover-like rapid temperature

evolution of the superconducting gap reported in Ref. [M.S. Hossain et al., Nat. Phys. 21, 556 (2025)]. Moreover, the thermal Hall effect reported in Ref. [H. Yoshida *et al.*, Sci. Adv. 11, eadu2973 (2025)] is observed to grow rapidly below $T \sim 1\text{K}$ ($\ll T_c$), which is consistent with our numerical results shown in **Fig. S12(d)**.

A more detailed quantitative analysis of various physical quantities below T_c, based on fully self-consistent calculations, is left for future work. We again thank the reviewer for this helpful comment.

Action

The novel and pronounced enhancement of the d-wave SC weight within the s + chiral d-wave SC phase, obtained by the nonlinear superconducting gap equation, is explained in **SI K: "Analysis of nonlinear gap equation in the LC+BO phase"** (on page 26 in the SI).

○ Comment 1-13

12) The superconducting solution is proposed to be nematic in the presence of bond order and loop current. Is the nematic direction of the normal state the same as that in the SC state? Would the result be consistent with the experimental observations in, e.g. Nat. Comm. 12, 6727?

○ Reply 1-13

Thank you very much for this useful comment on the in-plane anisotropy of the upper critical magnetic field H_{c2} reported in Refs. [Y. Xiang et al., Nat. Commun. 12, 6727 (2021)].

In the **SI I**, we explain the anisotropy of H_{c2} in the nematic chiral SC state. Below, we describe a brief excerpt. In the clean region, H_{c2} due to orbital depairing effect is: $H_{c2}^{\mu} = \Phi_0/2\pi\xi_\nu\xi_z$, where μ, ν = x, y and μ≠ν. ξ_μ is the coherence length along μ direction. Then, in-plane anisotropy of H_{c2} is $H_{c2}^{x}/H_{c2}^{y} = \xi_x/\xi_y$.

In the BCS theory, the coherence length is expressed as $\xi_\mu^2 \propto \langle v_{k,\mu}^2 |\Delta_k|^2\rangle_{FS} (\bar{\Delta}\,T_c)^{-2}$,

where $v_{k,\mu}$ is the μ component of the electron velocity at **k**. Therefore, we obtain

$$\frac{H_{c2}^{x}}{H_{c2}^{y}} = \frac{\langle v_{k,x}^2 |\Delta_k|^2\rangle_{FS}^{1/2}}{\langle v_{k,y}^2 |\Delta_k|^2\rangle_{FS}^{1/2}}$$

In kagome metal, the Fermi velocity approximately possesses C_6 symmetry even in the LC+BO phase. In contrast, $|\Delta_k|$ exhibits prominent C_2 symmetry in the nematic chiral SC state, as demonstrated in **Figs.4 (b)** and **(e)** in the main text. Therefore, experimental in-plane anisotropy of H_{c2} originates from the in-plane anisotropy of $|\Delta_k|$.

In this paper, y-direction corresponds to the nearest V-V direction. For the nematic LC+ SoD BO state shown in **Fig.4 (b)**, we obtain $\xi_x/\xi_y = H_{c2}^x/H_{c2}^y < 1$, in agreement with Ref. [Y. Xiang et al., Nat. Commun. 12, 6727 (2021)].

Importantly, another measurement of the in-plane anisotropy of H_{c2} [K. Fukushima et al., Nat. Commun. 15, 2888 (2024)] reports that $(H_{c2}^x - H_{c2}^y)/(H_{c2}^x + H_{c2}^y)$ becomes finite just below T_c, indicating that the electronic state is nematic even in the norma state. This report is consistent with the present theory of the nematic chiral SC state in the C_2 symmetry LC+BO phase.

Action

We have added the above discussion in **SI I: "In-plane anisotropy of H_{c2} in the nematic SC state"** (on page 23 in the SI).

○ Comment 1-14

13) It would be helpful if the authors could comment on the SC state on the hole-doped side with respect to the van Hove singularity. As one would expect a different dispersion in the hole-doped region, would the chiral d-wave remain favored?

○ Reply 1-14

We thank the reviewer for raising this important question regarding the filling dependence of chiral superconductivity.

In the revised manuscript, we have newly investigated the filling-number (n) dependence of the eigenvalues of the superconducting gap equation, and the results are presented in **Fig. 9** of **Method C**. The calculations were performed for $\eta = 0.032$eV, $\phi = 0$, and $T = 0.8$meV. At the van Hove filling $n \sim 11$, an s-wave SC state is realized. In contrast, chiral SC state emerges at $n \sim 11.8$ (electron-doped side) and $n \sim 10.2$ (hole-doped side), where the relation $|E_{\rm vHS}^{xz} - \mu| = 0.05\sim 0.15$ eV is satisfied.

Interestingly, for a fixed η, the d-wave state exhibits opposite chiralities at $n \sim 11.8$ and $n \sim 10.2$. Therefore, the LC-induced chiral superconductivity persists on both the electron-doped and hole-doped sides, indicating a degree of generality of the proposed mechanism. We thank the reviewer for prompting this important analysis.

Interestingly, we find that the LC-induced orbital magnetization M_{orb} also changes its sign between $n = 10.2$ and $n = 11.84$. The reversal of the d-wave SC chirality accompanying the sign change of M_{orb} further supports the "OM-chirality coupling mechanism", which is a key finding in the revised manuscript.

Action

To clarify the electron-filling dependence of the superconducting phase and the chiral superconducting state in the hole-doped region, we have added the following subsections:

・**Method C: "*n*-dependence of λ_x (*x* = *s, d*)"** (on page 22 in the main text)**,**

・**Method D: "LC-induced chiral d-wave state for hole-doped system (*n* = 10.2)"** (on page 23 in the main text)

Reply to the Comments by Reviewer #2

○ Comment 2-1

The paper entitled Nematic chiral Superconductivity driven by chiral loop current order in kagome metals by Rina Tazai, Youchi Yamakawa, Hiroshi Kotani sets out to explain recent experimental results reporting superconducting states in the of the Kagome metals (AV3Sb5) with TRSB, nematicity, PDW modulation, and a transition to s-wave SC in the presence of disorder.

○ Reply 2-1

We are grateful to Reviewer #2 for the careful evaluation of our manuscript and for the many constructive and insightful comments. These suggestions have enabled us to substantially improve and strengthen the previous version.

Below, we detail the main revisions.

○ Comment 2-2

The authors consider a simple tight-binding model for the Kagome lattice with hoppings up to next-nearest-neighbours. The authors "assume" that the system has developed loop-current (LC) or charge-bond order (BO), placing in "by-hand" the (possibly) complex modulations of the hopping parameters.

○ Reply 2-2

We thank the reviewer for this important comment. In the present study, we focus on the emergence of exotic superconductivity under the strong influence of the LC+BO orders. To this end, we treat the BO and LC orders as static background fields. This approximation is well justified, as their characteristic energy scales exceed that of the superconducting gap by more than an order of magnitude. Given this clear separation of energy scales, any feedback effects on the BO and LC orders below T_c are expected to be negligible, even within a fully self-consistent treatment.

Based on the above considerations of the energy hierarchy, treating the high-energy LC

and BO orders as fixed background fields—an approach motivated by previous experimental and theoretical studies—while focusing on the low-energy superconducting sector constitutes a well-justified and productive strategy. Accordingly, we have concentrated on the superconducting instability and identified the key underlying mechanisms. The hierarchical energy structure is discussed in detail in Supplementary Information H (**SI H**). Additional discussion is provided in a subsequent Reply.

○ **Comment 2-3**

The authors examine the behaviour of the linearized gap equation in the presence of loop-current (LC) order. In the absence of LC order, the s-wave state is selected. In the presence of LC order, a chiral d-wave state is selected. The authors use a 12-site superlattice corresponding to the 2x2 CDW order. The d-wave state is shown to display a nonzero (~5%) dependence within the supercell sublattice, i.e. a PDW.

○ **Reply 2-3**

In the revised manuscript, we have refined the tight-binding model for the kagome metal, as detailed in the Reply below. As a result, the numerical results are modified quantitatively, while the central physical conclusions remain unchanged. The obtained pair-density modulation (PDM; previously referred to as PDW), of order ~5%, is comparable to experimental observations [H. Deng et al., Nature 632, 775 (2024)]. The updated numerical results are presented in the revised **Fig. 8**.

Action

New numerical results of the pair-density-modulation in the 2x2 unit cell are presented in Fig. 8 in the "**Methods B**" Section (on page 22 in the main text).

○ **Comment 2-4**

The authors provide some explanation for this behaviour by presenting a phenomenological kernel function with only three parameters based on their numeric results of the full kernel. The phenomenological kernel is effective in explaining which SC state is selected, assuming the numeric results. However, the attempt to explain the mechanism underlying the numerics themselves (i.e. Fig. 3a-d) may be difficult to follow

for readers unfamiliar with the formalism.

○ **Reply 2-4**

We thank the reviewer for this very important comment. To address this comment, we have made the following revisions to provide a clearer physical understanding of the LC-induced chiral superconductivity mechanism proposed in this work.

(i) In **Fig. 3,** we show only the two dominant diagrams for the imaginary part of the pair-hopping amplitude $i\gamma''_{l,m}$ $(l \neq m)$, which stabilizes the chiral SC state.
(ii) In **SI H,** we newly derive the Ginzburg–Landau free-energy that describes the LC-induced chiral superconducting transition:

$$\boldsymbol{F_{\mathrm{OM-ch}} = -\bar{M}_{orb}(|\Delta_{d-}|^2 - |\Delta_{d+}|^2)},$$

where $\bar{M}_{orb}$ has a functional form similar to M_{orb}. This simple free energy describes the '**OM-chiraltiy coupling mechanism**,' whereby chiral SC state is stabilized via a bilinear coupling between the LC-induced orbital magnetization (OM) M_{orb} and the chiral Cooper pairs. This mechanism becomes more significant for $T \ll T_c$; see **SI K**.
(iii) In **Fig. S10**, we present careful numerical analysis that support the '**OM-chiraltiy coupling mechanism**.'

With these revisions, we believe that the physical interpretation underlying our main results is now presented more clearly, thereby enhancing the overall clarity and accessibility of the manuscript. We thank the reviewer for this valuable suggestion.

Action

We have added the following important discussions in the revised manuscript:
・**SI H** (on page 17 in the SI): We derived the Ginzburg–Landau free-energy theory and revealed the "**OM-chiraltiy coupling mechanism**," whereby chiral SC state is stabilized via a bilinear coupling between the LC-induced OM and the chiral Cooper pairs.
・**Fig. S10**: We present careful numerical analysis that support the "OM-chiraltiy coupling mechanism."

○ **Comment 2-5**

The authors cite their previous work [Nat. Comm. 14, 7845 (2023)] to invoke nematicity. According to that paper, nematic order develops when the centres of symmetry of the CO and LC orders are out of phase. The authors then study the behaviour of the gap equation in the presence of coexisting CO and LC orders, finding that the SC gap is also nematic, even though the Fermi surface retains its nearly-perfect C6 symmetry. The selected superconducting state is s + chiral d-wave. The authors invoke Anderson's theorem to explain the suppression of the chiral component in the presence of non-magnetic impurities, but do not support this claim quantitatively.

○ **Reply 2-5**

We thank the reviewer for the accurate summary of our findings on the $s+$chiral d-wave SC state. During the revision process, we carried out a dedicated theoretical analysis to clarify the crossover from a chiral d-wave–type gap to an s-wave–type gap induced by non-magnetic impurities. In addition, we demonstrate the realization of the Anderson theorem in the dense-impurity regime (i.e., where the s-wave SC transition temperature becomes independent of n_{imp}). The results are summarized in **SI J**, and the corresponding numerical data are presented in **Fig. S11**.

Figure S11(a) shows the eigenvalue λ approaches unity with increasing n_{imp} for g=2.4~2.7. (Here, T=0.8meV.) In particular, for g=3.2, the relation $\lambda \approx 1$ is realized for $n_{\mathrm{imp}} \gtrsim 3\%$, indicating that T_c =0.8meV is nearly independent of n_{imp}. In addition, **Fig. S11(c)** shows that a simple s-wave SC state is realized for $n_{\mathrm{imp}} \gtrsim 1\%$. This behavior is aallmark of the Anderson theorem.

These results constitute one of the main outcomes of the revised manuscript. More detailed and quantitative analyses will be an important subject for future work.

Action

We added the explanation on the impurity-induced crossover from a chiral d-wave type gap function to a s-wave type gap function in **SI J: Remarkable nonmagnetic impurity effects on the $s+d_{\pm}$ wave SC states"** (on page 24 in the SI)

In addition, the novel and pronounced enhancement of the d-wave SC weight within the s + chiral d-wave SC phase, obtained by the nonlinear SC gap equations for $T \ll T_c$, is

explained in **SI K: "Analysis of nonlinear gap equation in the LC+BO phase"** (on page 26 in the SI).

○ **Comment 2-6**

I have following questions:

1. The tight-binding model used is very simple, and assumes filling at the p-type vHS. Real Kagome metals have multiple vHS near the FS. How do the authors justify the use of this model compared to a more realistic tight-binding model based on DFT (e.g. arXiv:2507.16892)? Does the mechanism rely on a p-type vHS?

○ **Reply 2-6**

We thank the reviewer for the important comment regarding the multiband nature of the kagome metal model. In the revised manuscript, we have introduced a minimal yet realistic three-dimensional $xz + yz$ orbital model. This model contains six d orbitals per unit cell. The resulting band structure and Fermi surfaces are shown in **Figs. S5** and **S6**, respectively, demonstrating that the $xz + yz$ orbital band structure obtained from first-principles calculations is well reproduced. Furthermore, the present model captures the substantial gapping of large portions of the band contours at $E \approx E_{\rm Dirac}$ due to strong inter-orbital hybridization, as illustrated in **Fig. S6(c)**.

In **Reply 1-4**, we summarized the essential ingredients of the $xz + yz$ orbital model.

We are grateful to the reviewer for drawing our attention to the important work arXiv:2507.16892. In that study, a "13-orbital unit cell model" is analyzed, which is derived from a first-principles 30-orbital unit cell model by retaining six d-orbitals and seven p orbitals in the unit cell. As long as one focuses on the $xz + yz$ orbitals, this model is essentially equivalent to our $xz + yz$ orbital model.

As we show in **Fig. S5,** the bands derived from the yz orbital exhibit significant k_z dispersion, with the van Hove singularity energies located at approximately $+0.1$eV on the $k_z = 0$ plane and -0.2eV on the $k_z = \pi$ plane. To properly capture this feature, we do not employ a two-dimensional approximation. Instead, we carry out a fully three-

dimensional numerical analysis, despite the associated computational complexity.

(In a two-dimensional approximation, as adopted in Ref. [arXiv:2507.16892], the vHS energy lies close to $E_{\rm F}$ for all k_z, which may lead to an overestimation of the yz-orbital contribution.)

In **SI E**, we present the results of our extended analysis based on the three-dimensional $xz + yz$ orbital model. As shown in **Fig. S7**, the chiral SC state emerges over a broad parameter range in η and filling n, which is even wider than in the two-dimensional xz-orbital model owing to the inter-orbital hybridization. Therefore, the theoretical analysis based on the two-dimensional xz-orbital model presented in the main text captures the essential physics.

The newly added **SI E** thus not only reinforces the robustness of the LC-induced chiral SC mechanism, but also introduces a minimal yet realistic three-dimensional $xz + yz$ orbital model that can serve as a useful platform for future theoretical investigations.

Note that, in **Fig. S7**, the pairing interaction g and the LC η are introduced only on the xz orbitals. We have verified, however, that including g and η also on the yz orbitals yields essentially the same results, owing to the large xz-orbital DOS at E_F. Notably, recent magnetic circular dichroism ARPES experiments have reported broken time-reversal symmetry predominantly associated with the **xz-orbital vHS** [H. J. Elmers et al., PRL 134, 096401 (2025); H. J. Elmers et al., arXiv:2511.01238 (2025)].

Action

To clarify the above discussion, we have added **SI E**, "**chiral d-wave superconductivity in three-dimensional two-orbital kagome metal model**" (on page 10 in the SI), which includes three important figures:

・**Fig. S5**. **Two-orbital 3D kagome lattice model parameters**; the characteristic band dispersion near E_F is properly reproduced.

・**Fig. S6**. **Fermi surfaces of 3D kagome lattice model**; the pronounced inter-orbital band reconstruction around the vHS energy is properly reproduced.

・**Fig. S7. LC-induced chiral d-wave SC states in 3D kagome lattice model**; the chiral SC state is robustly obtained.

In addition, we added the following short paragraph to the SUMMARY Section:

(on page 17 in the main text) “We note that a two-dimensional 13-orbital model, in which seven additional p orbitals are introduced to the present model, has been analyzed in Ref. [arXiv:2507.16892]. In that work, the authors discuss a novel LC-fluctuation-mediated SC mechanism.”

○ Comment 2-7

2. The amplitudes ¥phi and ¥eta for the bond orders are put in by hand, and tuned freely. How do the authors justify their choices of these amplitudes in comparison with experiment or self-consistent theoretical calculations? How does it affect the constant attractive interaction "g", as the attractive interaction is due to BO fluctuations?

○ Reply 2-7

We are grateful to the reviewer for raising this important question regarding our research approach. In kagome metals, the LC and BO order parameters (η and ϕ) exceed the superconducting gap Δ by more than an order of magnitude, implying that their variation within the SC state is expected to be small. This clear separation of energy scales justifies the present approximation. The values of η and ϕ are chosen within experimentally suggested ranges. The current understanding of the hierarchy of energy scales, based on both theoretical and experimental studies, is discussed in detail in **SI F** (“**Hierarchy of Energy Scales in Kagome Metals**”).

Based on the above, introducing the high-energy-scale LC and BO orders as constants—on the basis of previous experimental and theoretical findings—and investigating low-energy superconductivity is a well-justified and productive strategy. Accordingly, we concentrated our efforts on superconductivity and identified key physical insights.

Below, we summarize the key points discussed in **SI F**. As noted in **Reply 1–3**, the experimental values of the LC (η) and BO (ϕ) parameters remain under debate. Nevertheless, several ARPES studies have provided rough estimates suggesting $\phi \sim$ 30meV and $\eta \lesssim \phi$. Importantly, the chiral superconducting state is realized over a broad range of (η, ϕ). As shown in **Figs. 4d** and **5e**, chiral superconductivity persists even in

the regime $\eta < \phi$.

【Emergence of attractive interaction: RG theory】

The experimental hierarchy of temperature scales **“$T_{BO} \gtrsim T_{LC} >> T_{s\text{-}SC} \sim T_{d\text{-}SC}$“** is naturally obtained by the parquet RG analysis presented in the Supplemental Information (Note 5) of Ref. [Tazai et al., Nat. Commun. 14, 7845 (2023)], when the inter-site attractive interaction (v) is much smaller than the Coulomb repulsion (U): In **Fig. S8** of the **SI F**, we show the numerical results of (a) the renormalized interactions g_1~g_4 and (b) the instabilities for the channel X, G_X (X = rCDW, iCDW, rSDW, iSDW, s-SC and d-SC) for U=0.9 and v =0.6. The horizontal axis denotes the RG parameter $z = \ln^2(E_0/T)$, where E_0 is the cutoff-energy (E_0~|t|) and T is the temperature: z = 12 corresponds to T= E_0/30.
In **Fig.S8 b**, the obtained instabilities G_X shown $G_{rCDW} \sim G_{iCDW} >> G_{s\text{-}SC} \sim G_{d\text{-}SC}$ for z > 10: G_{rCDW} (G_{iCDW}) represents the BO (LC) instability, and $G_{s\text{-}SC}$ ($G_{d\text{-}SC}$) represents the s-wave (d-wave) SC instability. Thus, the parquet RG analysis provides a consistent understanding of the relationship among the energy (temperature) scales of the three orders—bond order, loop-current order, and superconductivity—despite their common origin in the charge-channel interactions.

The increment of $G_{s\text{-}SC} = -g_4 - 2g_3$ and $G_{d\text{-}SC} = -g_4 + g_3$ originates from the large negative value of g_4 obtained in **Fig. S8 a**. Since negative g_4 corresponds to an intra-sublattice attraction, this result supports the present on-site attractive (*g*) model within the parquet RG framework. However, a fully microscopic calculation of the pairing interaction *g* inside the 2x2 LC+BO phase remains technically challenging.

【Pairing interaction inside the BO phase】

We thank the reviewer for this important question regarding the pairing interaction g in the 2x2 BO phase ($\phi \neq 0$). NMR experiments in CsV_3Sb_5 [X.Y. Feng et al., npj Quantum Materials 8, 23 (2023)] show that BO fluctuations are maximized at the critical pressure where BO disappears ($P_c = 2$ GPa), with T_c reaching ~10 K. These observations support the BO fluctuation mediated SC in kagome metals. In principle, BO fluctuations below T_{BO} can be evaluated by solving the density-wave (DW) equation for the 12-site unit-cell kagome model. (see, *e.g.,* our previous analysis of nematic fluctuations below T_{nem} in

FeSe [R. Tazai et al., PRB 107, 035137 (2023)].) However, carrying out the DW equation analysis for the 12-site model is technically highly demanding.

On the other hand, spin fluctuations the 12-site unit-cell kagome model can be treated within the random-phase approximation (RPA). In the revised manuscript, we have performed this analysis and demonstrate that the "LC-induced chiral SC mechanism" persists even within a spin-fluctuation pairing framework, highlighting its universality. The details are provided in **Reply 2–9.**

Action

To clarify the above important discussion on the energy hierarchy in kagome metals, we added **SI F**: "**Hierarchy of Energy Scales in Kagome Metals**" (on page 14 in the SI).

i. *Emergence of the s wave SC and d wave SC instabilities derived from the parquet RG*,

ii. *Magnitude of the bond order ϕ*,

iii. *Magnitude of the LC order η*,

iv. *Magnitude of the superconducting gap.*

○ Comment 2-8

3. While it was claimed that the proposed mechanism qualitatively reproduces a number of observed features of the SC state (TRSB, nematicity, PDW, disorder), the connection to experiment is largely descriptive. Can the authors offer more specific connections to experiment?

○ Reply 2-8

We thank the reviewer for this important comment.

Recently, a number of highly unconventional superconducting phenomena have been reported in kagome metals, attracting considerable attention. Prominent examples include a giant thermal Hall effect that is strongly enhanced in the superconducting state and a zero-field superconducting diode effect (SDE). Recent SDE measurements, with careful control of magnetic-field history, have revealed that the polarity of the SDE is determined by the LC order in the normal state ($T^* \sim 30$ K). (See the discussion in **Reply 1-6** under 【Exotic emergent phenomena in the LC-induced chiral SC states】.)

These observations strongly suggest the realization of chiral superconductivity. We

anticipate that the present theoretical framework will contribute to a unified understanding of these phenomena. We have also initiated investigations of the giant thermal Hall effect and the zero-field SDE within this framework [V. Ngampruetikorn et al., Phys. Rev. Lett. **124**, 157002 (2020)], and further results will be reported elsewhere.

Action

To clarify the above discussion on "**Exotic emergent phenomena in the LC-induced chiral SC states**" as an important direction for future scope, we have added the following two paragraphs in the SUMMARY Section in the main text:

(on pages 19 in the main text) "The present study offers crucial insight into the experimentally observed unconventional SC state in kagome metals, including the existence of phase degrees of freedom, time-reversal symmetry breaking, and strong coupling to the LC order. For example, zero-field SC diode effect and its relation to the LC chirality [43], switchable Little-Parks oscillation [71, 72], switchable chiral 2 × 2 pair density wave [65, 66], and zero-field Josephson diode effect [74, 75]. Notably, recent magnetic circular dichroism ARPES reports the time-reversal symmetry breaking in CsV_3Sb_5 [40, 41].

Especially, recently observed giant thermal Hall effect in CsV_3Sb_5 below Tc [44] would be driven by from present TRS-breaking chiral SC state. The observed $\kappa_{xy}{}^{\rm exp}$ is several tens of times larger than that expected from the intrinsic mechanism of the chiral superconductor: $\kappa_{xy}{}^{\rm int} = w\pi T/6\hbar$, where w is the winding number. Based on this observation, Ref. [44] attributed the gian $\kappa_{xy}{}^{\rm exp}$ to an extrinsic skew-scattering effect from dilute impurities [75]. The skew scattering mechanism also operates in the $s + d_\pm$ wave SC state, irrespective of the value of w. This is a highly important issue for future studies."

○ **Comment 2-9**

4. The authors assume a constant attractive interaction. How do the authors expect their results to change with a more sophisticated treatment of the pairing interaction like momentum dependence, etc?

○ **Reply 2-9**

We sincerely thank the reviewer for this important comment regarding the robustness and generality of the proposed mechanism. In the previous version of the manuscript, we demonstrated the LC-induced chiral SC mechanism within a minimal local attractive-interaction (g) model. The simplicity of this framework allowed us to analyze the gap equation in detail and provide a transparent physical interpretation. We agree, however, that the universality of the LC-induced chiral SC mechanism should be examined within a broader class of nonlocal pairing interactions.

Below, we briefly summarize the newly added RPA analysis (see **SI D** for details). By optimizing the gap function, we find that a nodal s-wave SC state emerges as the leading instability. The subleading eigenvalues correspond to a twofold-degenerate d-wave SC state. The resulting gap structures are shown in **Figs. 5b** and **5c**. The η dependence of the eigenvalues is presented in **Fig. 5(d)** ($\phi = 0$) and in **Fig. 5(e)** ($\phi = -0.024$). These results indicate that chiral SC state is realized over a relatively broad parameter range.

With these additional calculations, we demonstrate the generality of the LC-induced chiral SC mechanism proposed in the present work. We thank the reviewer again for the insightful comments that motivated this analysis.

Action

To examine the generality of the proposed LC-induced chiral superconductivity mechanism, we further performed an analysis of the spin-fluctuation–exchange mechanism within a local repulsive interaction (U) model, which serves as a prototypical nonlocal pairing mechanism.

This new result is added as **SI D: "RPA analysis of kagome lattice Hubbard model"** (on page 6 in the SI), and the numerical results are presented in Fig. 5 and Fig. S4.

○ Comment 2-10

5. Nematicity is introduced by the coexistence of LC and CB order, as was previously argued by the authors in Nat. Comm. 14, 7845 (2023). It is not clear to me what is novel in this work compared to their previous publications (Refs. 10, 37, and 38), aside from introducing the ad hoc constant g and studying the pairing instabilities. Since SC, LC, and CB are all broken-symmetry states, should they be treated on equal footing?

○ **Reply 2-10**

We consider that the novelty of the present work lies in providing a unified understanding of chiral and nematic superconductivity—central issues in kagome metals—by starting from the experimentally observed electronic state characterized by coexisting LC and BO orders and identifying the underlying the "OM-chiraltiy coupling mechanism." Because this mechanism promotes chiral superconductivity irrespective of the detailed form of the pairing interaction (repulsive or attractive), it possesses a high degree of universality.

The microscopic origins of the LC and BO orders remain unresolved. However, there is broad consensus that the nesting vectors connecting the three van Hove singularities at the M points drive the 2×2 BO and LC orders, and increasing experimental evidence supporting this scenario has recently accumulated (see the **Action** in **Reply 1–6**). In light of these considerations, we introduce the 2×2 BO and LC orders as model parameters, without specifying their microscopic origin.

Furthermore, as already discussed in **Reply 2-7**【Emergence of attractive interaction: RG theory】, we analyze a kagome metal model including onsite repulsion (U) and bond-order interaction (v) using parquet renormalization-group (RG) theory. Within the RG framework, LC, BO, and SC are treated on an equal footing. In **SI F** of the revised manuscript, the experimentally observed hierarchy of temperature scales, **"$T_{BO} \gtrsim T_{LC} \gg T_{s\text{-}SC} \sim T_{d\text{-}SC}$"**, is naturally reproduced by the parquet RG analysis presented in this work. In view of this hierarchy, we conclude that treating the LC and BO orders as essentially constant below T_c is a well-justified approximation.

However, as mentioned by the reviewer. achieving a fully unified understanding of BO, LC, and SC, remains an important open problem, as we stress at the end of the SUMMAY Section:

(on page 19 in the main text) "In this study, we examined exotic states under the strong influence of LC+BO by approximating ϕ and η as constants, justified by the hierarchy of energy scales $\phi, \eta \gg \Delta$. However, the achievement of a unified understanding of BO, LC,

and SC, as initiated in Refs. [27, 28], remains the ultimate objective."

In summary, the LC-induced chiral SC mechanism developed in this work provides a unified and physically transparent interpretation in terms of an "OM-chirality coupling mechanism," independent of the specific form of the pairing interaction. Importantly, our formulation does not depend on the microscopic origin of the BO and LC orders, underscoring the robustness and generality of the proposed mechanism.

○ Comment 2-11

Minor points (mainly typos):

a. Change "sensitively" to "sensitivity" in abstract

b. Change "disorders" to "disorder" in abstract

c. Change "holded" to "folded" on line 173

d. Lines 243 and 244 are repeated on lines 254 and 255.

f. References [4] and [8] are repeated.

○ Reply 2-11

We thank the reviewer for pointing out these minor issues. All of the listed points have been corrected in the revised manuscript.

○ Comment 2-12

In summary, I do not recommend the publication unless the above points are addressed in a satisfactory manner.

○ Reply 2-12

We once again express our sincere gratitude to Reviewer #2 for the careful reading of our manuscript and the insightful comments. We have carefully addressed all constructive suggestions and, guided by these valuable comments, performed important additional calculations, thereby significantly enhancing the robustness and clarity of the manuscript. As a result, we believe that the overall clarity and completeness of the work have been significantly improved. We thank you for your time and consideration and hope that the revised manuscript is now suitable for publication.

Reply to the Comments by Reviewer #3

○ Comment 3-1

In the manuscript, the authors seek to combine the reported experimental evidence for kagome metals into a consistent formation mechanism of superconductivity. The chosen key aspects from the authors' opinion are time-reversal symmetry breaking, nematicity, and a propensity for pair density wave formation. This phenomenological ansatz adopted by the authors is not obvious. For instance, it is rather a subtle point to assess the current experimental evidence as to whether time reversal symmetry is broken in the kagome superconductors or not: while muSR supports this claim, Kerr spectroscopy discards this possibility. The same holds for pair density wave signatures: it is not clear whether there are just electrons proximized by a charge order which when suggests to be a pair density wave. Only nematicity is largely undisputed uniformly shown by different experimental techniques.

○ Reply 3-1

We are grateful to Reviewer #3 for the careful evaluation of our manuscript and for the important and insightful comments. These suggestions have enabled us to substantially strengthen and improve the previous version. Below, we respond to the reviewer's comments in detail.

【Experimental evidences of LC order】

Here, we briefly review recent experimental studies reporting time-reversal-symmetry breaking in the normal state of kagome metals. Although this issue remains under active debate, accumulating evidence increasingly points toward intrinsic symmetry breaking. The 2×2 loop-current (LC) order—one of the leading candidates—generates not only local (alternating) orbital magnetization detected by μSR, but also a finite uniform orbital magnetization (OM) $M_{\rm orb}$. While $M_{\rm orb}$ is just of the order of $10^{-3}\,\mu_{\rm B}$, its presence has been reported by electrical magneto-chiral anisotropy (eMChA) measurements [C. Guo et al., Nature 611, 461 (2022)], magnetization measurements using tuning-fork resonators [H. Gui et al., Nat. Commun. 16, 4275 (2025)], and chiral quasiparticle-interference signals

observed by STM under magnetic fields [H. Deng et al., Nature 632, 775 (2024)]. These observations are naturally accounted for within the LC framework [R. Tazai et al., PNAS 122, e2503645122].

By contrast, Kerr-effect measurements [D. R. Saykin et al., PRL 131, 016901 (2023)] have not yet converged. Possible staggered orbital magnetization between layers or domain formation may reconcile this apparent discrepancy.

Notably, very recent magnetic circular dichroism ARPES (MCD-ARPES) experiments have reported time-reversal-symmetry breaking predominantly associated with the **xz-orbital vHS** [H. J. Elmers et al., PRL 134, 096401 (2025); H. J. Elmers et al., arXiv:2511.01238 (2025)]. In addition, magnetic-field-history-dependent measurements of the superconducting diode effect (SDE) indicate the onset of LC order at $T^* \sim 30\mathrm{K}$ [G. Ge et al., arXiv:2506.04601 (2025)].

Taken together, this growing body of experimental and theoretical evidence strongly motivates a systematic investigation of LC-induced superconductivity. In view of recent reports of unconventional phenomena—such as the zero-field SDE and half-vortex states—establishing a coherent theoretical framework for LC-induced chiral superconductivity is not only timely but essential for resolving several outstanding experiments in kagome metals.

Action

We have strengthened the paragraph in the Introduction Section concerning the experimental reports of LC by incorporating recent experimental findings:

(on page 3 in the main text) "The chiral LC phase accompanies finite uniform orbital magnetization (M_{orb}), which can be switched by a small magnetic field *Bz*, as evidenced by the anomalous Hall effect [32], electronic magneto-chiral anisotropy (eMChA) [33, 34], and magnetic torque [13] measurements. Magnetotropic susceptibility measurements reveal a small but heavily anisotropic M_{orb} with tiny hysteresis [18]. Another direct evidence of LC comes from scanning tunneling microscopy (STM) measurements: The chirality in the quasiparticle interference (QPI) is switched by *Bz* in both *A*=Cs and *A*=K compounds [12, 14−17], which is naturally induced by the LC [35, 36]. In addition,

internal local magnetic field in the LC phase has been reported by μSR [37, 38] measurements. In contrast, Kerr-effect studies reported the absence of LC [40]. Recently, magnetic circular dichroism ARPES has measured the orbital magnetic moments mainly on the *xz*-orbital band in CsV_3Sb_5 [40, 41]. Also, the SC diode polarity reflects the normal-state LC chirality in Ref. [43]."

○ **Comment 3-2**

Starting from their phenomenological assumptions, the authors employ sophisticated mean field ansätze to follow up on their suggestion of an s+(d+id) superconducting state, which is the central new proposal in the manuscript in comparison to the current states quo. This finding must be taken with some reservation, as there are so many competing orders involved that any mean field ansatz might fall for its systematic bias.

○ **Reply 3-2**

We thank the reviewer for recognizing the novelty of the s-dominant chiral d-wave SC state, which constitutes the central result of the present work.

In the present study, we focus on the emergence of exotic superconductivity under the strong influence of the LC+BO orders. The **novel LC-induced nematic chiral SC state** is the main finding of the present theory. The present study is not only timely but essential for resolving several outstanding questions in kagome metals.

Fortunately, the emergence of the novel nematic chiral SC state is not tied to specific details of the order parameters, but instead is based on a physically transparent **OM-chirality coupling mechanism**. This mechanism is clearly established based on the Ginzbuerg-Landau free energy; see **SI H**

Furthermore, the present **OM–chirality coupling mechanism** is not tied to a particular pairing mechanism. We demonstrate that this mechanism causes the chiral SC state in the **spin-fluctuation-mediated superconductors**, by performing the RPA analysis for the 12-site kagome metal model; see **SI D.**

In addition, the robustness of the **OM–chirality coupling mechanism** has been carefully verified based on the analysis of the **three-dimensional $xz + yz$orbital model** (see **SI E**), and the nonlinear gap equation analysis to investigate the chiral SC sate below

T_c.(see **SI K**).

In summary, the novel LC-induced nematic chiral SC state is a direct consequence of the **OM–chirality coupling mechanism**, rather than an artifact of any "systematic bias" arising from the presence of 'many competing orders.' Therefore, the proposed mechanism has a degree of universality and may serve as a useful guidepost for future experimental and theoretical studies of superconductivity.

Below, we briefly explain the **OM-chirality coupling mechanism** and **Spin-fluctuation-mediated pairing**, both of which are the main findings added in the revised manuscript:

【OM-chirality coupling mechanism】

In **SI H,** we newly derive the Ginzburg–Landau free-energy that describes the LC-induced chiral superconducting transition:

$$\boldsymbol{F}_{\mathbf{OM-ch}} = -\bar{\boldsymbol{M}}_{orb}(|\boldsymbol{\Delta}_{d-}|^2 - |\boldsymbol{\Delta}_{d+}|^2),$$

where $\bar{M}_{orb}$ has a functional form similar to M_{orb}. This simple free energy gives rise to the '**OM-chirality coupling mechanism**,' whereby the chiral SC state is stabilized via a bilinear coupling between the LC-induced orbital magnetization M_{orb} and the angular momentum of the Cooper pairs. This discussion is clearly presented in detail in **SI H**. Importantly, this mechanism becomes even more significant deep in the SC phase ($T \ll T_c$); see **SI K**.

Thus, the present OM-chirality coupling mechanism provides a clearer physical picture and offers improved insight into the LC-induced chiral superconductivity.

【Spin-fluctuation-mediated pairing】

In the revised manuscript, we newly added the RPA analysis for the repulsion pairing state (see **SI D** for details). We obtain a nodal s-wave SC state as the leading eigenvalue. In addition, the second eigenvalues correspond to a twofold-degenerate d-wave SC state. The resulting gap functions are shown in **Figs. 5(b) and (c).** The η dependence of the eigenvalues is presented in **Fig. 5(d)** ($\phi = 0$) and in **Fig. 5(e)** ($\phi = -0.024$). These results indicate that chiral SC state is realized over a relatively wide parameter region. This result strongly supports the generality of the LC-induced chiral SC mechanism.

Action

We have added the following important discussions:

・**SI H: Derivation of the GL free energy: OM-chirality coupling** (on page 17 in the SI)：We have clarified the universality and robustness of the LC-induced chiral SC mechanism by elucidating the underlying "**OM–chirality coupling mechanism**.", which is free from the fine tuning of model parameters.

・**SI D: RPA analysis of kagome lattice Hubbard model** (on page 6 in the SI): The OM–chirality coupling robustly stabilizes a chiral SC state, even when nonlocal repulsive spin-fluctuation-mediated pairing interactions are taken into account.

・**SI K: Analysis of nonlinear gap equation in the LC+BO phase** (on page 26 in the SI): The OM–chirality coupling becomes more significant inside the SC phase.

○ Comment 3-3

In total, with regard to publication in Nature Communications, I take a rather neutral stand, slightly leaning against publication in Nature Communications. I simply cannot assign too much relevance to mean field proposals that, in particular in the case of a system as complicated as kagome metals, involve a cascade of mean fields (nematicitiy at higher energy, orbital currents at intermediate energy, superconductivity at low energy. I do, however, acknowledge that the authors' suggestion of s + (d+id) has not yet appeared in the literature.

○ Reply 3-3

We thank the reviewer for acknowledging the novelty of the proposed s +chiral d-wave superconducting state. As we explained above, this LC-induced novel SC state is reliable because it is a direct consequence of the **OM–chirality coupling mechanism**.

Here, we briefly discuss the **hierarchy of energy scales** in kagome metals, which forms one of the fundamental bases of the present theory.

【Energy hierarchy of BO, LC and SC】

In kagome metals, the LC and BO order parameters are expected to $\phi \sim 30\text{meV}$ and $\eta \lesssim \phi$ experimentally. In contrast, the SC gap Δ is more than an order of magnitude smaller than η, ϕ. This clear separation of energy scales justifies the present approximation. The values of η and ϕ are chosen within experimentally suggested ranges. The current understanding of the hierarchy of energy scales, based on both theoretical and experimental studies, is discussed in detail in **SI F** ("**Hierarchy of Energy Scales in Kagome Metals**").

Based on the above, introducing the high-energy-scale LC and BO orders as constants—on the basis of previous experimental and theoretical findings—and investigating low-energy superconductivity is a well-justified and productive strategy. Accordingly, we concentrated our efforts on superconductivity and identified key physical insights.

Furthermore, as we have discussed in **Reply 2-7**【Emergence of attractive interaction: RG theory】, we analyze a kagome metal model including onsite repulsion (U) and bond-order interaction (v) using parquet renormalization-group (RG) theory. Within the RG framework, LC, BO, and SC are treated on an equal footing. In **SI F** the experimentally observed hierarchy of temperature scales, "$T_{BO} \gtrsim T_{LC} \gg T_{s\text{-}SC} \sim T_{d\text{-}SC}$", is naturally reproduced. Based on this hierarchy, it is therefore well justified to treat the LC and BO orders as essentially constant within the SC phase.

Action

To clarify the above important discussion on the energy hierarchy, we have added **SI F**: "**Hierarchy of Energy Scales in Kagome Metals**" (on page 14 in the SI).

i. *Emergence of the s wave SC and d wave SC instabilities derived from the parquet RG*,

ii. *Magnitude of the bond order* ϕ,

iii. *Magnitude of the LC order* η,

iv. *Magnitude of the superconducting gap.*

○ Comment 3-4

Two minor points:

1. The manuscript needs a detailed spellcheck. Example: Fermi -> Fermi

○ **Reply 3-4**

Thank you very much for the helpful advice. We have addressed this point.

○ **Comment 3-5**

2. There is highly related work on the microscopic formation of orbital currents in kagome medals, which the authors might want to consider: https://arxiv.org/pdf/2506.01648, https://arxiv.org/abs/2405.09451.

○ **Reply 3-5**

We sincerely thank you for bringing these highly important theoretical works (arXiv:2506.01648 and arXiv:2405.09451) to our attention. These studies clearly emphasize the crucial role of off-site interactions in establishing the CL order.

Especially, these studies investigate the phase diagram of BO, LC, and superconductivity on an equal footing within a single Hamiltonian, and therefore constitute important and complementary contributions to the present work.

In contrast, in this work, we analyze the 12-site unit-cell kagome model to investigate the emergence of an exotic SC state under the strong nonperturbative influence of the 2×2 LC and BO.

Action

We have cited arXiv:2506.01648 and arXiv:2405.09451 as Refs. [27] and [28], respectively, in the revised manuscript.

Our ultimate goal is to achieve a fully unified understanding of BO, LC, and SC on the same footing, and the above papers represent an initial step toward this goal. Therefore, we have added the following sentences to the final paragraph of the SUMMARY section:

(on page 19 in the main text) "In this study, we examined exotic SC states under the strong influence of LC+BO by approximating ϕ and η as constants, justified by the hierarchy of energy scales $\phi, \eta \gg \Delta$. However, the achievement of a unified understanding of BO, LC, and SC, as initiated in Refs. [27, 28], remains the ultimate objective."

【Summary of the Replies to All Reviewers #1-#3】

We would like to once again express our sincere gratitude for all the reviewers' careful reading and insightful comments. We have carefully addressed all constructive suggestions and, guided by these valuable comments, performed important additional calculations, thereby significantly enhancing the robustness and clarity of the manuscript. As a result, we believe that the overall clarity and completeness of the work have been significantly improved. The main points of improvement are summarized below:

1. **Introduction of a minimal yet realistic three-dimensional $xz + yz$ orbital model and its theoretical analysis:** We reveal that the LC-induced chiral SC state is robust against the band reconstruction due to the substantial inter-orbital hybridization. (see Figs. S5–S7 in SI E).
2. **Spin-fluctuation–exchange mechanism based on a local repulsive interaction (U) model:** As an example of a nonlocal pairing mechanism, we performed the RPA analysis to verify the universality of the present LC-induced chiral SC mechanism (see Fig. 5 and Fig. S4 in SI D).
3. **Predominant impurity effect on the nematic chiral SC**: We show the impurity-induced crossover from a chiral d-wave–type gap function to an s-wave–type gap function (see Fig. S11 in SI J).
4. **Clear physical interpretation of the chiral SC mechanism—the OM-chirality coupling mechanism:** The chiral SC state with finite Cooper-pair angular momentum is stabilized by the LC-induced effective $M_{\rm orb}$ (see Fig.S10 and SI H).
   This mechanism becomes even more significant deep in the SC phase (see SI K).
5. **Nonlinear SC gap equation below T_c**,: The present LC-induce chiral SC state persist inside the SC phase. Furthermore, a drastic change in the gap function for $T \ll T_c$ can be realized (see Fig. S12 in SI K).

In light of this growing body of experimental and theoretical work, we believe that the investigation of the LC-induced exotic SC state is both timely and essential. Given recent experimental reports of novel superconducting phenomena—such as the zero-field superconducting diode effect and half-vortex states—the present "OM–chirality coupling mechanism for chiral SC state" provides valuable insights into many outstanding and still mysterious open questions. Therefore, we believe that the present manuscript is well suited for publication in *Nature Communications*.

List of Changes Made in the Revised Manuscript

- All textual revisions in the revised manuscript are indicated in red.
- Due to improvements in the xz-orbital tight-binding model ($t = -0.8,\ t' = 0.16t$), several figures presenting numerical results have been updated. These changes lead to quantitative improvements in the results, while the qualitative conclusions remain unchanged.
- In order to provide thorough and transparent responses to the reviewers' comments, we have newly prepared Supplemental Information (SI A–SI K).
- Introduction of a realistic three-dimensional $xz + yz$ orbital model and its theoretical analysis (Figs. S5–S7 in SI E).
- Analysis of the spin-fluctuation–exchange mechanism based on a local repulsive interaction (U) model (Fig. 5 and Fig. S4 in SI D).
- Investigation of impurity effects, in particular the impurity-induced crossover from a chiral d-wave–type gap function to an s-wave–type gap function (Fig. S11 in SI J).
- Analysis of the nonlinear superconducting gap equation below $T_{\rm c}$, with particular emphasis on the drastic change in the superconducting gap function deep in the superconducting phase (Fig. S12 in SI K).
- Development of a clear physical interpretation of the chiral superconductivity mechanism based on the OM-chirality coupling mechanism (Fig.S10 and SI H).

Fig. R1. DOS of AV_3Sb_5 (A=Cs,Rb,K): d-orbital weight of the density-of-states in ab-initio three-dimensional 30 orbital tight-binding model. For all A = Cs, Rb, K, xz+yz orbitals exhibit the largest weight around the Fermi level (E=0).

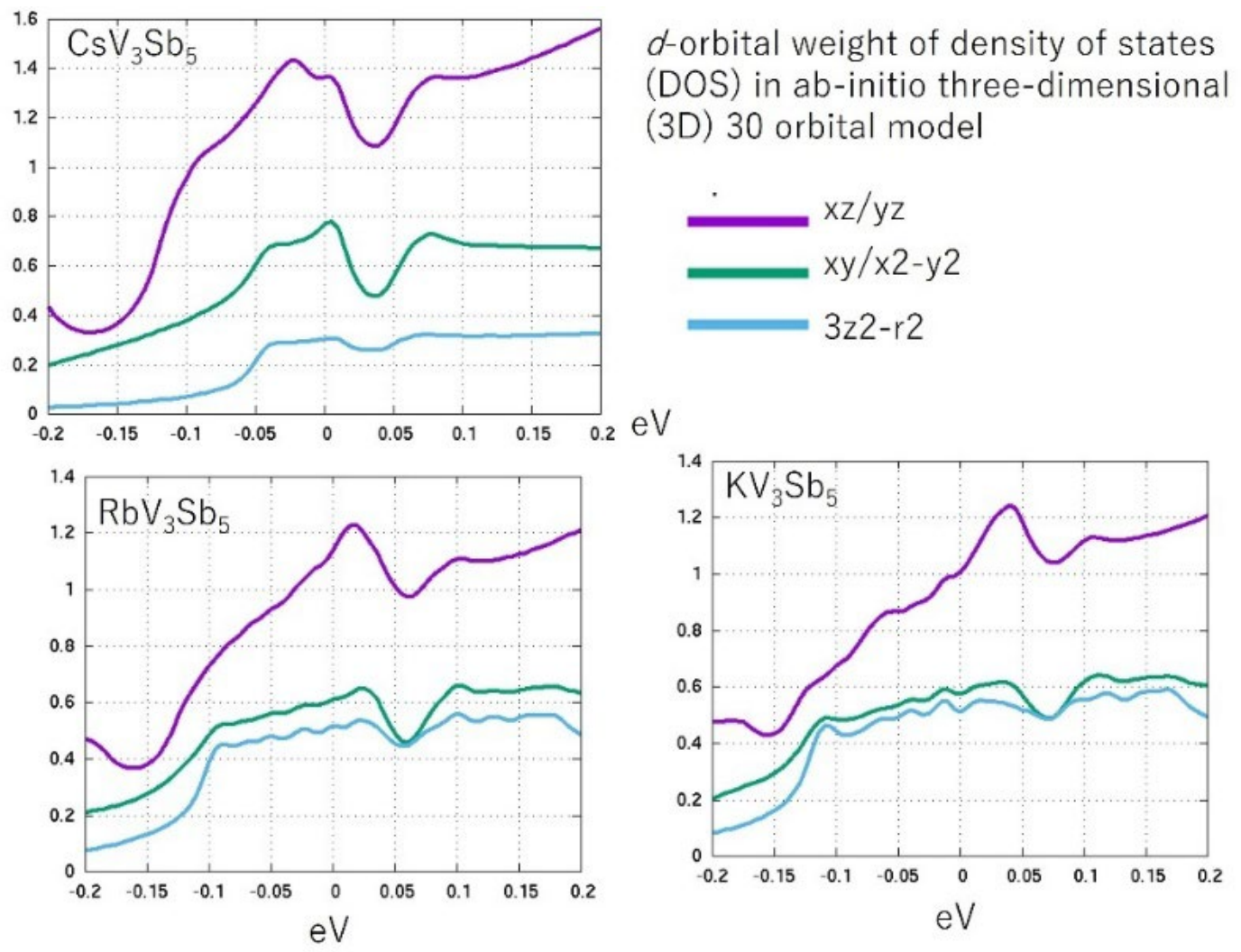

Fig.R2. DW equation analysis for 3D 30 orbital model: Eigenvalue of the density-wave equation based on the three-dimensional 30 orbital Hubbard model for A=Cs at T=0.04. Here, $E_{vHS} - \mu = -0.048$eV for n=31, and $E_{vHS} - \mu = -0.079$eV for n=31.2. In both cases, nearly commensurate BO is obtained.

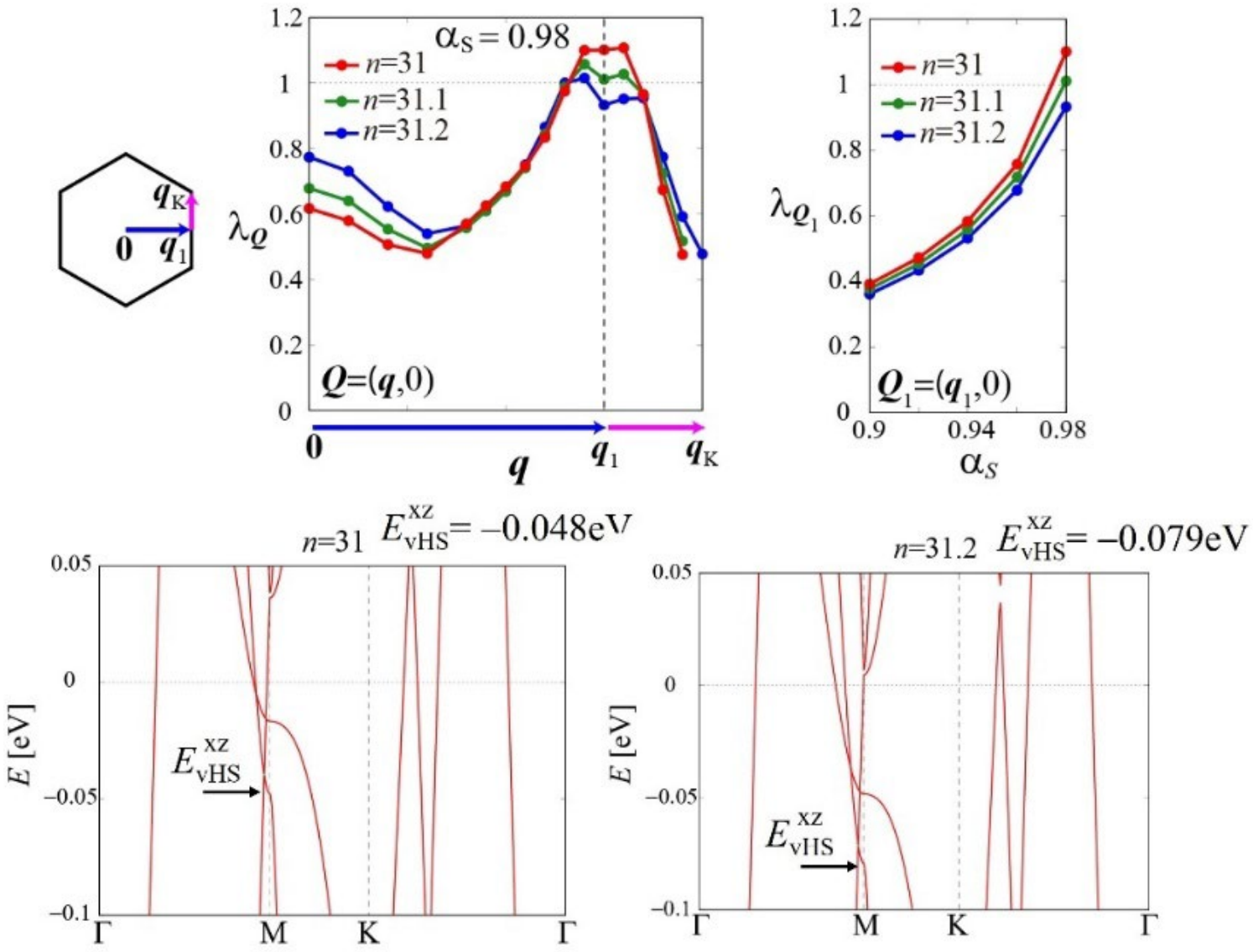

Fig.R3. Gap equation eigenvalue λ_Q: Eigenvalue of the SC gap equation λ_Q as a function of the Cooper pair momentum **Q** at T=0.8meV: (left) η=0.032 and ϕ=0; (right) η=0.032 and ϕ= − 0.032. In both cases, λ_Q exhibits the peak at **Q**=**0**.

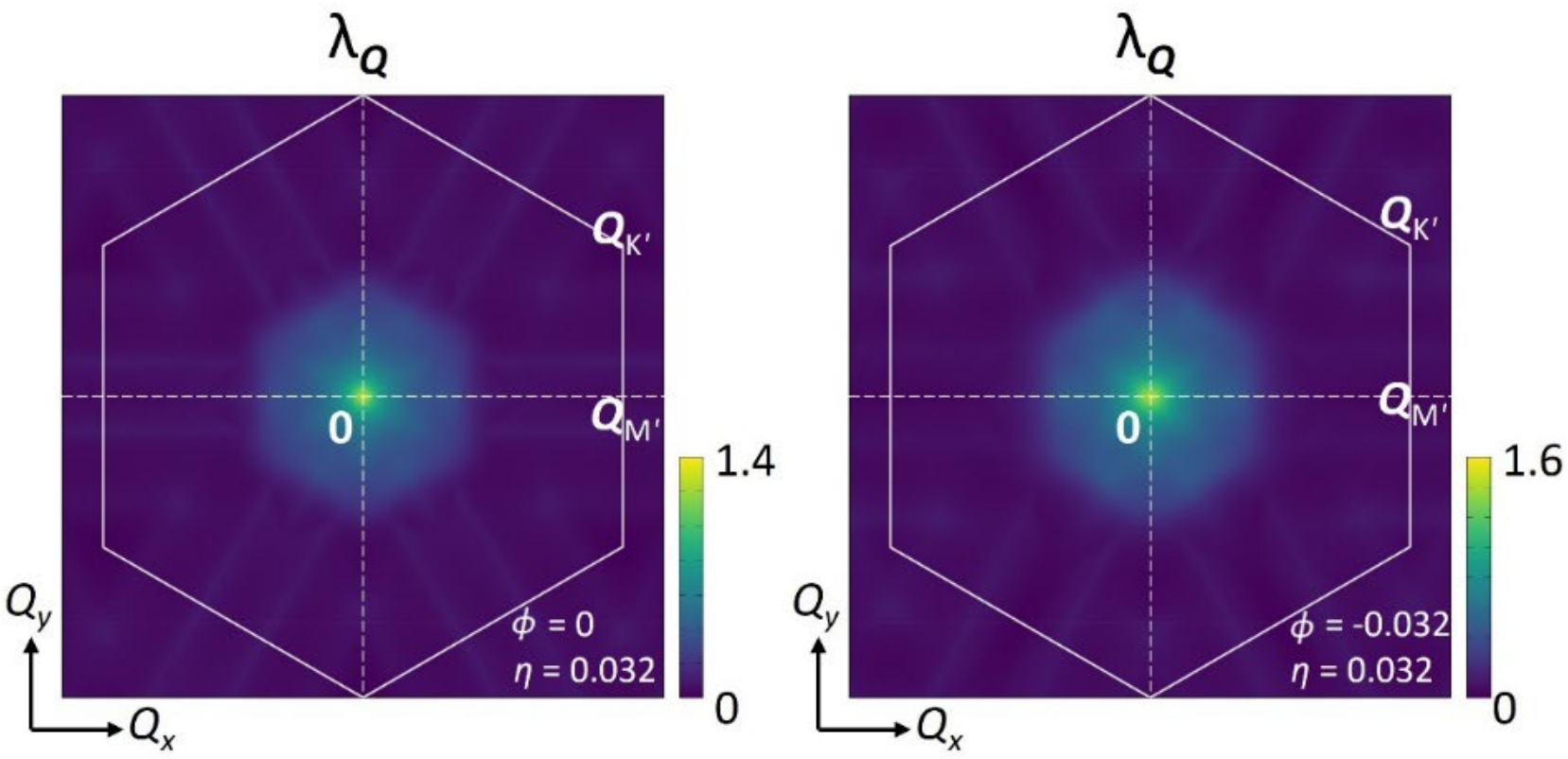

Reply to the Comments by Reviewer #1

〇 Comment 1-0

The authors have addressed most of my previous questions and concerns satisfactorily. I find that the manuscript has improved significantly, both in terms of technical content and physical clarity. In particular, the introduction of the orbital-magnetization–chirality coupling mechanism provides a more transparent understanding of how loop-current order selects the chiral superconducting state.

With the following points clarified, I would be supportive of publication in Nature Communications.

〇 Reply 1-0

We sincerely thank the Reviewer for the positive evaluation of our revised manuscript and for the encouraging comments on the improvement in both its technical content and physical clarity. We are particularly grateful that the Reviewer found the orbital-magnetization–chirality coupling mechanism useful for clarifying how the loop-current order selects the chiral superconducting state.

Below, we provide our point-by-point responses to the remaining comments raised by the Reviewer.

〇 Comment 1-1

1. Energy scale of the OM–chirality coupling

One of the key advances in the revision is the identification of the orbital magnetic moment as the driving force for the splitting between different chiral superconducting channels. I find this mechanism very interesting.

It would be helpful if the authors could provide an estimate of the magnitude of this splitting, for example by comparing the chiral splitting (e.g., ¥lambda_{d-}-¥lambda_{d+}) to the overall pairing strength or to T_c. This would give a clearer sense of how dominant this mechanism is relative to the underlying pairing interaction.

In addition, it would be useful to further clarify the relation between orbital magnetization and Berry curvature in this context, i.e., whether the orbital magnetization should be

viewed as the primary driver of chirality selection, or as a phenomenological manifestation of the underlying Berry-phase structure.

○ Reply 1-1

We sincerely appreciate your highly significant and fundamental comments, which have helped us further strengthen our central conclusion regarding the "OM-chiral coupling mechanism" and its close connection to the LC-induced Berry phase. Since the explanation in the previous version of the manuscript was insufficient, we have added further clarification in the revised manuscript.

Our responses are provided below.

1. **Validity of the OM-chiral coupling mechanism:**

In Fig. S10(a), we previously showed the η dependence of the uniform orbital magnetization $M_{\rm orb}$ at $T = 0.8$meV, but the values were given in arbitrary units. In the revised manuscript, we now express $M_{\rm orb}$ per V atom in units of μ_B. The unit conversion is described in Ref. [31]. For $\eta, \phi \sim 10$meV, we obtain $M_{\rm orb} \sim 10^{-4}\,\mu_B$. Since $M_{\rm orb} \propto \eta\,\phi^m$ (m=1,2), $M_{\rm orb}$ increases to $\sim 10^{-3}\,\mu_B$ for $\eta, \phi \sim 20$ meV. In kagome metals, $T_{\rm BO}, T_{\rm LC} \lesssim 100$K. Thus, taking $\eta, \phi = 10$–20 meV, and the expression in Eq. (S18), $M_{\rm orb}$ is estimated to be 10^{-4}–$10^{-3}\,\mu_B$. We note that for $n = 11.84$, which is used in the present calculation, the vHS energy is located approximately 0.1 eV away from E_F. Therefore, the temperature dependence of $M_{\rm orb}$ below $T < 100$K is moderate.

In Fig. S10(b), we also show the η-dependence of the splitting of the gap-function eigenvalues in the chiral superconducting state,

$$\Delta\lambda = \lambda_{d+} - \lambda_{d-},$$

at $T = 0.8$meV. Note that $\Delta\lambda$ is proportional to $\bar{M}_{orb}$. The superconducting transition temperature at $\eta = 0$ is $T_{c0} = 0.8$meV, so the calculation is performed at $T \sim T_{c0}$. From this figure, $\Delta\lambda \sim 0.05$ for $\eta, \phi \sim 10$meV. Since $\bar{M}_{\rm orb} \propto \eta\phi^m$ (m=1,2), $\Delta\lambda$ increases to ~ 0.5 for $\eta, \phi \sim 20$meV.

We now discuss the qualitative consistency between these two quantities, $M_{\rm orb}$ and $\Delta\lambda \propto \bar{M}_{orb}$. In the LC phase, the energy splitting between conduction-electron states expanded in terms of orbital angular momentum (such as between $|\,l_z = \pm 1\rangle$) is estimated as $\Delta E \sim |M_{\rm orb}|/N(0)$, where N(0)~1eV^{-1} is the density-of-states. Thus, $\Delta E =$ 0.1-1 meV for φ, η = 10–20 meV. This energy splitting is large enough to produce a substantial eigenvalue splitting, $\Delta\lambda \gtrsim 0.1$, between the two chiral d-wave superconducting states with $l_z = \pm 2$, for $T_c \sim \Delta_{\rm SC} \sim 1$ meV.

2. Discussion of Berry phase and Berry curvature

The imaginary part of the off-diagonal components in Eq. (3), ($\pm b$), is proportional to the Berry phase acquired by Cooper pairs when they undergo pair hopping among sites A, B, and C. We would like to emphasize that this Berry phase not only increases in proportion to the current order η, as shown in Figs. 3(a) and 3(b), but also becomes larger at lower temperatures, as shown in Fig. 3(b). A mechanism for the emergence of chiral superconductivity based on the Berry phase and Berry curvature has also been discussed in Ref. [55].

As shown in Eqs. (5) and (6), $\Delta\lambda \propto |b|$, which is consistent with the discussion of the OM-chirality coupling energy $F_{\mathrm{OM-ch}}$ introduced in Eq. (S19). However, the former argument is specific to the kagome lattice model, whereas the latter provides a more general perspective that extends beyond the kagome lattice model. We expect that the importance of the latter viewpoint will be clarified further in future studies.

Action:

To incorporate the above discussion into the revised manuscript, we added the unit to the vertical axis of Fig. S10a in Sec. H of the SI.
We then inserted the following explanatory text.

○ On page 9 of the main text:

In the present model, $|M_{\mathrm{orb}}| = 10^{-4}$–$10^{-3}$ μ_B per V atom for φ, η = 10–20 meV; see Sec. H of the SI [30]. The energy splitting between states with finite angular momenta, such as |l_z = ±1⟩, is then estimated as $\Delta E \sim M_{\mathrm{orb}}/N(0)$ = 0.1–1 meV, where $N(0) \sim 1$ eV^{-1} is the density of states. The estimated ΔE is sufficiently large to induce a significant splitting between λ_{d+} and λ_{d-} when $T_c \sim 1$ meV.

○ On page 23 of the SI:

From the numerical results in Fig. S10a and the analytic expression in Eq. (S18), we estimate $|M_{\mathrm{orb}}| = 10^{-4}$–$10^{-3}$ μ_B per V atom for φ, η = 10–20 meV [31].

○ Comment 1-2

2. Role of fluctuations and consistency of the mechanism

In the revised manuscript, the loop-current and bond-order states are assumed to form at temperatures significantly higher than the superconducting transition and to be pinned by the lattice.

Given this, I would appreciate some clarification on the role of fluctuations in the superconducting pairing mechanism. In particular, what type of fluctuations are implicitly involved in generating the effective pairing kernel in this low-temperature regime, and how this is consistent with the assumed rigidity of the loop-current and bond-order background.

○ **Reply 1-2**

We sincerely appreciate your very fundamental and constructive comments on the quantum fluctuations responsible for the pairing mechanism in kagome metals.

In Ref. [8], we found that BO fluctuations make an important contribution to Cooper-pair formation. Relatedly, in the FeSe model, we verified that the BO instability remains sizable, with $\lambda_{\rm BO} \sim 0.9$, even for $T \ll T_{\rm BO}$; see Fig. 6 of Ref. [58]. This analysis suggests that BO fluctuations can remain strong even for $T \ll T_{\rm BO}$ in kagome metals.

The Cooper-pair attraction V_X in the spin-singlet channel mediated by $X = \mathrm{BO}$ fluctuations is expressed, using the structure factor $f_q^X(k)$ of the BO order, as

$$V_X(k,k') = g_X \frac{[f_q^X(k) f_q^X(-k')]}{1-\lambda_X(q)}, \qquad q = k' - k,$$

where g_X is the coupling constant, and $\lambda_X(q)$ is the eigenvalue corresponding to the structure factor $f_q^X(k)$. Here, the relation $1-\lambda_X(q) \sim \mid T - T_X \mid$ is realized as we have explained in Ref. [58]. In the following, we consider $X = \mathrm{BO}$ or LC.

$V_{\rm BO}$, which originates from BO fluctuations, mediates an attractive pairing interaction. In Ref. [8], based on the Hubbard model, we derived $g_{\rm BO}$, $\lambda_{\rm BO}(q)$, and $f_q^{\rm BO}(k)$ contained in $V_{\rm BO}$ using an electron-correlation theory beyond the mean-field approximation. For $U \sim 1.2$eV, we obtained $\lambda_{\rm BO} \sim 0.88$ and $g_{\rm BO} = g_{\rm back} + g_{\rm um} \sim 3$eV. Since $g_{\rm BO} \gg U$, this result shows that even moderate BO fluctuations can give rise to a strong attractive interaction.

On the other hand, $V_{\rm LC}$, which originates from LC fluctuations, mediates a repulsive interaction because the structure factor $f^{LC}(k)$ is an odd function, and therefore $[f^{LC}(k) f^{LC}(-k')] < 0$, as revealed in Ref. [64]. In Ref. [29], we microscopically derived $\lambda_{\rm LC}(q)$ and $f_q^{\rm LC}(k)$, but we have not yet been able to derive $g_{\rm LC}$ owing to technical difficulties. In Ref. [64], $g_{\rm LC}$ was introduced as a model parameter. If $g_{\rm LC} \sim O(U)$, moderate LC fluctuations would give rise to a strong superconducting interaction.

For simplicity, assuming $\lambda_{\rm BO} \sim \lambda_{\rm LC}$, we obtain

$$|V_{\rm LC}/V_{\rm BO}| \sim |g_{\rm LC}/g_{\rm BO}|.$$

Thus, if $g_{\rm LC} \sim g_{\rm BO}$, LC fluctuations and BO fluctuations would contribute comparably to superconductivity. However, if $g_{\rm LC} \ll g_{\rm BO}$ $(\sim O(U))$, the contribution of LC fluctuations to superconductivity would likely be limited. The microscopic derivation of $g_{\rm LC}$ is therefore crucial for exploring the possibility of superconductivity mediated by LC fluctuations. At present, however, this issue remains unresolved and constitutes an important direction for future research.

Action:

To briefly introduce the above discussion, we added the following sentence.

○ On page 17 of the main text:

The present LC-induced chiral SC mechanism can emerge for pairing driven by either attractive or repulsive interactions. The attractive interaction may originate from BO fluctuations [8], in addition to phonons. In contrast, the repulsive interaction may arise from spin fluctuations as well as from the LC-fluctuation mechanism [64] owing to the odd-parity LC form factor. (Relatedly, spin-loop-current (sLC) fluctuations can mediate attraction even though they originate in the spin channel, owing to the odd-parity sLC form factor [65].) The pairing strength generated by these fluctuations, which is governed by both proximity to quantum criticality and the magnitude of the coupling constant, remains an important theoretical issue in kagome metals.

○ **Comment 1-3**

3. Topological characterization of the superconducting state

In Reply 1-6, the authors discuss the topology in terms of the winding number of the superconducting gap function. While this is a useful diagnostic, it appears somewhat different from the standard definition of topology in superconductors based on the BdG Chern number.

It would be helpful if the authors could clarify the relation between these two characterizations, and under what conditions the winding number they compute is equivalent to the BdG Chern number. A brief discussion would be sufficient.

○ **Reply 1-3**

We sincerely appreciate your very fundamental and constructive comments on the

topological characterization of the SC state. In the original paragraph discussing the relation between the winding number and the Chern number, the underlying assumptions and conditions were not explained sufficiently. We have therefore substantially expanded the explanation in the revised manuscript, as detailed below.

In the previous manuscript, we briefly stated that the winding number w is equal to the Chern number C in the fully gapped superconducting state. However, this statement is valid only under the condition that the pairing is purely intraband and when the Berry curvature of the normal-state bands is neglected.

More explicitly, if the pairing is purely intraband, the BdG Hamiltonian can be decomposed into independent 2×2 blocks in the band basis. For each band b, the skyrmion number is given by $w_b = (1/4\pi)\int dk_x\, dk_y\, \mathbf{n}_\mathrm{b}\, (\partial_{k_x}\mathbf{n_b} \times \partial_{k_y}\mathbf{n_b})$, where the unit vector is defined as $\mathbf{n}_{b,k} = (\Delta_{b,k}', \Delta_{b,k}'', \mu - \varepsilon_{b,k})/(|\Delta_{b,k}|^2 + (\mu - \varepsilon_{b,k})^2)^{1/2}$. w_b always takes integer values and represents how many times $\mathbf{n}_{b,k}$ covers the unit sphere over the Brillouin zone. Because $\mathbf{n}_{b,k}=(0,0,\pm 1)$ sufficiently far away from the Fermi surface, while $\mathbf{n}_{b,k} = (\cos\theta_{b,k}, \sin\theta_{b,k}, 0)$ on the Fermi surface, w_b is determined by the winding number of $\theta_{b,k} = \arctan(\Delta_{b,k}''/\Delta_{b,k}')$ along the Fermi surface, taken anticlockwise (clockwise) for the electron-type (hole-type) Fermi surface.

Also, the Chern number for band b given by $C_b = (1/2\pi)\int dk_x\, dk_y\ \Omega^b_k$, where Ω^b_k is the Berry curvature of the occupied Bogoliubov bands. By analyzing 2×2 BdG Hamiltonian, we can show that $w_b = C_b$ =integer. Therefore, the total Charn number is equal to the total skyrmion number.

This discussion is restricted to the contribution from the superconducting gap, and possible additional contributions from the Berry curvature of the normal-state bands in the LC phase are neglected.

Action:

The revised paragraph is as follows.

○ On page 8 of the main text:

In the chiral d-wave state, $\Delta_k \equiv \Delta_k' + i\Delta_k''$ is a complex-valued function of k. Figures 2b and 2c show the obtained $|\Delta_k|$ and $\theta_k \equiv \arctan(\Delta_k''/\Delta_k')$ on the Fermi surface, respectively, for $\eta = 0.029$ at T = 0.8 meV. Here, $|\Delta_k|$ exhibits a sizable k dependence owing to the phase differences among Δ_l (l = 1–12). The vector representation of θ_k for a single Fermi pocket is shown in Fig. 2d. w_1 (w_2) denote the winding number of the SC gap on the electron-type (hole-type) FS, evaluated in the anticlockwise (clockwise) direction. The total winding number, $w = w_1 + 2w_2$, coincides with the skyrmion number

$(1/4\pi)\Sigma_b \int dk_x\, dk_y\, \mathbf{n}_b\, (\partial_{kx} \mathbf{n}_b \times \partial_{ky} \mathbf{n}_b)$, with the unit vector $\mathbf{n}_{b,\mathbf{k}} = (\Delta_{b,k}', \Delta_{b,k}'', \mu - \varepsilon_{b,k})/(|\Delta_{b,k}|^2 + (\mu - \varepsilon_{b,k})^2)^{1/2}$. This quantity is equivalent to the Chern number $C = (1/2\pi)\Sigma_b \int dk_x\, dk_y\, \Omega^b{}_k$ of the occupied Bogoliubov bands, when the Berry curvature of the normal-state bands in the LC phase is neglected.

List of changes made in the revised manuscript

All changes made in the revised manuscript have been explained in the replies above. The revised parts of the text are marked in red in the manuscript. Regarding the figures, we added the unit to the vertical axis of Fig. S10 **a**.

Reply to the Comments by Reviewer #1

○ Comment

The authors have addressed my remaining questions satisfactorily. The revised manuscript is significantly improved in clarity and technical content, especially through the added discussion of the orbital-magnetization–chirality coupling mechanism.

I find that the main concerns raised in the review process have now been adequately addressed. I support publication in Nature Communications.

○ **Response:**

We sincerely thank Reviewer #1 for the positive evaluation of our revised manuscript and for confirming that the remaining questions have been satisfactorily addressed. We are particularly grateful for the reviewer's appreciation of the improved clarity and technical content, including the added discussion of the orbital-magnetization–chirality coupling mechanism. We also deeply appreciate the reviewer's support for publication in Nature Communications. Finally, we would like to express our sincere gratitude for the reviewer's many constructive and insightful comments throughout the review process, which have greatly helped us improve the manuscript.

List of changes made in the revised manuscript

(i) We have shortened the Abstract to bring it within the 150-word limit.

(ii) We have revised Figs. 2, 4, 5, and 10 by adding legends identifying each line.

(iii) We have added the Figshare DOI to the Data Availability statement and included it as a reference.
